# Model-Based Geostatistical Mapping of the Prevalence of *Onchocerca volvulus* in West Africa

**DOI:** 10.1371/journal.pntd.0004328

**Published:** 2016-01-15

**Authors:** Simon J. O’Hanlon, Hannah C. Slater, Robert A. Cheke, Boakye A. Boatin, Luc E. Coffeng, Sébastien D. S. Pion, Michel Boussinesq, Honorat G. M. Zouré, Wilma A. Stolk, María-Gloria Basáñez

**Affiliations:** 1 Department of Infectious Disease Epidemiology, School of Public Health, Faculty of Medicine (St Mary’s Campus), Imperial College London, London, United Kingdom; 2 MRC Centre for Outbreak Analysis and Modelling, Department of Infectious Disease Epidemiology, Imperial College London, London, United Kingdom; 3 Natural Resources Institute, University of Greenwich at Medway, Chatham, Kent, United Kingdom; 4 Lymphatic Filariasis Support Centre, Department of Parasitology, Noguchi Memorial Institute for Medical Research, University of Ghana, Legon, Ghana; 5 Department of Public Health, Erasmus MC, University Medical Center Rotterdam, Rotterdam, The Netherlands; 6 UMI 233, Institut de Recherche pour le Développement (IRD) and University of Montpellier 1, Montpellier, France; 7 African Programme for Onchocerciasis Control (APOC), World Health Organization (WHO), Ouagadougou, Burkina Faso; 8 London Centre for Neglected Tropical Disease Research, Department of Infectious Disease Epidemiology, Imperial College London, London, United Kingdom; University of Queensland School of Veterinary Science, AUSTRALIA

## Abstract

**Background:**

The initial endemicity (pre-control prevalence) of onchocerciasis has been shown to be an important determinant of the feasibility of elimination by mass ivermectin distribution. We present the first geostatistical map of microfilarial prevalence in the former Onchocerciasis Control Programme in West Africa (OCP) before commencement of antivectorial and antiparasitic interventions.

**Methods and Findings:**

Pre-control microfilarial prevalence data from 737 villages across the 11 constituent countries in the OCP epidemiological database were used as ground-truth data. These 737 data points, plus a set of statistically selected environmental covariates, were used in a Bayesian model-based geostatistical (B-MBG) approach to generate a continuous surface (at pixel resolution of 5 km x 5km) of microfilarial prevalence in West Africa prior to the commencement of the OCP. Uncertainty in model predictions was measured using a suite of validation statistics, performed on bootstrap samples of held-out validation data. The mean Pearson’s correlation between observed and estimated prevalence at validation locations was 0.693; the mean prediction error (average difference between observed and estimated values) was 0.77%, and the mean absolute prediction error (average magnitude of difference between observed and estimated values) was 12.2%. Within OCP boundaries, 17.8 million people were deemed to have been at risk, 7.55 million to have been infected, and mean microfilarial prevalence to have been 45% (range: 2–90%) in 1975.

**Conclusions and Significance:**

This is the first map of initial onchocerciasis prevalence in West Africa using B-MBG. Important environmental predictors of infection prevalence were identified and used in a model out-performing those without spatial random effects or environmental covariates. Results may be compared with recent epidemiological mapping efforts to find areas of persisting transmission. These methods may be extended to areas where data are sparse, and may be used to help inform the feasibility of elimination with current and novel tools.

## Author Summary

Onchocerciasis is a neglected tropical disease caused by *Onchocerca volvulus* and transmitted by *Simulium* blackflies. Operational between 1975 and 2002, the Onchocerciasis Control Programme in West Africa (OCP) was one of the first large-scale intervention programmes ever implemented, and was successful in controlling the disease as a problem of public health importance. Currently, national control programmes in the former OCP and the World Health Organization African Programme for Onchocerciasis Control (APOC) are shifting their focus from morbidity control to elimination of the infection where feasible through community-directed treatment with ivermectin (CDTI). The level of endemicity (measured as infection prevalence) prior to control interventions has been shown to be an important determinant of the feasibility of elimination by CDTI. We present the first pre-control Bayesian geostatistical map of infection (microfilarial) prevalence in the OCP with the objectives of: a) identifying important environmental covariates which help to estimate prevalence in areas without observed survey data; b) understanding the distribution of areas of initially high endemicity, where the current CDTI strategy may be insufficient to achieve elimination goals and intensified CDTI or novel/complementary tools may be required; c) re-estimating the initial numbers of persons at-risk and with onchocerciasis in 1975.

## Introduction

Human onchocerciasis (river blindness) is a parasitic infection caused by the filarial nematode *Onchocerca volvulus*. Onchocerciasis belongs to the group of neglected tropical diseases (NTDs). In January 2012, the London Declaration on NTDs [1], inspired by the World Health Organization (WHO) Road Map to overcome the impact of these diseases [2], committed to sustain, expand and extend the necessary supply of drugs and other interventions to help control onchocerciasis throughout Africa, and achieve elimination in selected African countries by 2020, with global elimination/eradication targeted by 2040 [1, 2, 3]. The life cycle of *O*. *volvulus* depends on transmission by biting flies of the genus *Simulium*. Persistent infection can lead to gradual loss of visual acuity, eventually leading to blindness, and to the development of a variety of severe and debilitating skin lesions [4, 5]. The infection is also a cause of excess human mortality both indirectly through blindness and directly through (still poorly understood mechanisms caused by) heavy infection in sighted individuals [6, 7].

**Fig 1:**
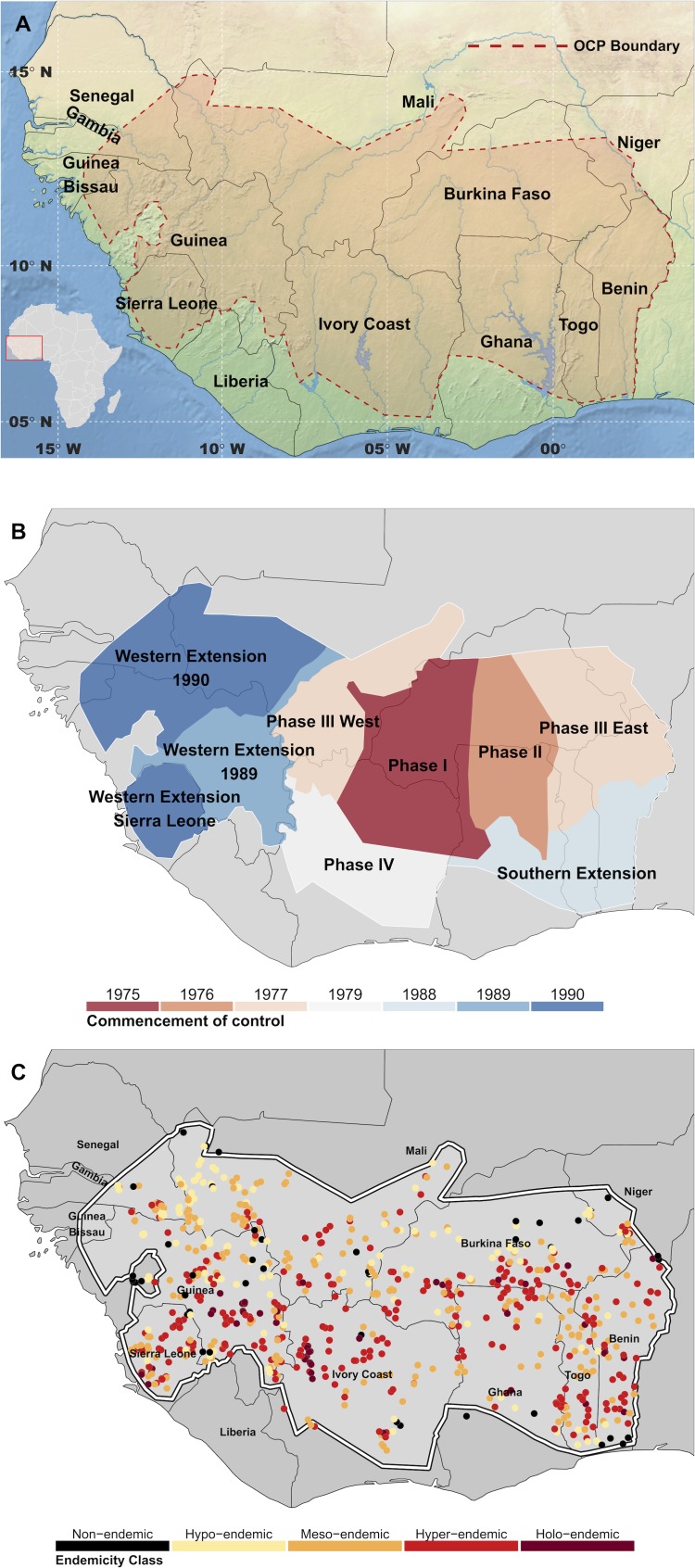
Maps of the Onchocerciasis Control Programme area. A) Map showing the area covered by the Onchocerciasis Control Programme (OCP) in West Africa. The red dashed line delineates the limit of anti-vectorial and/or anti-parasitic activities carried out by the OCP between 1975 and 2002. B) Map of the operational phases of the OCP. Until 1987 the method of control was almost exclusively anti-vectorial, through larviciding of vector breeding habitats. From 1987 ivermectin was used extensively, and almost exclusively in the Western Extension zones. C) Locations of the village surveys which met the criteria for inclusion in this study. A total of 737 survey sites were selected, from 5,817 surveys. The survey data displayed are coloured by endemicity class of crude microfilarial prevalence in the population aged ≥5 years. For the cut-off values of microfilarial prevalence corresponding to each endemicity category see Main Text and legend of Fig 3B.

Onchocerciasis has previously been highly prevalent across much of sub-Saharan Africa and in foci in Latin America and the Yemen [8]. Under the umbrella of the WHO, the Onchocerciasis Control Programme in West Africa (OCP) deployed large-scale antivectorial and/or antiparasitic interventions against onchocerciasis from 1975 to 2002 to control the disease in initially 7 (original area) and finally 11 (including extension areas) West African countries (Fig 1A) [9]. The implementation of vector control, by weekly larviciding of *Simulium damnosum* s.l. breeding sites for a period at least as long as the estimated longevity of the parasite, led to the interruption of transmission across large swathes of the OCP area [10, 11]. From 1990 onwards, vector control began to be stopped in those basins that had received at least 14 years of larviciding, and in which the epidemiological and entomological trends were satisfactory [12]. However, because of invasion by infective flies, some rivers on the border of the original area continued to be treated beyond the hypothetical period of 14 years, and up to the time the extension areas were set up. Although onchocerciasis ceased to constitute a public health problem in the original OCP area, transmission persisted in others, and towards the end of the OCP in 2002, special intervention zones were established to continue larviciding and/or to intensify distribution of the microfilaricidal drug ivermectin [13].

Currently, national control programmes in the former OCP and the WHO African Programme for Onchocerciasis Control (APOC) are shifting their focus from morbidity control to elimination of the infection where feasible through community-directed treatment with ivermectin (CDTI). Using 15–17 years of annual (or biannual) ivermectin distribution in the absence of vector control, elimination has been documented in some OCP foci in Mali and Senegal [14, 15], and some APOC foci in Nigeria [16], providing proof-of-principle for the goals set by the WHO for elimination of onchocerciasis in selected African countries by the year 2020 [2]. However, a key question that needs urgent investigation is where else will it be feasible to achieve the 2020 goals in the former OCP and APOC areas using current intervention strategies, and if not, by when these goals could be achieved, or what other (novel or complementary) strategies are required.

Among the main determinants of the duration of ivermectin-based control and the feasibility of its elimination is the initial, pre-control, level of endemicity [17, 18]. Endemicity levels are usually categorised in terms of the community prevalence of infection with *O*. *volvulus* microfilariae, with the community microfilarial load (CMFL) sometimes also used. (A highly non-linear relationship exists between infection prevalence and infection intensity, such that at high infection prevalence the CMFL may vary widely.) CMFL is calculated as the geometric mean number of microfilariae per skin snip in a cohort of adults aged 20 years and over, and is an indicator of the intensity of infection [19]. CMFL is a more accurate indicator of the true epidemiological situation in a population than prevalence, as for the reasons mentioned earlier there can be substantial variability in CMFL values at high prevalences. However, prevalence data are more widely available. Microfilarial prevalence is positively correlated with transmission intensity, measured in terms of the annual transmission potential (ATP)—the yearly number of infective larvae of *O*. *volvulus* potentially received by a person maximally exposed to simuliid bites in the same community, although the relationship between these two variables is also strongly non-linear [20, 21].

Therefore, and to further understanding of the feasibility of onchocerciasis elimination, we embarked on the task of mapping the distribution of the baseline microfilarial prevalence in the former OCP area. Recent modelling studies [18, 22], have indicated that reaching the operational elimination thresholds suggested by APOC [23] may be feasible, with an annual ivermectin distribution strategy, in areas with a baseline microfilarial prevalence less than 60% (the prevalence value above which there is hyperendemicity), provided that high levels of therapeutic coverage and treatment compliance are maintained. By contrast, in initially highly hyperendemic areas (prevalence of 80% or above), biannual ivermectin distribution or deployment of complementary strategies (e.g. focal vector control if possible; delivery of anti-wolbachial therapies [24], or others [25]) may be necessary. Mapping of infection prevalence is of importance not only for targeting current or novel control efforts appropriately, but also, once local elimination has been achieved or is deemed possible to achieve within proposed time frameworks, to predict areas at risk of recrudescence from geographically contiguous areas still harbouring infection. Finally, mapping the baseline prevalence can help to determine the population at risk and infected prior to the inception of control interventions, essential denominators to calculate the initial burden of disease and its changes as a result of such interventions [26].

The production of continuous prevalence maps is increasingly using model-based geostatistics (MBG) [27], often employing Bayesian inference (B-MBG) for spatial prediction and robust characterisation of uncertainty surrounding those predictions [28]. Detailed prevalence maps have been presented for a variety of directly- and indirectly-transmitted human helminthiases, e.g. [29–32], and vector-borne diseases, e.g. [28, 33–35], including lymphatic filariasis [36]. Zouré et al. (2014) recently presented a model-based geostatistical map of the prevalence of onchocerciasis in countries covered under APOC [37], based on nodule prevalence data obtained from Rapid Epidemiological Mapping of Onchocerciasis (REMO). This map marks substantial progress in mapping the pre-control distribution of onchocerciasis in APOC areas, but does not consider the potential influence of environmental covariates affecting onchocerciasis distribution, particularly in areas still lacking ground-truth data. In the study presented in this paper, we map the pre-control distribution of onchocerciasis—based on skin snip data for microfilarial prevalence—in the area of West Africa formerly covered by the OCP, whilst also extending the geostatistical methods to consider and incorporate important environmental explanatory covariates.

The overall aims of this study are to: a) present the first geostatistical map of human onchocerciasis (microfilarial) prevalence at the inception of the former OCP in West Africa; b) identify important environmental covariates that aid estimation in locations for which no data were collected by the OCP; c) to characterise areas where high transmission occurred prior to control (areas of initially high onchocerciasis endemicity), and d) estimate the number of people living in the OCP area that were at risk and infected with *O*. *volvulus* prior to the commencement of the control interventions.

## Methods

The methods used in the epidemiological surveys of the OCP have been previously described [38]. At each survey a complete census of the village was conducted, and approximately 84% of persons enumerated in the census were examined [39]. The countries participating in the OCP signed a memorandum of agreement that covered all issues pertaining to the operations and included clearance for epidemiological, parasitological, and ophthalmological surveys. Additionally, a committee consisting of the Chief of Units of OCP ensured that the plans and the methodology of work were correctly followed by the technicians in the field. Communities were free to participate in the taking of skin snip samples. Therefore, our study satisfies the requirements for ethical clearance within the memorandum and the data were provided by BAB (former and last director of the OCP).

### Onchocerciasis Prevalence Data

Relevant survey data were extracted from the OCP epidemiological database, which contains 5,816 surveys conducted in 2,581 (geo-referenced) villages across all operational phases of the OCP in the 11 participating countries in West Africa (see Fig A within S1 File for locations of all village sites in the OCP epidemiological database). There were 9 operational phases (summarised in Table 1 and depicted in Fig 1B). A total of 737 villages had parasitological surveys that met the inclusion (pre-control) criteria (see S1 File Text A ‘Data description and pre-control data selection criteria’ and Fig B therein). Prevalence ranged between 0 and 94.8% with a mean of 51.7% and a median of 56.4%. With the exception of one village, numbers examined per village ranged from 23 to 828 with a median of 171. One village had just 5 individuals examined (prevalence of 40%) but this represented 100% of the recorded population for this village. The geographical distribution and endemicity of the selected villages are shown in Fig 1C, with the endemicity classes defined as non-endemic/sporadic endemicity <10%; hypoendemic onchocerciasis: ≥10% and <35%; mesoendemic: ≥35% and < 60%; hyperendemic: ≥ 60% and < 80%, and highly hyperendemic (or holoendemic): ≥ 80% microfilarial prevalence. Although in the OCP it was standard epidemiological practice to adjust microfilarial prevalence by age and sex according to the OCP reference population [40], in this study we used crude microfilarial prevalence because counts of persons examined were not available in the data by age cohort. The correlation coefficient between crude and adjusted prevalence was 0.973 (determination coefficient 0.947, Fig C in S1 File), therefore the use of crude prevalence was not expected to bias results.

**Table 1:** Number of pre-control input data points by operational phase of the Onchocerciasis Control Programme in West Africa and the commencement date of vector larviciding operations.

Phase Name		Start of control	Type of intervention*	No. of pre-control surveys (n = 737)
I		February 1975	Larviciding	115
II		January 1976	Larviciding	61
III East		March 1977	Larviciding	87
III West		February 1977	Larviciding	27
IV		March 1979	Larviciding	47
Southern Extension		February 1988	Larviciding	83
Western Extension	1989	March 1989	Ivermectin	–
	1990	April 1990	Ivermectin	317
	Sierra Leone	March 1990	Larviciding + Ivermectin	–

* The types of intervention are informed by [9–12].

### Geographical Limits of the Mapping

We restricted our mapping to the geographic extent visible in Fig 1A, which encompasses the OCP and immediate surrounding areas in West Africa. Our ground-truth data points, consisting of parasitological survey data from the OCP epidemiological database, were located almost entirely within the boundaries of the OCP, as depicted in Fig 1C (see also Fig D in S1 File for a heat map of distance of map pixels to the nearest input, ground-truth data point within the study area). The OCP boundaries themselves were largely defined according to the primary objectives of eliminating blinding onchocerciasis (in savannah areas), subsequently protecting the original area (of 654,000 km^2^, spread over Burkina Faso, south-eastern Mali, south-western Niger, and northern Côte d’Ivoire, Ghana, Togo and Benin) from reinvasion by infective flies [9, 12], which led to the extensions described in Table 1. River basins in which the blackfly vectors of onchocerciasis can breed were identified by the OCP for the different species of the *Simulium damnosum* complex [41, 42]. We let the northerly limits of the mapping extend to extremely arid, desert conditions where transmission of onchocerciasis is known not to occur, but we do not generate pseudo-absence points in these areas; instead, we allow the model to generate prevalence values based on the environmental factors associated with the data and distance from known (ground-truth) data points. The easterly limit extends to the fringes of Nigeria, where control is under the auspices of APOC and we do not consider estimation beyond these areas. (See Text B ‘Parasitological survey methods and geographical limits of the mapping’ in S1 File.)

### Climatic and Environmental Data

An ensemble of explanatory environmental and climatic covariates which were deemed useful in the estimation of microfilarial prevalence was identified building on the methodology presented in a previous mapping study for another filarial infection [36]. Initial inclusion was broad and partly based on knowledge of parasite and vector ecology (e.g. distance of villages to fast flowing rivers), as well as variables considered to affect their distribution and life cycle (e.g. climatic data). Climatic data were extracted from WorldClim Global Climate Data, http://www.worldclim.org [43]. Hydrologically-derived variables were extracted and calculated from the Hydro1K database [44, 45], and an alternative set of hydrologically-derived variables was extracted from the Rivers of Africa dataset (derived from HydroSheds) [46], which includes alternative metadata about the stream lines. Normalized Difference Vegetation Index (NDVI) data were extracted from the Global Inventory Modeling and Mapping Studies (GIMMS) dataset for the years 1981 to 1986 [47–49] (the earliest data available in this dataset). Data layers, generated from the MODIS instrument on board the Terra satellites, were also used. These layers included land surface temperature (day and night), land cover classification, NDVI and enhanced vegetation index (EVI) [49–53]. A layer of vegetation type based on physiognomy and floristic composition from White’s Vegetation Map of Africa was also extracted and used [54]. We initially tested layers for altitude derived from digital elevation models (DEMs) from different mapping projects, which were extracted from the Global 30 Arc-Second Elevation (GTOPO30) DEM [55, 56] and the Global Land One-km Base Elevation Project (GLOBE) [57]. Where necessary, the original data sources were assembled together to form contiguous coverage of the study area, re-projected to the output map projection (Lambert Azimuthal Equal Area; Fig E in S1 File), re-sampled to 5km x 5km (25 km^2^, the resolution of the output map), cropped to the borders depicted in Fig 1A and, finally, those cells that overlay inland water bodies were masked as they represented no data values. Each pixel of the output map (*n* = 112, 295), therefore, had a value for each of the environmental covariates. The values of these covariates at each survey village were sampled from these layers. The initial ensemble of covariates tested and the data sources are summarised in Table 2 and further details of the processing of these datasets are described in Text C of S1 File ‘Processing of environmental covariates’. Maps of the covariates that were included in the model are presented in Fig F.1 and Fig F.2 of S1 File Text D ‘Maps of environmental covariates’.

**Table 2:** Sources of spatially-referenced explanatory environmental covariate data considered in this study.

Dataset	Layers Included	Pixel resolution	Coverage	Projection	Datum	Temporal coverage	Source	Version	Ref
WorldClim	Min Temp, Max Temp, Mean Temp, Precipitation, Bioclim 1–19	1km	Global	Geographic	WGS84	1950–2000	http://www.worldclim.org/bioclim	Version 1.4 (release 3)	[43]
HYDRO1k Elevation Derivative Database	Slope, Aspect, Compound Topographic Index, Stream lines^1^*, Drainage basins, Elevation^2^, Flow direction, Flow accumulation	1km	Africa	Projected	Lambert Azimuthal equal-area	NA	https://lta.cr.usgs.gov/HYDRO1K	NA	[44,45]
Rivers of Africa (Derived from HydroSHEDS)	Stream lines^1^,Strahler order, River gradient, River type	Shapefile	Africa	Geographic	WGS84	2000	http://www.fao.org/geonetwork/srv/en/metadata.show?id=37333	First Edition	[46]
Global Inventory Modeling and Mapping Studies (GIMMS)	Normalised Difference Vegetation Index^3^	8km	Global	Projected	Albers equal-area conic	1981–2006	http://www.landcover.org/data/gimms/index.shtml *	NA	[47–49]
MODIS Vegetation Indices (MOD13Q1)	250m 16 days Normalised Difference Vegetation Index,250m 16 days Enhanced Vegetation Index	250m	Global	Projected	Sinusoidal	2000-Present	https://lpdaac.usgs.gov/products/modis_products_table/mod13q1	Version 005	[50]
MODIS Land Surface Temperature & Emissivity (MOD11A2)	8-Day daytime 1km grid land surface temperature,8-Day night time 1km grid land surface temperature	1km	Global	Projected	Sinusoidal	2000-Present	https://lpdaac.usgs.gov/products/modis_products_table/mod11a2	Version 005	[51]
MODIS Land Cover Type Yearly (MCD12Q1)	Land Cover Type 2 (UMD)	500m	Global	Projected	Sinusoidal	2001–2012	https://lpdaac.usgs.gov/products/modis_products_table/mcd12q1	Version 051	[52,53]
UNESCO (White's) Vegetation Map of Africa	Vegetation type map	30 arc seconds	Africa	Geographic	WGS84	1968–1983	http://geonetwork.grid.unep.ch/geonetwork/srv/en/metadata.show?uuid=cc2fbd96-ae4c-4593-910f-c103b1b51299	NA	[54]
Global 30 Arc-Second Elevation (GTOPO30)	Altitude^2^	30 arc seconds	Global	Geographic	WGS84	1996-Present	http://webmap.ornl.gov/wcsdown/dataset.jsp?ds_id=10003	NA	[55,56]
Global Land One-km Base Elevation Project (GLOBE)	Altitude^2^	30 arc seconds	Global	Geographic	WGS84	NA	http://www.ngdc.noaa.gov/mgg/topo/globe.html	Version 1.0	[57]
Gridded Population of the World (GPW V3.0)	Population density,Population count	5km	Global	Geographic	WGS84	1990-Present	http://sedac.ciesin.columbia.edu/data/set/gpw-v3-population-density	Version 3	[69]
Global Rural-Urban Mapping Project (GRUMP) V1.0	Rural-Urban extents	Shapefile	Global	Geographic	WGS84	1990-Present	http://sedac.ciesin.columbia.edu/data/dataset/grump-v1-urban-extents	Version 1	[71]

^1^Environmental covariate layers for Log_10_ distance to river and distance to river were derived from these vector shapefiles.

^2^Multiple digital elevation models from these sources were tested and the best performing variables were selected for further testing. See S1 File for details.

^3^This dataset is currently unavailable from the Global Land Cover Facility website.

### Temporal Trends in the Data

Although the baseline survey data used in this study were collected over a period of 16 years (1974–1990), exploratory inspection of pre-control prevalence boxplots by year showed that there was no significant temporal trend. It was, therefore, deemed unnecessary to include the effect of time as these data were assumed to be representative of the pre-control endemic equilibrium situation at the beginning of each OCP phase and commencement of intervention by river basin. Time-varying changes in the environmental covariates were also treated as negligible. The WorldClim dataset uses interpolated data averaged over 50 years, whilst the MODIS data, representing 10-day means, were averaged by ecological quarter (1st: Dec–Feb, 2nd: Mar–May, 3rd: Jun–Aug, 4th: Sep–Nov) across the entire duration of available data at the time of analysis (February 2000–July 2013). There was no discernable non-linear trend in NDVI from MODIS vs. NDVI from the Advanced Very High Resolution Radiometer, which has a much earlier temporal coverage (from 1981) but a coarser spatial resolution (64km^2^ pixels). Therefore, the long-term climatic and environmental data were implicitly assumed to be representative of the average conditions present in 1975–1990 even if portions of the covariate data had been collected prior or subsequent to this date.

### Spatial Dependence in the Data and Model-Based Geostatistical Analysis

A geostatistical model (used to develop a continuous map of pre-control microfilarial prevalence across the OCP area) is simply a Generalised Linear Mixed Model (GLMM) with environmental covariates to account for the fixed effects, a spatial term that accounts for the spatial correlation in the data, and a random effect term that accounts for the non-spatial variation in the data, yielding a Generalised Linear Spatial Model (GLSM).

If we assume that the number of individuals found positive for *O*. *volvulus* microfilariae at location *x*_*i*_ is *Y*_*i*_ out of the total number of people examined by skin snip at such location, *n*_*i*_, then *Y*_*i*_ is a binomial random variable, *Y*_*i*_ ∼ *Bin*(*n*_*i*_, *p*_*i*_) where *p*_*i*_ is the proportion of individuals positive for skin microfilariae at location *i*.

**Fig 2:**
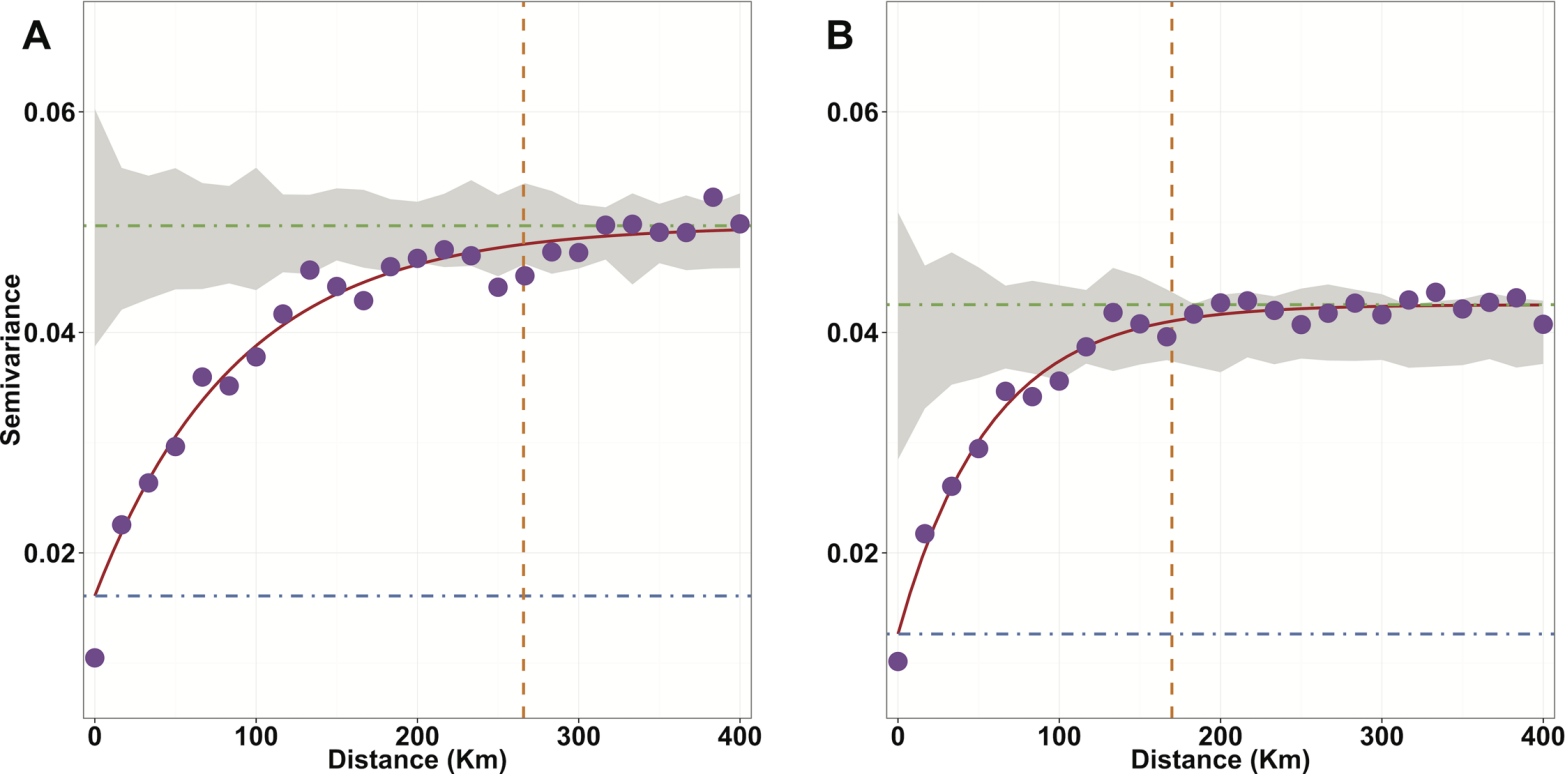
Semi-variogram analysis of spatial trend in the data. A) Empirical variogram assuming a constant mean trend across the study region (i.e. without the use of environmental covariates). B) The spatial trend present in the data after accounting for the effects of explanatory environmental covariates by incorporating a spatially-varying mean trend surface. The purple dots are the mean semivariance values between all pairs of villages with separation distance contained within a discrete distance bin, with the mid point of each bin used to locate the dots on the *x*-axis. The red solid line is the fitted theoretical variogram model. The dash-dot blue horizontal lines represent the nugget variance *τ*^2^, encompassing sources of non-spatial variation. The dash-dot green horizontal lines represent the sill variance, *σ*^2^, which is the estimated variance of the spatial process *S*(⋅). The dashed orange vertical lines are the range at which the variance asymptotically reaches 95% of its sill value and is given by −ln(0.05)*ϕ*, where ϕ is the range parameter. Beyond this value the covariance between locations does not significantly depend on separation distance. The grey shaded areas are the Monte Carlo envelopes of variance expected by chance, obtained by randomly reassigning the values of microfilarial prevalence at data locations and calculating the empirical semivariogram. The empirical variogram (A) mostly lies outside this region and, therefore, there is therefore a significant spatial trend present in the data. The trend in semivariogram (B) is fitted to the residuals resulting after adjusting for the set of linear predictors of the environmental covariate model. The reduction in range parameter, compared to the mean trend shows that the environmental covariates account for some (but not all) of the observed spatial variation. As some of such variation still lies outside of the Monte-Carlo envelope of spatially random data, there is still a significant spatial trend.

An exploratory tool widely used in geostatistics is the semivariogram **[**58**]**. Under the assumption of spatial dependence, there is a relationship between the variance of the difference between pairs of random variables (in this study microfilarial prevalence) and their separation distance, *d*. The empirical variogram represents the relationship between that separation distance, binned into intervals, and the mean semivariance of infection prevalence between all pairs of locations within each distance bin (Fig 2A). Firstly, we fitted a theoretical variogram model to an empirical variogram controlling for the environmental covariates (Fig 2B)—using an exponential covariance function—to generate sensible prior values to use for the spatial component of the Bayesian model (see section on ‘Prior Specification‘ below). Second, we used these in the GLSM to fit a model of linear predictors (the environmental variables as fixed effects), conditional on some stochastic process *S*(⋅), i.e., the unobserved, underlying spatial process (which enters the model as a location-specific random offset in the linear predictor). Following [27], modifying the notation in [36], and conditional on *S*(⋅), the responses *Y*_*i*_/*n*_*i*_ at locations *x*_*i*_, for *i* = 1,…,*k* (*k* = 737 survey villages) are mutually independent random variables whose conditional expectations, *μ*_*i*_ = *E*[(*Y*_*i*_ / *n*_*i*_) | *S*(⋅)], are given by,

$$h(\mu_{i})=S(x_{i})+\sum_{j=1}^{p}\beta_{j}d_{j}(x_{i}),$$ (1)

where *μ*_*i*_ is the expected value of microfilarial prevalence at location *x*_*i*_; *S*(*x*_1_),…,*S*(*x*_*k*_) are the unobserved random variables for the *i* = 1,…,*k* locations derived from the spatial process, *S*(⋅); *d*_*j*_(*x*_*i*_) represents the value of the *j*^*th*^ environmental variable at location *x*_*i*_; *β*_*j*_ is the regression coefficient of the *j*^*th*^ variable, and the link function is denoted by *h*(⋅). For binomial data the link function is the logit function, i.e. *h*(*μ*) = log[*μ*/(1−*μ*)].

The spatial signal *S*(⋅) has expectation *E*[*S*(*x*)] = 0, variance *Var*[*S*(*x*)] = *σ*^2^ and covariance function,

$$C(d)=\left\{\begin{array}{ll} \sigma^{2}+\tau^{2} & :d=0\\ \sigma^{2}\rho (d) & :d>0 \end{array}\right\},$$ (2)

where *σ*^2^ is the ‘sill’ or maximum variance of the spatial process (composed of the sum of the partial sill―the spatial variance―and the nugget variance, *τ*^2^―the non-spatial variance (representing sources of non-spatial variation in true prevalence such as measurement error derived from the skin snip method or unmeasured village-specific demographic or socio-economic factors, among others)―*d* = ‖*x* − *x*′‖ is the Euclidean distance between *x* and *x*′, and *ρ*(*d*) is the correlation function which controls how the variogram increases from the intercept to its asymptote. Initial fitting of semivariogram models suggested that the exponential correlation function gave a good fit to the data for both a mean trend without covariates (Fig 2A) and after controlling for the effect of environmental covariates (Fig 2B). The exponential correlation function is given by,

ρ(d)=exp(−d/ϕ), (3)

and the semivariance *γ*(*d*) of the exponential model with a nugget effect is given by,

$$\gamma (d)=\tau^{2}+\sigma^{2}\left[1-\text{exp}\;\left(-d/\phi \right)\right]$$ (4)

where *ϕ* is the *range parameter*, determining the rate at which the dependence between *S*(*x*) values decays with distance. Taking −ln(0.05)*ϕ* gives the distance at which the semivariance asymptotically reaches 95% of its sill value. The specification of this correlation function determines the smoothness of the underlying spatial surface (see section on ‘Prior Specification*‘* below). We denote the vector of *j* = 1,…, *p* regression parameters as *β* and the parameters of the correlation function as *θ*. We can use this model specification and the data to make predictions about locations of interest which may not have been sampled, but for which values of the regression coefficients are available (environmental covariates).

Continuing with the notation of [27], we may specify a vector of prediction at *target* locations, *T* = *T*(S*), where $S*=S(x_{1}^{*}),\ldots ,S(x_{m}^{*})$ are the values of *S*(⋅) at the x^*^ un-sampled locations where predictions are required (in this study, the 25km^2^ grid covering the study area that contains observed values of covariates at all *m* = 112, 295 prediction locations). It is not possible to calculate the predictive distribution [S*|*Y*,*β*,*θ*] directly, therefore we use Markov chain Monte Carlo (MCMC) simulation to generate samples from the conditional distribution, [(*S*,S^*^)|*Y*] = [*S* | *Y*][S^*^|*S*,*Y*]. Because S^*^ is conditionally independent of *Y* and *β* given *S*(⋅), we generate values by direct simulation from the conditional distribution [S^*^ | *S*, *θ*]. (See [27] for further details and extensions to this framework.) The modelling framework was implemented using the geostatistical modelling software from the package *geoRglm (Version 0*.*9–2)* [59] of the R statistical environment for computing (*Version 3*.*0*.*1*) [60].

### Initial Selection of Environmental/Climatic Covariates

A systematic Bayesian step-wise procedure was used to select the best set of uncorrelated predictor covariates for use in the geostatistical model. This method builds upon that of Austin and Tu [61] by fitting univariate Bayesian GLSM models for both linear and quadratic functional forms of each of the initial predictor covariates. We tested model fit by means of a *K*-folds cross validation, with *K* = 1,…, 10. The data were randomly partitioned into a training set, consisting of approximately 90% of the data ($N_{T_{K}}$ = 663), whilst the remaining 10% ($N_{V_{K}}$ = 74) were held-out and used in the validation exercises. In each *K*-fold a different set of input data points was held out, such that each of the 737 data points appeared in one and only one of the *K-*fold validation data set. For this reason, $N_{V_{K=10}}$ comprised 71 data points and $N_{T_{K=10}}$ 666 training data points (see Text E ‘*K*-folds model validation’ and Fig G in S1 File for locations of data points in each *K*-fold). Each model was then independently fitted against the 10 training datasets. The Deviance Information Criterion (DIC), which is a Bayesian measure of model fit [62], was used to compare how well each covariate was able to estimate prevalence at a set of held-out validation locations. Ten datasets were chosen as a compromise between number of bootstraps and computational time.

For both linear and quadratic functional forms of all of the input covariates, using each bootstrap dataset in turn we calculated the DIC for that model. For each covariate, the functional form yielding the lowest DIC was used to select either linear or quadratic forms for further evaluation. The covariates were then ranked by their resulting DIC values (lowest to highest) and an iterative selection procedure applied to select the best set of uncorrelated covariates. In the first iteration, the overall best-performing covariate (the covariate generating the lowest DIC) was selected and the correlation between values at all data locations between the model with this covariate and with all other covariates was computed. Any covariates with a Pearson’s correlation of > 0.7 were deemed to be collinear and were eliminated from further model selection. The second iteration started with the next best remaining covariate, i.e. the covariate yielding the next lowest DIC after the previous round. Collinear covariates were excluded as before and further iterations continued until all remaining covariates had either been eliminated or included in the set of uncorrelated covariates used to identify the best performing model via a Bayesian step-wise selection procedure.

### Building a Multivariate Model

Given the set of uncorrelated potential predictors identified, we used a standard Bayesian forward step-wise procedure to build the most parsimonious model that provided the best fit to the observed data. We started by selecting the model which had the lowest possible DIC, identified from the previous step. Using this as a baseline model, bivariate models, each using an additional covariate, were run. Whichever, if any, of the new models had the greatest reduction in DIC from the baseline was selected to be the new baseline model, and additional covariates were added to this model. At each step we added the covariate which effected the greatest marginal reduction in DIC. The step-wise procedure stopped when the addition of further covariates did not reduce (or increased) the DIC. Table 3 lists the covariates which were included in the final Bayesian GLSM, their median values and their 75% and 95% Bayesian credible intervals (BCI), respectively containing 75% and 95% of the posterior distribution for each parameter.

**Table 3:** Posterior regression coefficient values and Bayesian credible intervals (BCI) for the explanatory environmental covariates included in the linear predictor model as well as for values of spatial covariance parameters.

Variable	Median	95% BCI	75% BCI
Intercept	0.218	(–0.037, 0.495)	**(0.082, 0.39)**
BIO7: Mean temperature annual range	0.136	(–0.071, 0.371)	**(0.002, 0.267)**
BIO7: (Mean temperature annual range)^2^	-0.087	(–0.22, 0.053)	**(–0.171, –0.008)**
BIO8: Mean temperature of wettest quarter	-0.053	(–0.195, 0.103)	(–0.151, 0.027)
BIO8: (Mean temperature of wettest quarter)^2^	**-0.048**	**(–0.1, –0.002)**	**(–0.076, –0.018)**
BIO15: Precipitation Seasonality	**-0.455**	**(–0.78, –0.143)**	**(–0.638, –0.275)**
BIO15: (Precipitation Seasonality)^2^	-0.134	(–0.34, 0.038)	**(–0.243, –0.015)**
BIO16: Precipitation of wettest quarter	0.013	(–0.192, 0.24)	(–0.115, 0.129)
Compound Topographic Index	0.014	(–0.061, 0.109)	(–0.033, 0.064)
Log_10_(Distance to river+1)	**-0.137**	**(–0.215, –0.054)**	**(–0.188, –0.094)**
Log_10_(Distance to river+1)^2^	**-0.034**	**(–0.061, –0.007)**	**(–0.051, –0.019)**
NDVI (1^st^ quarter: Dec–Feb)	**-0.341**	**(–0.637, –0.035)**	**(–0.511, –0.162)**
NDVI (2^nd^ quarter: Mar–May)	0.201	(–0.204, 0.561)	**(0.001, 0.445)**
NDVI (3^rd^ quarter Jun–Aug)	0.101	(–0.098, 0.279)	**(0.007, 0.229)**
NDVI (4^th^ quarter Sep–Nov)	0.023	(–0.237, 0.304)	(–0.15, 0.167)
*ϕ*, range parameter	**38.265**	**(34.341, 41.248)**	**(36.303, 40.187)**
*σ*^2^, sill	**0.790**	**(0.707, 0.874)**	**(0.744, 0.845)**

Values highlighted in bold denote statistical significance.

### Prior Specification

For each of the *β*_*j*_ environmental regression parameters, a prior distribution was assigned, such that $\beta_{j}∼N\;\left(0,\sigma_{\beta_{j}}^{2}\right)$ and $\sigma_{\beta_{j}}^{2}$ was given a value of 10. Pragmatic priors for the spatial correlation function parameters (Eq 2) were specified after initial investigation by fitting a theoretical variogram, by maximum likelihood estimation, to the data conditioning on a mean trend model specified by the environmental covariates (Fig 2B). Slightly at odds with traditional Bayesian prior selection, the maximum likelihood estimate was used as the mean of the prior for the range parameter *ϕ*, to help the model converge better. The prior was specified as a discretised uniform prior, defined across the interval *ϕ*'−10 < *ϕ* < *ϕ*' + 10 with 1,000 discrete support points, where *ϕ*' is the maximum likelihood estimate. The upper and lower limits of support were set to ensure exploration of a wide prior parameter space. The nugget variance, *τ*^2^―not a parameter estimated by the model―was specified as a relative nugget (i.e. as the proportion of the total variance of the spatial process that is due to non-spatial effects). The relative nugget was computed using the parameter estimates from the theoretical variogram model fitting procedure, giving a value of 0.279. The variance *Var*[*S*(*x*)] = *σ*^2^ was assumed to follow a chi-squared distribution, composed of the sum of squares of random normal deviates (from the mean of this distribution, set to 0), drawn at each data location.

Although initial testing indicated that adequate mixing of most MCMC chains was achieved with a burn-in of less than 1.5×10^5^ iterations, some bootstrap datasets did not converge, so the burn-in length was increased to 1.5×10^6^ iterations to ensure that chains reached the posterior equilibrium distribution. A further 1×10^6^ iterations were run to sample from the posterior distribution with thinning set to store every 500th subsample to reduce autocorrelation between successive subsamples, giving a total of 2,000 samples from the posterior distribution. Text F of S1 File provides further details on ‘MCMC chain diagnostics’ and Fig H and Fig I of S1 File present trace plots of MCMC chains for model parameters in the final predictive model. Text G provides further details on ‘Posterior distributions of model parameters’ and Fig J illustrates their highest posterior density (HPD) plots.

### Model Output

**Fig 3:**
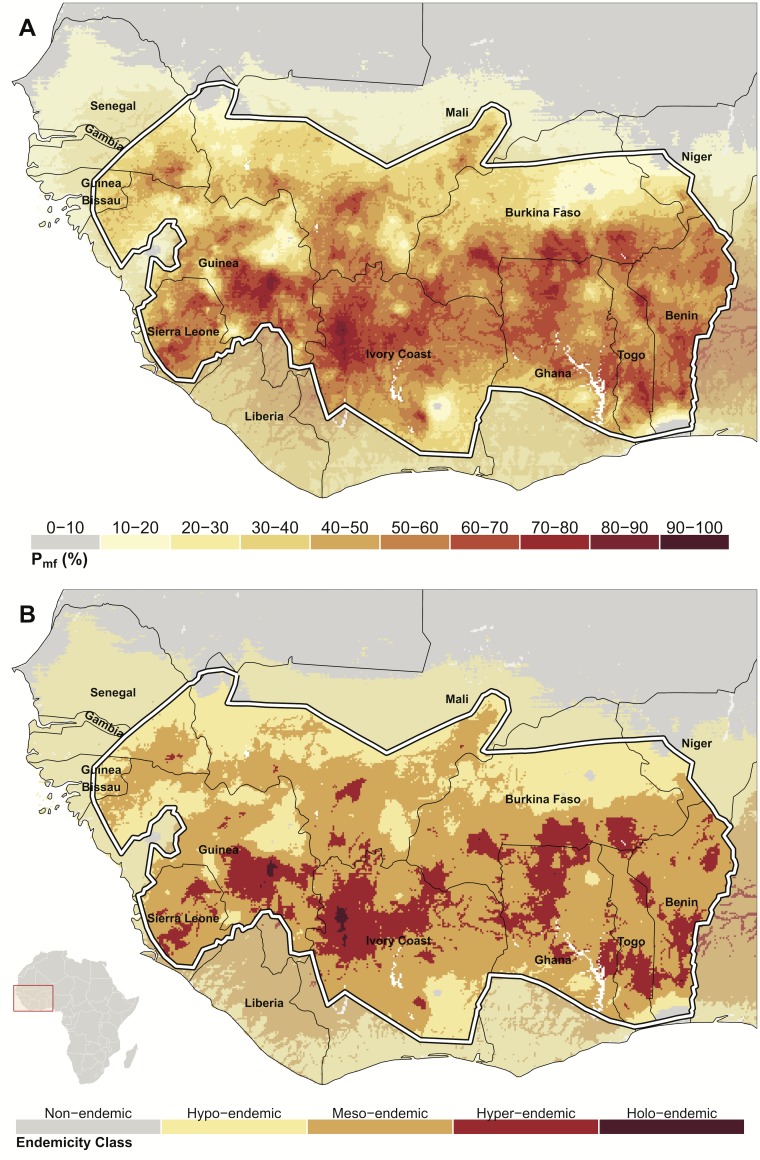
The spatial distribution of onchocerciasis microfilarial prevalence in West Africa. A) The mean of the predictive posterior distribution of microfilarial prevalence in the population aged ≥ 5 years (*P*_*mf*_) for each pixel in the study area. There are no pseudo-absence data points in this model (i.e., pragmatically-generated points of zero-prevalence in areas of known absence of disease) and, therefore, the grey areas in the north of the map, where predictions of prevalence are very low, are entirely determined by the value of the environmental covariates (the closest villages are too distal to exert a spatial effect). The white boundary denotes the limits of the OCP area. B) Endemicity class classifications for the mean of the predictive posterior, based on a modification of the OCP endemicity categories. Areas where prevalence is very low are shown by sub-dividing the hypoendemic class into two categories, a non-endemic/sporadic endemicity class, where microfilarial prevalence is <10%, and a hypoendemic class where prevalence ≥10% but <35%. Other categories are mesoendemic: ≥35% and < 60%; hyperendemic: ≥ 60% and < 80%, and highly hyperendemic (or holoendemic): ≥ 80%.

Model outputs consist of a series of realisations from the posterior distribution of microfilarial prevalence for each map pixel, which may be summarised in a number of ways. The mean of the posterior distribution was taken as a point estimate for each pixel (Fig 3A), and pixels were discretized into broad endemicity classes (Fig 3B) following the endemicity classification for onchocerciasis used by the OCP [63], but also including an extra holoendemic class for very high prevalence areas (previously defined).

**Fig 4:**
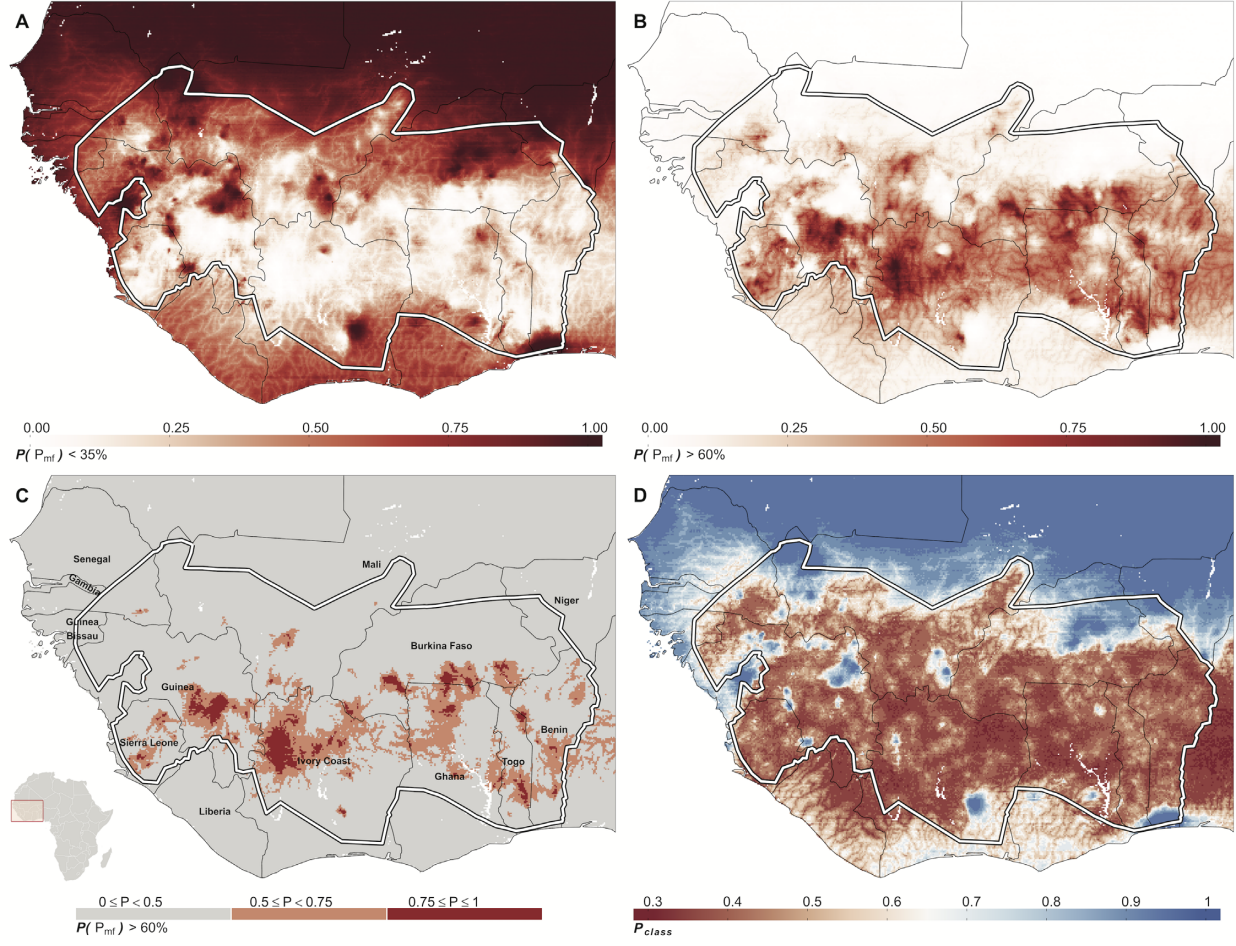
Predictive inference for threshold exceedance and endemicity class membership. A) The posterior predictive probability that microfilarial prevalence for each map pixel is less than 35%. The model output is mapped on a continuous scale from 0 to 1, with dark red representing a high degree of certainty that prevalence does not exceed the threshold of 35%. B) The continuous posterior predictive probability that microfilarial prevalence exceeds the threshold for hyperendemicity of 60%. C) Model output as in Fig 4B but categorised into broad probability intervals. Maps of this nature may have utility for the optimal targeting of current and novel control interventions by programme managers. Light-grey areas are pixels with a probability < 50% of exceeding the 60% threshold; pink pixels denote that the probability of exceeding hyperendemicity lies between 50% and 75%; deep-red colours represent a probability > 75% of exceeding this threshold. D) The probability of each map pixel being in the endemicity class to which it was assigned (*Ρ*_*class*_). With 4 endemicity classes the probability ranges from a minimum of 0.25 to 1, with a value of one meaning that all realisations from the predictive posterior were assigned to the same endemicity class. In all panels the red boundary denotes the OCP limits.

It is also straightforward to derive quantities that may be of operational interest in control programmes, such as threshold probability maps (Fig 4A–4C) or probability of endemicity class classification (Fig 4D). We mapped the predictive probability that prevalence did not exceed the threshold of hypoendemicity (35%), where the burden of onchocerciasis as a public health problem is relatively low. We also mapped the predictive probability that prevalence exceeds the epidemiologically important threshold of hyperendemicity (60%), above which there is a greater than non-linear increase in morbidity experienced by the population, and annual ivermectin distribution may not be sufficient to reach operational elimination thresholds within the WHO proposed timeframes [22]. To map the predictive probability that prevalence does not exceed 35% we approximated from the predictive posterior at each target location (x^*^) by calculating the proportion of realisations (*r*) from the predictive posterior that do not exceed this threshold; $(1/r)\sum_{i=1}^{r}P(0.35<x_{i}^{*})$ at each prediction location x^*^, with *P* taking a value of 0 or 1 depending on whether the threshold was exceeded or not. This surface is depicted in Fig 4A. The continuous probability surface that prevalence exceeds 60% is shown in Fig 4B, and a discretized surface of probability that prevalence exceeds 60% is depicted in Fig 4C, classed into *P < 50%*, *50% ≤ P < 75%* and *P ≥ 75%*.

A surface of the probability of endemicity class assignment (*P*_*class*_) was also calculated for each pixel (Fig 4D). Each posterior predictive realisation was assigned to an endemicity class based on the cut-off values detailed in the legend of Fig 3B. The mean of the posterior of each pixel was used to assign endemicity class. The probability of that assignment is then the proportion of posterior realisations which were in the same endemicity class. The number of endemicity classes was reduced to four with cut-off values set as follows: hypoendemic onchocerciasis: <35%; mesoendemic: ≥35% and <60%; hyperendemic: ≥60% and <80%, and highly hyperendemic (or holoendemic): ≥80% microfilarial prevalence. The latter category, although not specified in [63], has been used to define more accurately the relationship between the prevalence of infection and onchocerciasis-associated morbidity, and to investigate the feasibility of reaching operational elimination thresholds with ivermectin distribution alone [18, 22]. With four endemicity classes the minimum probability of endemicity class membership is 25%.

### Uncertainty

**Fig 5:**
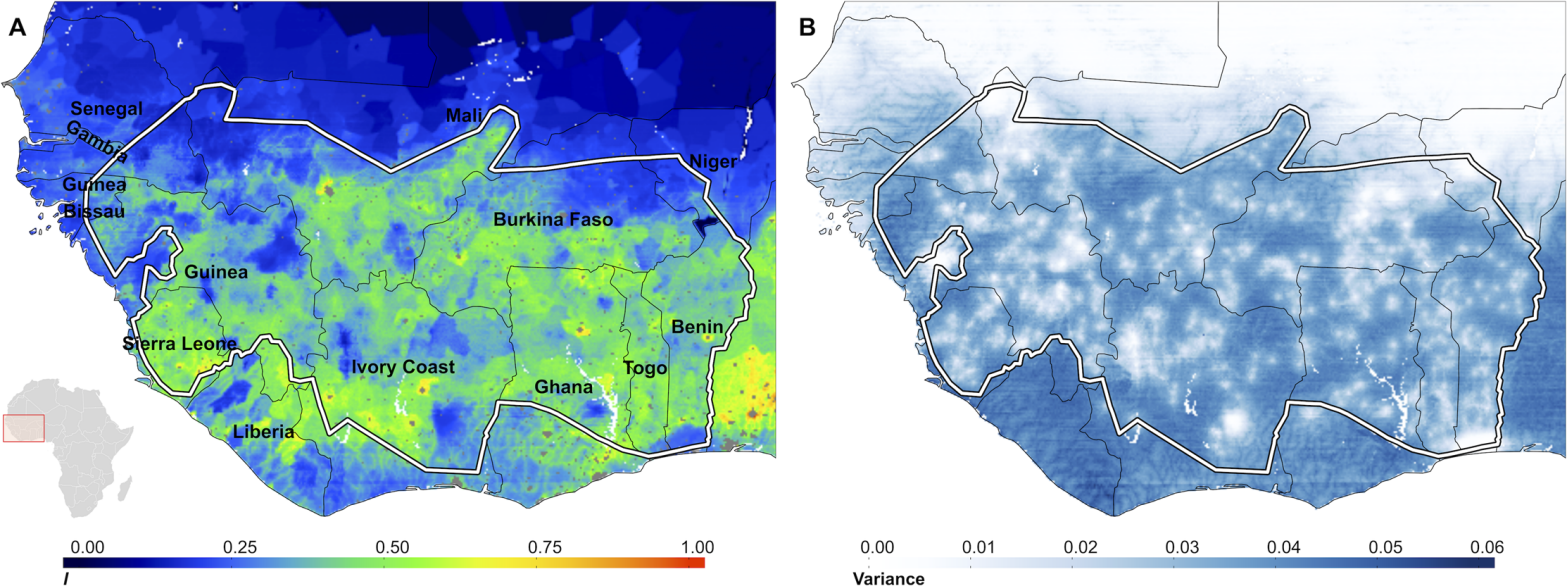
Maps of uncertainty in predictive posterior realisations. A) The population weighted index of uncertainty is calculated as in [61] and is a pragmatic representation of how important uncertainty in the predictive posteriors is likely to be. The uncertainty index is calculated by taking the log_10_(*pop*_75_ +1)×1/*P*_*class*_ where *pop*_75_ is the population count for 1975 and *Ρ*_*class*_ is the probability of endemicity class assignment (Fig 4D). B) Map of the variance of predictive posteriors. Higher values mean more diffuse posterior distributions and hence greater uncertainty in model estimates. The lowest uncertainty is found in pixels proximal to data locations. In both panels the red boundary denotes the OCP limits.

A key advantage of the MBG approach is that it permits robust evaluation of the uncertainty associated with model outputs [58]. Uncertainty may be directly inferred from the posterior distribution of the predictions, with greater uncertainty indicated by a larger variance of the posterior distribution at prediction locations. The standard deviation of the posterior predictive distribution and the population layer were used to calculate a population-weighted index of uncertainty (Fig 5A) following [64]. Maps displaying the variance of the posterior predictive distribution at each map pixel were also calculated (Fig 5B) as a visual representation of the uncertainty surrounding the prediction made at each target location.

### Model Validation

The ability of the final B-MBG GLSM model to estimate the prevalence of *O*. *volvulus* microfilarial infection prior to the inception of the OCP control interventions in the area covered by the programme was assessed, for comparability with published work, using validation criteria which have been applied in other mapping exercises using Bayesian inference to generate predictive distributions. [e.g. 64, 65]. A *K*-folds cross-validation exercise [66] was performed as previously described. In each cross-validation (CV) run, a single subsample was retained to use as the validation dataset whilst the other *K* − 1 datasets were used for training the model. For each CV, posterior predictive distributions of microfilarial prevalence were generated at unobserved validation locations for comparison against the known values. Using *K*-folds validation, each datum was used once in the cross validation. A number of validation indicators were then calculated according to the methodology used by the Malaria Atlas Project (MAP; http://www.map.ox.ac.uk/).

**Fig 6:**
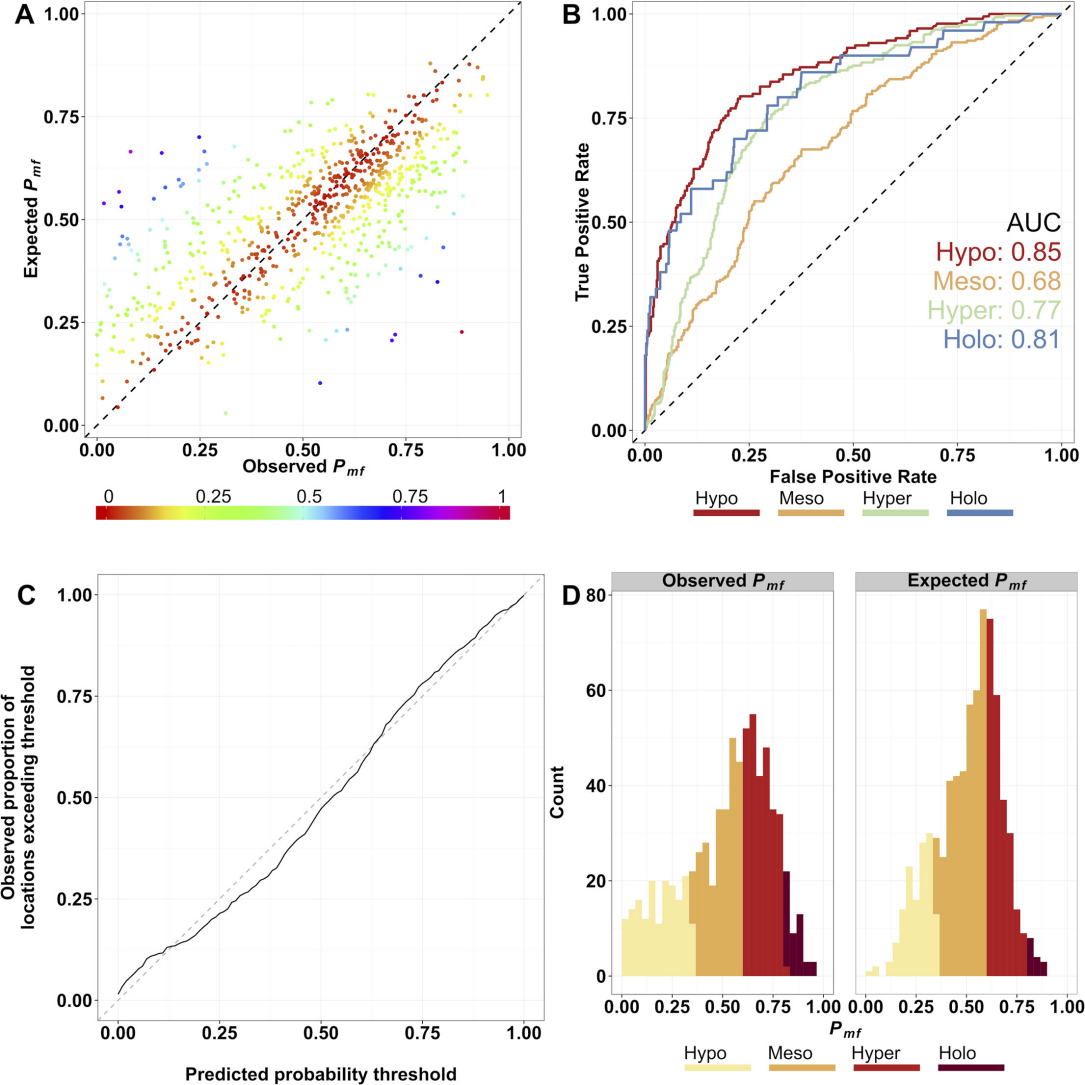
Plots of model performance. A) Scatter-plot of observed versus estimated microfilarial prevalence in those aged ≥ 5 years (*P*_*mf*_) at validation locations. The Pearson’s correlation coefficient between observed and estimated values was 0.693. The 1:1 line of perfect correlation is shown for reference. The data are coloured by the absolute difference between observed and expected values, mapped from a continuum between 0 and 1. B) Receiver Operating Characteristic (ROC) curves for each endemicity class (black line, hypoendemic; red line, mesoendemic; green line, hyperendemic; blue line, holoendemic). AUC values for each endemicity class are also displayed on the plot. C) Probability-probability plot of the proportion of true values of *P*_*mf*_ at validation locations that exceed their predicted probability threshold for a series of probability thresholds from 1% to 100%. Deviation from the 1:1 dashed line in this plot represents the difference between the distributions of observed and estimated microfilarial prevalence values at validation locations. D) Frequency histogram of observed and estimated *P*_*mf*_ at validation locations, classified according to their true class (Observed *P*_*mf*_ panel) and estimated endemicity class (Expected *P*_*mf*_ panel).

As a measure of the accuracy of point estimations the following statistics were calculated: (a) the overall Pearson-product-moment correlation coefficient (*r*) between observed and estimated values at validation locations (Fig 6A); (b) the mean prediction error; (c) the mean absolute prediction error, and (d) the ability of the model to allocate endemicity class correctly. The correlation coefficient provides a measure of linear association between observations and estimations. The mean prediction error is a measure of overall bias, i.e. the tendency of the model to systematically over- (if the value is positive) or under- (if the value is negative) estimate the true value. The mean absolute prediction error is a measure of overall accuracy, i.e. the magnitude of the difference between estimated and observed values [67]. We computed receiver operating characteristic (ROC) curves [64] and calculated the area under curve (AUC) statistic for each endemicity class to test congruency between observed endemicity classes and model-estimated endemicity classes (Fig 6B). Following [64] an AUC greater than 0.7 indicates good to excellent ability to discriminate correctly between points within and without an endemicity class.

Following [68], we tested how closely posterior distributions matched their assumed error distribution by means of a probability–probability (*P-P*) plot, which computes the expected value of microfilarial prevalence at each location, for a series of increasing probability thresholds (a total of 101 thresholds so that the range 0–100% can be explored), and compares them to the observed prevalence values (Fig 6C). Assuming errors are normally distributed, at a given probability threshold, the proportion of predicted values exceeding their observed value across all validation locations (the so-called ‘coverage probability’) should be equal to that probability threshold and therefore the *P-P* plot should show values lying perfectly along the 1:1 line. Deviations from this line indicate a tendency of the model to over- or under-represent uncertainty in point predictions. We also compared the distribution of estimated and observed prevalences at validation locations using a histogram (Fig 6D).

### Estimating Human Population Counts, Numbers at Risk, and Numbers Infected with *O*. *volvulus* in 1975

In order to estimate numbers of persons infected, maps estimating mean endemicity were overlaid on a population count layer to calculate the number of persons expected to have been infected with *O*. *volvulus* microfilariae. The number of persons at risk was assumed to be the total population aged ≥5 years living within rural areas as onchocerciasis transmission requires clean, unpolluted and fast flowing bodies of water as breeding sites for the vectors. (The selection of the ≥5-year olds was because this is the age group examined for skin microfilariae in most surveys. Children younger than 5 years of age are at risk of acquiring onchocerciasis but there was a need to make the population groups comparable to those for which infection with *O*. *volvulus* is routinely reported.)

**Fig 7:**
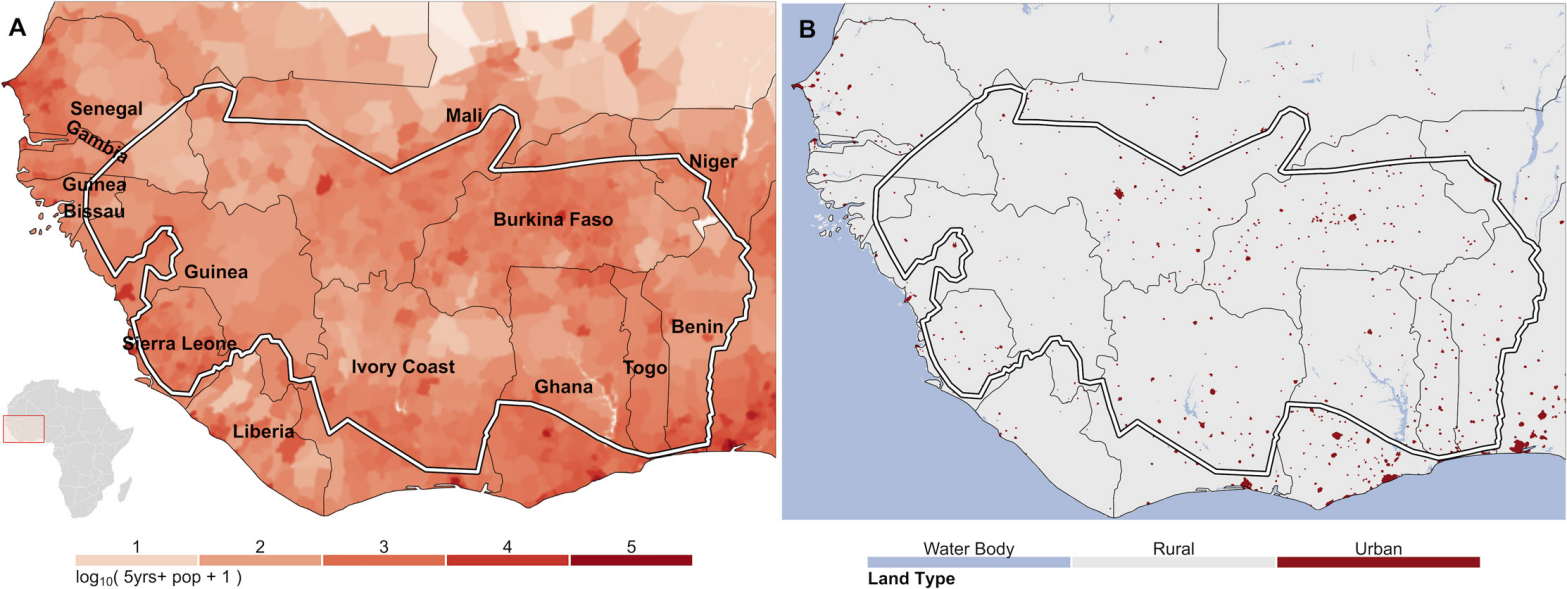
Estimated gridded population counts for 1975 and urban extents mask. A) Each map pixel contains the log_10_ transformed count of the total population +1 (to avoid negative values for population counts), aged ≥ 5 years estimated to live in that map pixel in 1975. Summing pixels by country, the totals show < 0.8% difference to the United Nations World Population Prospects (2012 Revision) country census figures for 1975. B) Red pixels delineate pixels that are classified as urban extents according the Global Rural-Urban Mapping Project (GRUMP V1.0). Estimates in urban pixels and water bodies do not count towards the population totals of numbers infected shown in Table 4. In both panels the white boundary denotes the OCP limits.

Raster global population counts are readily available from the Gridded Population of the World (GPW) v.3 data (http://sedac.ciesin.columbia.edu/data/collection/gpw-v3) from the Center for International Earth Science Information Network (CIESIN) within the Earth Institute at Columbia University [69]. These data were downloaded for 1990 (the earliest available data) and projected backwards to 1975, on a per-pixel basis, using the relevant national medium-variant, inter-censal country population growth rates from the United Nations (UN), World Population Prospects, 2012 Revision (WPP2012) database [70]. As microfilarial prevalence is often expressed in terms of the population aged 5 years and older, the proportion of the population aged 0–4 years in 1975, according to the WPP2012 database, was removed (see above). The resulting raster was then re-projected and cropped to match the study area output maps (Fig 7A). Total population counts in the estimated 1975 map were very close to the census estimates from the WPP2012 database (Table 4). The area was delineated into rural and urban areas using the gridded rural-urban extents map (GRUMP v.1) [71]. Pixels falling in urban extents were considered not-at-risk and were excluded from calculations of at-risk and infected totals, as these areas are not suitable for simuliid breeding (Fig 7B). We considered estimates of infection for the total study area (as shown by the extent of the maps), and within the OCP control area (delineated by the boundary marked in Fig 1A).

**Table 4:** Total population, at-risk population, numbers with onchocerciasis and mean microfilarial prevalence by OCP country in 1975, at the commencement of the programme.

Country	1975 total population count (this study)	1975 Total UN WPP2012 population count	At risk population*	Total No. infectedǂ	Mean prevalence^++^
Benin	3,061,326	3,262,959	1,591,242	621,600	51.8%
Burkina Faso	6,269,113	6,154,548	3,710,859	1,382,943	40.7%
Ghana^+++^	10,023,764	9,831,636	2,140,328	1,103,260	52.7%
Guinea	4,386,421	4,350,256	1,676,141	694,441	43.9%
Guinea Bissau	679,563	778,096	106,654	35,042	34.1%
Côte d’Ivoire	6,656,868	6,606,395	2,616,591	1,242,853	52.8%
Mali	6,836,293	6,191,134	3,024,280	1,108,550	37.0%
Niger	5,037,758	5,070,583	227,909	46,299	26.3%
Senegal	4,645,508	4,901,053	122,813	41,388	35.2%
Sierra Leone	2,729,938	2,820,782	1,392,505	708,285	51.3%
Togo	2,149,634	2,410,446	1,161,603	564,466	52.6%
**Total**	**52,476,186**	**52,377,888**	**17,770,925**	**7,549,127**	**45.2%**

* At risk population is defined as the total population aged 5 years and above living in rural areas within the Onchocerciasis Control Programme borders. Rural areas are defined by the Urban Extents mask (Fig 7B).

ǂ The product of the mean per-pixel microfilarial prevalence and the corresponding pixel at risk-population count. Only pixels within OCP borders count towards this total.

++ The average mean prevalence values by country, in percent. Country mean prevalence is the average of the per-pixel mean prevalence from the map output depicted in Fig 3A, within OCP borders (not the ratio between the numbers of infected and the numbers at risk).

+++ The values shown here do not include a large area in southern Ghana later incorporated into the OCP and that mainly comprised forest-type onchocerciasis.

## Results

### Model Parameters

As may be expected from the ecology of onchocerciasis, the log_10_-transformed distance to the closest river was a negative and statistically significant environmental predictor of onchocerciasis microfilarial prevalence (Table 3), confirming that as distance to rivers increases, microfilarial prevalence decreases. In addition to this, the only other (negatively associated) environmental covariates, which were statistically significant at the 95% highest posterior density (HPD; the shortest interval which contains 95% of the posterior probability), were: a) the second order polynomial term of log-transformed distance to river; b) the second order polynomial term of mean temperature of the wettest quarter (BIO8); c) precipitation seasonality (BIO15); and d) NDVI for the December–February ecological quarter. The spatial terms for the range (*ϕ*) and for the sill or variance of the spatial process (*σ*^2^) were positive and significant predictors. At the 75% HPD (the interval which contains 75% of the posterior probability), in addition to the variables mentioned above, the intercept, the mean temperature annual range, and the NDVI of the second (Mar–May) and third (Jun–Aug) ecological quarters were positively and statistically significantly associated with microfilarial prevalence, whilst the second order polynomial terms of the temperature annual range and of the precipitation seasonality were negative and significant predictors.

### Model Output: Map of Baseline Microfilarial Prevalence prior to OCP Interventions

The mean of the predictive posterior microfilarial prevalence in the population aged 5 years and older is shown in Fig 3A. The mean *O*. *volvulus* prevalence in pixels within the boundaries of the OCP area was 45.2%, ranging between 2.3% and 90.0%. The mean prevalence in map pixels outside the OCP control programme was 18.2%, ranging between 0.5% and 70.1%. It should be noted that we do not have ground-truth data points in areas outside the OCP boundary and, therefore, estimates made for such areas are more speculative and rely on data from within the OCP boundaries and their relationship with environmental covariates.

A surface of mean prevalence discretized into the endemicity levels defined in the Methods (see ‘Onchocerciasis Prevalence Data‘ section above) is shown in Fig 3B. The map of input data locations is displayed in Fig 1C.

### Map Accuracy and Predictive Probability Maps

The predictive posterior distributions are an inherent realisation of the statistical uncertainty surrounding the mean estimates. We translated the posterior distributions into operationally useful maps, depicting areas where the prevalence is below or above a certain threshold. The probabilities that microfilarial prevalence is below 35% (where disease severity is low), or exceeds 60% (where disease severity is high) are given in Fig 4A and 4B, respectively. In both cases, the greatest levels of uncertainty were associated with areas where the posterior predictive mean of prevalence fell in the mesoendemic endemicity class, and in areas where we do not have ground-truth data points to inform the model, such as areas outside the OCP boundaries. Where predictions were either in hypo- or holoendemic classes, the probability of threshold exceedance was either very low or very high, respectively. The effect of the environmental covariate, distance to river, is visible in these continuous probability threshold maps. The outline of the stream network can be seen in Fig 4A as lighter shade in dark areas indicating a low probability that microfilarial prevalence is < 35% along the stream network. In Fig 4B the river network is also visible as darker colours in light areas, indicating a higher probability that microfilarial prevalence exceeds 60% close to the river. Fig 4C describes the probability that prevalence exceeds the clinically important threshold of 60% microfilarial prevalence, indicating areas with 0 to 50%, 50 to 75%, and 75 to 100% probability that areas are at least hyperendemic. There is a band of high-probability of exceedance of the hyper-endemic threshold across the core area of the OCP, from Guinea, through Côte d’Ivoire, Ghana and Southern Burkina Faso, to Togo and into Benin. This band also extends into Nigeria, despite there being no input data points there; a country which was later included in the mass treatment programmes under APOC (see Fig 2 and Fig 3 of [37] by way of validation of our results). The average probability of endemicity class membership across all map pixels was 66% (Fig 4D), with a range of 34% to 100%. This means that on average, 66% of the posterior realisations for any given map pixel were within the same endemicity class. The most uncertain endemicity class membership for individual map pixels appears to be in the core areas of the OCP, predominantly in meso- and hyper-endemic classified pixels. However, examination of the signal to noise ratio (SNR) map (see S1 File Text H ‘Further maps derived from the predictive posterior distributions’) indicates that these are the areas with the highest SNR values, as may be expected due to their proximity to ground-truth data points (Fig L of S1 File). This suggests that the prevalence cut-off values used to define endemicity classes are less well-suited to this study. Pixels with estimates of prevalence close to endemicity class boundaries will be less easily classified to a given endemicity class, and instead a more finely discretized map, such as the map in Fig 3A, would be more informative.

We modelled the probable importance of the uncertainty surrounding endemicity class membership as in [64], using a population-weighted index of uncertainty. Briefly, in areas where the probability of endemicity class membership is not well defined (a low probability within the overall range of 0.25–1 in this study), and the population density is also high, the possible marginal effect of that uncertainty in the predictive posterior distribution of microfilarial prevalence will be high. The population-weighted uncertainty map depicted in Fig 5A, which has an uncertainty index value scaled between 0 and 1, is highly overdispersed, with > 90% of the pixels in the map having an uncertainty index of < 0.5 assigned to them.

### Model Validation

The overall correlation coefficient, *r*, between observed and estimated values at validation locations was 0.693 (Fig 6A). Calculating separately a correlation coefficient, *ρ*, for each of the 10 *K*-folds individually, we obtained a range of values varying between 0.566 and 0.806 with a median of 0.699. The mean prediction error was 0.77% (i.e., overall, observed prevalence was greater than the estimated prevalence by less than 1%). The mean absolute error indicated that, on average, there was a difference of 12.3% between the observed and estimated microfilarial prevalence at validation locations. The variograms depicted in Fig 2 show that there was significant spatial dependence in the data (Fig 2A), even after accounting for the effect of environmental covariates (Fig 2B), as with increasing separation distance there was increasing semivariance that lies outside the envelope of semivariance values expected by chance (the grey shaded area—a Monte Carlo envelope calculated from random reassignment of prevalence values to different data locations). The expected onchocerciasis endemicity class exactly matched the observed endemicity class for 406/737 (52.7%) validation locations and was within one endemicity class at 711/737 (96.5%) locations, showing good agreement between observed and estimated classes. Serious misclassification of endemicity class (i.e. classifying a point to a non-adjacent class) occurred in 26/737 (3.5%) cases. A contingency table by endemicity class is given in Table 5. AUC statistics for each endemicity class were calculated using ROC curves (Fig 6B), and with the exception of the mesoendemic class, all other endemicity classes had good to very good agreement between observed and predicted endemicity classes with AUC values of 0.85 for hypoendemic; 0.77 for hyperendemic, and 0.81 for holoendemic classes. The mesoendemic class fell just slightly below the threshold for good predictive capacity, with a value of 0.68.

**Table 5:** Classification of survey villages according to observed and expected (model-derived) endemicity class for all validation locations.

Expected Class	Observed Class				Total
	Hypoendemic	Mesoendemic	Hyperendemic	Holoendemic	
Hypoendemic	91	32	9	1	133
Mesoendemic	74	172	123	9	378
Hyperendemic	7	45	134	29	215
Holoendemic	0	0	2	9	11
**Total**	172	249	268	48	737

Following [64, 65], we also tested how closely posterior distributions matched their assumed normal error distribution by means of a *P-P* plot (Fig 6C). The line is close to the 1:1 line of a perfect error model, but falls below the 1:1 line for values between probability thresholds of 0.15 to 0.60, meaning that there is a slight bias of the model to overestimate the probability of taking values closer to the middle of the range of possible prevalence values, whilst slightly under-representing extreme (very low or very high) microfilarial prevalence values.

This phenomenon can be seen on the histogram in Fig 6D. The histogram of prevalence at input data locations is negatively skewed, whilst the histogram of estimated prevalence at validation locations exhibits a smaller degree of skewness, with a greater proportion of estimated values falling in the meso- and hyper-endemic categories.

### Estimated Numbers of People Living in the OCP Area, Infected with *O*. *volvulus* and At-Risk of Onchocerciasis in 1975

The population totals calculated in this study are very close to the country-level census numbers provided in [70]. The total gridded population living in OCP countries in 1975 according to our estimates was 52.5 million, comparing well with the total UN estimate for OCP countries of 52.4 million persons. We combined the map of mean microfilarial prevalence in those aged 5 years and above with a map of the estimated corresponding population for 1975 to derive estimates of numbers of people infected with *O*. *volvulus*. We estimated that there were 7.55 million persons with onchocerciasis at the outset of the OCP living within the control programme’s boundaries (approximately 1.3 million km^2^ [9]). The total number of persons estimated to be at risk of onchocerciasis within the OCP borders was estimated to be 17.8 million. Within the study area, there were an additional 11.6 million persons aged 5 years old or more, living in rural areas in OCP countries, but outside of the control programme’s borders. From the model estimates, we calculated that an additional 2.8 million of these persons may have been living with onchocerciasis in these areas. Of these, 1.1 million persons were in Ghana, where the control programme subsequently extended southwards from the original OCP boundaries to cover forest areas known to be endemic but not previously covered under the OCP. The total numbers of persons at-risk and infected, by country, are shown in Table 4.

## Discussion

To the best of our knowledge, we have presented the first continuous map of the pre-control prevalence of *O*. *volvulus* microfilariae in West Africa, in the region previously covered by the OCP and, in addition, have identified environmental covariates that aid in the estimation of onchocerciasis prevalence in un-sampled areas. For instance, our estimated pre-control infection prevalence distribution in the totality (Liberia) or part (Nigeria) of two non-OCP countries fares well with that presented, respectively, in [72] and [37]. We have also provided comprehensive estimates of the initial numbers of persons infected and at risk of infection, and consider a robust handling of the uncertainty surrounding those estimates. According to our model, the mean baseline infection prevalence in the rural areas of the OCP was 45%. By comparison, Zouré et al. [37] estimated that in APOC areas and for 2011―had control interventions not been implemented―the average microfilarial prevalence of infection in the entire rural population would have been 15%. This estimate, based on a conversion of nodule prevalence into microfilarial prevalence using the relationship described in Coffeng et al. [73], is much lower than ours. APOC covers a much larger and more environmentally and geopolitically heterogeneous area, which may contribute to explain some of the difference in infection prevalence estimated in the two studies.

Although levels of infection have decreased as a result of the interventions implemented by the OCP [8–12], and environmental change has occurred in the intervening years since 1975 [74], a map of the baseline levels of prevalence and endemicity provides an indicator of initial conditions for transmission, which are influential determinants of the feasibility of onchocerciasis elimination with ivermectin [17, 18, 22]. This is particularly relevant, since after cessation of OCP vector control operations in 2002, and subsequently in special intervention zones [13], blackfly vector populations have recuperated to original levels in many areas and there is evidence of continuing transmission within the former OCP area [75–77].

### Representativeness of the Data

The OCP epidemiological dataset represents the most complete source of standardised parasitological survey data for onchocerciasis in West Africa. However, although extensive surveys were conducted (see Fig A in S1 File for a map of all locations surveyed during the OCP), only a small fraction of the total population was sampled (the subset of the OCP dataset which was selected as the most representative of pre-control prevalence consisted of approximately 145,000 persons skin snipped). One of the problems faced when using the OCP data for mapping purposes, was that the population and epidemiological surveys were not performed at random, introducing some bias in the data towards areas of higher endemicity. Original programme areas were delineated, for the purposes of enabling evaluation of the impact of vector control, using cartographic and entomological knowledge about the location of rivers, flow speeds and vector breeding sites. In a limited number of epidemiological settings more detailed studies were carried out with the objective to map the distribution of onchocerciasis, and villages were classified as first-line if there were no other settlements between themselves and vectors’ breeding sites [78, 79]. Further surveys were conducted in a selection of villages at regular intervals up- and down-stream from the breeding site, and a random sample taken from second- and third-line villages at increasing distances from the river [79]. Including this information in the model when estimating the effect of environmental covariates may have helped increase predictive accuracy, but it would be difficult to classify map pixels into first-line or otherwise without complete knowledge of the distribution of all villages across the study area. It was not necessary at the time to conduct a complete census of the resident population (although a census was carried out in each survey village) in the OCP area and its extensions, because control initially relied on vector control (and not on mass distribution of ivermectin, for which censuses are mandatory).

### Use of Environmental Covariates and a Spatial Trend

The use of environmental covariates to improve model accuracy was investigated and a number of variables associated, or deemed to be potentially associated, with onchocerciasis was investigated. A model incorporating covariates was found to be a better predictor of onchocerciasis prevalence and endemicity than an intercept-only model, which incorporated only the spatial trend. Inclusion of covariates improved (reduced) the DIC values in model validation and improved the correlation between observed and predicted prevalence values at validation locations. In addition to the environmental covariates listed in Table 3, we also tested for any effects of spatial differences between the geographical distributions of different members of the *S*. *damnosum* complex but we could not detect any significant influence of vector taxon on the model’s predictions. (Had we modelled infection intensity rather than prevalence, an effect of vector taxon might have become apparent.) However, the value of *ϕ* ≈ 38 corresponds to a spatial trend that reaches 95% of its maximum semi-variance over a distance of approximately 113km (i.e., for any prediction location, its estimated value is influenced by locations up to 113km apart). The range parameter is consistent with the observation that blackfly vectors can travel several hundred kilometres from a breeding site [80]. However, Renz et al. (1987) found biting density to drop to 10% of its original level at distances of 5km from riverine breeding sites in Sudan-savanna focus of Northern Cameroon [81]. Other studies have shown that people may be bitten by female blackflies in locations distant from any known breeding site [82–84] and that re-invasions of parous flies into areas previously cleared through larviciding can happen from breeding sites as far as 300km away [85]. This lends strong epidemiological and ecological support to the operation of a spatial trend operating over this distance.

We extended the methods of Slater et al. [36] to carry out a robust, Bayesian step-wise selection procedure to identify the best set of uncorrelated explanatory covariates. The chosen covariates significantly improved the ability of the model to explain the variation in recorded microfilarial prevalence. The covariates also helped to define natural geographical limits to the mapping which would otherwise have been impossible, given the bias towards surveyed villages with a high probability of having onchocerciasis. In other mapping studies, pseudo-absence points have been introduced into the data in un-sampled areas which are suspected to be non-endemic, to help delineate risk-free areas. In this study we have avoided this, and instead allowed the limits to be defined by the environmental predictors and the spatial covariance function in the model. We found that the model estimated very low prevalence in the very dry arid areas north of the OCP boundaries, without recourse to including pseudo-absence points.

There is a zone of southeastern Côte d’Ivoire in Figs 3 and 4 where the model predicts non-endemicity and hypoendemicity with patches of mesoendemicity, although it captures a hyperendemic hotspot around the lower Bandama river. The prediction for an area east of this location is consistent with a zone where there are very few *S*. *damnosum* s.l. breeding sites in the middle section of the River Comoe and its surrounds (see Figures 2 and 3 of [42]). However, for the lower Bandama river and the lower Comoe river, a previous study by Dadzie et al. (1990) [86] found a high prevalence of onchocerciasis in 11 first-line villages in southern Côte d'Ivoire which, with one exception, were located south of the OCP boundary, but they did remark that the CMFL values were “relatively low”. Similarly, hyperendemicity in the Cavally river valley in western Côte d’Ivoire [87], the map for which was erroneously transposed with the one in [88] was missed by the model. Whilst our map estimates show elevated prevalence along stream lines in the southeastern Côte d’Ivoire area, the prevalence is not as high as that found in the Dadzie and co-workers’ study. The villages in that study were all situated ≤ 1km from known breeding sites in rivers, making this sample not wholly representative of the epidemiological situation in southern Côte d'Ivoire. The villages in our study are located between 0 and 26km from the nearest river (mean = 5.15km, median = 3.86km), and because our pixel resolution is of 5km x 5km, our model may have smoothed over some of the highly localised nature of the elevated endemicity reported in [86]. In addition, the model relied heavily on only two village data sets in this area with a considerable distance between them on a west to east axis, both of which were points of non-endemicity (Fig 1C). Nevertheless, that these points reflected the true situation in the zone between the Bandama and Comoe rivers is supported by epidemiological data collected in an area due north of Abidjan and due east of Yamoussoukro in October 2014 that revealed only hypoendemic villages. An additional potential explanation is that we may be missing important environmental covariates that would be able to explain the high endemicity in this particular area. In the southern extremes of the study area the land type is dense forest, and forest-type onchocerciasis is known to produce high prevalence; we may be missing some important covariates associated with this bioclime, as most of the area for which the model produces estimates of initial onchocerciasis prevalence belongs to the savannah bioclimes. Finally, in areas with ground truth data, the variable distance to river is helpful for modelling the distribution of endemicity around rivers, but in those without ground truth data, the model may not be able to predict high endemicity levels on the basis of the environmental variables only. Some of these variables are probably not capturing determinants of high endemicity along river courses, such as the locations and productivity of vector-breeding grounds. This may require other environmental information such as that used in the remote sensing model presented in [89].

We confirmed that microfilarial prevalence is statistically significantly and negatively associated with increasing distance from rivers in both the linear term (*p*-value < 0.01) and the quadratic term (*p*-value < 0.05). Distance between village and river is an important determinant of vector biting rate [81] and onchocerciasis endemicity when the river contains rapids and other suitable and identifiable vector breeding sites, according to a classification of first, second and third line villages [90, 91].

Mean temperature of the wettest quarter (BIO8) was also significantly and negatively correlated with prevalence (*p*-value < 0.05). Interpretation of this result is rather more difficult because the wettest quarter is necessarily different across different map pixels, but it suggests that conditions for blackfly breeding and transmission may be more favourable in areas in which the majority of precipitation occurs in the cooler parts of the year. This is linked with precipitation seasonality (BIO15), which was also significantly and negatively correlated with infection prevalence. Areas with greater intra-annual variability in rainfall (and therefore possibly representative of more seasonal transmission) were negatively associated with prevalence (the coefficient of variation of precipitation seasonality is the standard deviation of the weekly precipitation estimates expressed as a percentage of the mean of those estimates). Areas with a more constant weekly rate of precipitation (perhaps indicative of perennial transmission), which also occurs during the cooler parts of the year (possibly favouring vector survival), are positively associated with microfilarial prevalence. This is compounded by the negative parameter value for temperature annual range (BIO7). A smaller difference between the highest weekly maximum temperature and the lowest weekly maximum temperature had a positive effect on prevalence (presumably through an effect on developmental rates of blackfly immature stages, and perhaps also of the parasite larval stages within the vectors), which are highly dependent on temperature [92].

The normalized difference vegetation index (NDVI) for the December–February ecological quarter was a statistically significant and negative predictor of microfilarial prevalence (*p*-value < 0.05). This quarter follows a period of low precipitation within the OCP area during which the temperature is still relatively mild and the highest NDVI values for this quarter are found only along the coastal regions, which are unsuitable as blackfly breeding habitats. When relaxing the BCIs to 75% (i.e. at *p*-value < 0.25), NDVI for other ecological quarters became significantly and positively associated with prevalence. In particular, areas with more lush green vegetation during March–May and June–August were positively associated with onchocerciasis prevalence. These greener areas occur within the OCP boundaries, whereas the desert areas situated north of the programme boundaries are extremely arid due to lower rainfall and higher temperatures, with very low NDVI values and negatively associated with the presence of onchocerciasis. Compound topographic index (a proxy for the slope and relative wetness of the land) had a weak effect in the model, as did the precipitation of the wettest quarter (BIO16).

Altitude has been found to be positively correlated with onchocerciasis prevalence in various foci of Cameroon [93], Venezuela [94], and Guatemala [95] up to an altitude of 1,500 metres above sea level, and negatively correlated above that altitude, with most of the proportional increase taking place at altitudes up to 500 m [93–95]. However, this association may also depend on the type of river and its geomorphological substrate, determining vector breeding suitability (and in Venezuela also an association with vector species composition and the relative vector competence of the various simuliid species for *O*. *volvulus*), and not merely on altitude [96, 97]. In this study, altitude was not found to be a significant predictor of onchocerciasis; however, this could be partly explained because both rainfall and temperature variables were included in the model, which may remove some of the effect of altitude on prevalence, and the fact that > 94% of the total land area in the OCP is < 500 metres elevation.

### Model Implementation and Validation

In line with other recent approaches to large-scale geostatistical mapping [28–36], we used MCMC methods to fit a Bayesian statistical model, which uses linear combinations of the survey data (microfilarial prevalence) and a spatial covariance function to obtain predictive posterior estimates at un-sampled locations. The distribution of such estimates readily translates into measures of uncertainty associated with microfilarial prevalence. For those non-surveyed locations, which are distant from those with data, the posterior distribution tends to be more diffuse, indicating greater uncertainty.

In this study we used 25km^2^ map pixels. This was chosen as a compromise between achieving a desirable degree of spatial resolution and minimising computing time, but led to some loss of short-scale variation in prevalence below the chosen value. This size of individual map pixels means that very short-scale variation between closely situated villages is smoothed over and is likely to be important given the exponentially decaying relationship between distance from the river and biting density/proportion of parous flies that occurs within this distance [81]. A consequence of this smoothing effect is that although the predicted surface provides a robust prediction of endemicity at larger scales, it is less capable of representing variations over very short distances, and the estimation of extreme values may be truncated. The output range of prevalence values in those aged 5 years and over in the predictive map was slightly narrower (from 0.5% to 90%) than the input data (from 0% to 95%), a feature of spatial smoothing also reported by others [64, 65, 98].

Our validation statistics compare reasonably well with those presented in other mapping exercises, such as MAP for 2007 [64] and 2010 [65], which also applied MBG procedures but (in the case of MAP 2007) did not find a strong relationship between malaria prevalence (*Plasmodium falciparum* in those aged 2–10 years) and a range of remotely-sensed environmental covariates. (In their update of global *P*. *falciparum* endemicity map for 2010, Gething et al. [65] succeeded in identifying environmental covariates as predictors of malaria prevalence.) Although we obtained a lower correlation coefficient (0.69 in our case versus 0.82, for the 2007 version of MAP in Africa [62]), our sample size was 7 times lower (737 versus 5,307 input data points). The mean prediction error (0.8% vs. 0.8%, indicating a tendency to overestimate prevalence by less than 1%), and the mean absolute errors (12% vs. 11%) are comparable. (In the 2010 MAP iteration for Africa, the correlation coefficient between predicted and observed values was 0.86; the mean prediction error was –0.9%, indicating a tendency to underestimate prevalence by less than 1%, and the mean absolute error was 12% [65].) The ability to predict endemicity class correctly was also lower (59% vs. 71% in the 2007 MAP), but there was only a value of 3.5% for serious estimation errors (i.e. non-adjacent class classification) when using four endemicity classes (the hypoendemic class was expanded to include the non-endemic class). The ability of the model to allocate locations correctly into endemicity class partly depends on the boundaries and number of categories chosen to represent endemicity levels, and therefore it is not a wholly unbiased metric of map accuracy. (With *n* endemicity classes there are (*n –* 1)^2^ – (*n –* 1) chances for misclassification—allocating to a non-adjacent endemicity class—in the *n*×*n* matrix; hence, the probability of misclassification increases non-linearly, asymptotically approaching 1, with increasing *n*).

### Population at Risk and Numbers Infected with *O*. *volvulus* in 1975

The United Nations estimated the 1975 population for the 11 countries covered by the OCP at 52.4 million [70]. Our back-calculation sets this figure at 52.5 million (only 0.2% higher). Boatin [9] stated that by the time the OCP ended in 2002, 27 years later, the estimated population in the area covered by the programme was over 78 million.

The Third Report of the Expert Committee on Onchocerciasis, published in 1987 [99], indicated that in the OCP area, the number of persons at risk (as assessed in 1984–1985) was 7.2 million, with 2.4 million infected (a ratio of 3:1). Since this assessment was made approximately 10 years after the commencement of antivectorial operations in the seven initial countries of the OCP (namely, Benin, Burkina Faso, Côte d’Ivoire, Ghana, Mali, Niger and Togo), the report excluded persons living in previously endemic areas of these countries where transmission was deemed to be controlled from the calculations of the numbers at risk. In those areas, the numbers of persons infected with *O*. *volvulus* were calculated as 25% of those who would have been infected at the beginning of the control programme [99]. Consequently, the numbers infected at the start of the OCP would have been 5.9 million in the initial 7 countries, and 6.8 million adding Guinea, Guinea Bissau, Senegal, and Sierra Leone (which were incorporated later). According to our mapping exercise, the numbers of persons at risk would have been 2.5 times as large as the number reported in 1987, at 17.8 million (representing the total population aged 5 years and above living in rural areas within the OCP borders). The number of people with onchocerciasis would have been 7.55 million, i.e. three times as large as the number documented in 1987 (which gives directional support to the report’s consideration that the figures given would have been 25% of those prior to control). The report also acknowledges that the figures for the prevalence of infection most likely underestimated the true extent of the problem, as they were obtained by extrapolation from relatively small sample surveys, without accurate knowledge of the local distribution of endemic foci (and certainly without considering spatial correlation between survey points). Survey villages were selected from historical records, for their hyperendemicity of onchocerciasis, and other criteria aimed to ensure long-term evaluation of vector control. Village selection was somewhat independent of the entomological evaluation network, and consequently, although successful in evaluating the vector control campaign, it could not have described the distribution and severity of the disease adequately and did not allow for correct estimates to be made of the number of people infected with *O*. *volvulus* [100].

A study of the distribution of onchocerciasis was conducted in 1987 in the Western Extension [100], partly conceived to overcome these limitations. After discarding population sizes greater than 800 inhabitants, the authors calculated a rural population of 4.5 million persons and reported that, of these, 1.5 million persons were infected. Using the Gridded Population of the World dataset, and satellite-derived GRUMP data as a clipping mask to remove urban extents, our model estimated that the rural population at risk within the Western Extension in 1975 would have been 4.5 million, with 1.9 million persons infected. The congruence between our study and that of [100] is excellent. Another study by De Sole et al. [101] investigated the distribution and severity of onchocerciasis in Southern Benin, Togo and Ghana. There is also excellent concordance between the hyperendemic areas delineated by the maps in our study and those areas proposed to be at elevated risk of onchocerciasis-associated blindness in the De Sole et al. [101] study. The district of Pru on the north-western fringes of Lake Volta in Ghana is particularly congruent between the two studies. Both of the De Sole et al. studies [100, 101] were carried out in proposed extension areas in order to determine the pre-control geographical distribution of *O*. *volvulus* in such areas. Where this type of surveys was carried out, accurate knowledge of the distribution of pre-control onchocerciasis prevalence was obtained, now confirmed with the modern mapping methods we have applied. However, in the original OCP areas, where the main focus was not to determine the distribution of the disease but to identify vector breeding sites to monitor vector control operations, the pre-control prevalence of onchocerciasis was greatly underestimated [99].

Finally, Coffeng et al. [26] estimated that before the inception of APOC in 1995 about 32 million people in APOC areas were infected with onchocerciasis (Zouré et al. [37] estimated a figure of 35.6 million in 2011 had interventions not been implemented). Adding these figures to our 7.55 million infected prior to the inception of the OCP, we obtained a total value of 40–43 million people infected in Africa at baseline. This total lends support to the recent estimation of 37 million people infected with *O*. *volvulus* in the continent [8, 102], and supersedes the frequently quoted, but grossly underestimated, previous figure of about 18 million infected [99].

### Directions for Future Work and Concluding Remarks

The Disease Reference Group for Helminth Infections (DRG4) of the UNICEF/UNDP/World Bank/WHO Special Programme for Research and Training in Tropical Diseases (TDR), recently identified that an essential pre-requisite for statistical and dynamical modelling to effectively aid and inform helminthiasis control programmes, is that of creating open access, high quality, epidemiology and control databases [103]. It is the aim of other infectious disease mapping projects (e.g. Global Atlas of Helminth Infections, http://www.thiswormyworld.org; Malaria Atlas Project, http://www.map.ox.ac.uk; Global Atlas of Trachoma Prevalence, http://www.trachomaatlas.org) to become an open-access information resource on the distribution of these infections, to which the community of public health workers can contribute their own data. Progress towards this goal has been made particularly for soil-transmitted helminthiases.

Among the questions for onchocerciasis that need addressing, are those concerning: a) how best to quantify the progress of the aforementioned control programmes, describing in time and space changes in infection prevalence, intensity, and morbidity after the commencement of interventions?; b) how to identify in time and space hotspots of persistent transmission and investigate the causes for such persistence (e.g. poor geographic/therapeutic coverage and compliance vs. decreased drug efficacy)?; c) how best to evaluate the feasibility of local elimination of the infection reservoir and the probability of recrudescence or reinvasion once interventions cease? and d) how is the burden of onchocercal disease changing under sustained control [26, 104]?

The DRG4 group also identified, as a modelling research priority, the need for linking MBG maps with population dynamics frameworks to both update the information on endemicity in future iterations of the maps; to compare the outputs with those, derived from transmission models, of long-term projections of the impact of interventions on infection prevalence, intensity, and associated disease [26,103], and to project in which foci the 2020 goals will be achieved with current tools and where they will not be reached within reasonable time horizons without implementing novel and/or complementary control interventions. An insightful, albeit complex, analysis might be obtained by approaching this question as a spatio-temporal problem with interventions (of at least two kinds, namely antivectorial and antiparasitic) occurring at different times in different places.

Finally, it would be possible to extend the geostatistical model developed here to map the endemicity of onchocerciasis in APOC’s countries. APOC has made remarkable progress using REMO to guide the implementation of CDTI [105], and model-based geostatistical maps (based on the spatial dependency component of the model, without inclusion of environmental covariates), have recently been published [37]. However, application of the methodology described here may make it possible to refine estimation of infection prevalence at locations for which no ground-truth data are available by using information from the identified environmental covariates (such as distance to nearest river). It would also be crucial to develop MBG tools to jointly map onchocerciasis–loiasis endemicity and co-endemicity [34, 106–108] as the risk of severe adverse effects due to ivermectin treatment in the latter [109, 110] presently hinders progress towards the control and elimination of onchocerciasis in the continent by 2020.

## Supporting Information

S1 File Detailed description of data and methods. Text A. Data description and pre-control data selection criteria. Text B. Parasitological survey methods and geographical limits of the mapping. Text C. Processing of environmental covariates. Text D. Maps of environmental covariates. Text E. *K*-folds model validation. Text F. MCMC chain diagnostics. Text G. Posterior distributions of model parameters. Text H. Further maps derived from the predictive posterior distributions. Text I. Geospatial software. Table A. Population growth rates, factor by which the 1990 population count was multiplied for back-calculation to 1975, and proportion of the population aged 0–4 years for OCP countries. Fig A. Village locations in the OCP epidemiological database. Fig B. Location of the data points selected for use in this study after applying pre-control selection criteria. Fig C. Scatterplot of age- and sex-standardised microfilarial prevalence in those aged 5 years and above (*P*_*mf*_), against the crude, unadjusted prevalence. Fig D. Heatmap of distance (in Km) to nearest pre-control survey data point for each map pixel in the study area. Fig E. Lambert Azimuthal Equal Area projection of the world, centred on Africa. Fig F.1. Maps for covariate values at prediction locations, for NDVI *β*_*j*_ covariates included in the final predictive model. Fig F.2. Maps for covariate values at prediction locations, for all other *β*_*j*_ covariates included in the final predictive model. Fig G. The location of validation points in each *K*-fold cross-validation dataset. Fig H. MCMC traceplots of all *β*_*j*_ regression coefficients. Fig I. MCMC traceplot of the value of the range parameter, *ϕ*. Fig J. Highest posterior density (HPD) plots of model parameters. Fig K. Mean of predictive posterior distributions for estimated microfilarial prevalence of *Onchocerca volvulus* prior to the commencement of the Onchocerciasis Control Programme in West Africa (OCP) in 1975. Fig L. Signal to noise ratio between mean and standard deviation of predictive posteriors. Fig M. Histogram of model output mean (left) and median (right) microfilarial prevalence values at all prediction locations.
(PDF)

## References

[1] London Declaration on Neglected Tropical Diseases. Uniting to combat neglected tropical diseases: Ending the neglect and reaching 2020 goals. 2012. [http://unitingtocombatntds.org/sites/default/files/resource_file/london_declaration_on_ntds.pdf]. Accessed 30 November 2015.

[2] World Health Organization. Accelerating work to overcome the global impact of neglected tropical diseases–A roadmap for implementation. 2012. [http://www.who.int/neglected_diseases/NTD_RoadMap_2012_Fullversion.pdf]. Accessed 30 November 2015.

[3] Kim YE, Remme JH, Steinmann P, Stolk WA, Roungou JB, Tediosi F. Control, elimination, and eradication of river blindness: scenarios, timelines, and ivermectin treatment needs in Africa. PLoS Negl Trop Dis. 2015;9(4): e0003664 (Correction in PLoS Negl Trop Dis. 2015;9(5): e0003777). 10.1371/journal.pntd.0003664 25860569 PMC4393239

[4] Bradley JE, Whitworth J, Basáñez MG. Onchocerciasis In: Wakelin D, Cox F, Despommier D, Gillespie, editors. Topley and Wilson’s Microbiology and Microbial Infections. Volume Parasitology. 10th edition London: Hodder Arnold; 2005 pp: 781–801.

[5] Murdoch ME, Hay RJ, Mackenzie CD, Williams JF, Ghalib HW, Cousens S, et al A clinical classification and grading system of the cutaneous changes in onchocerciasis. Br J Dermatol. 1993;129(3): 260–269. 8286222 10.1111/j.1365-2133.1993.tb11844.x

[6] Little MP, Breitling LP, Basáñez MG, Alley ES, Boatin BA.Association between microfilarial load and excess mortality in onchocerciasis: an epidemiological study. Lancet. 2004;363(9420): 1514–1521. 15135599 10.1016/S0140-6736(04)16151-5

[7] Walker M, Little MP, Wagner KS, Soumbey-Alley EW, Boatin BA, Basáñez MG. Density-dependent mortality of the human host in onchocerciasis: relationships between microfilarial load and excess mortality. PLoS Negl Trop Dis. 2012;6(3): e1578 10.1371/journal.pntd.0001578 22479660 PMC3313942

[8] Basáñez MG, Pion SDS, Churcher TS, Breitling LP, Little MP, Boussinesq M. River blindness: a success story under threat? PLoS Med. 2006;3(9): e371 17002504 10.1371/journal.pmed.0030371 PMC1576321

[9] Boatin B. The Onchocerciasis Control Programme in West Africa (OCP). Ann Trop Med Parasitol. 2008;102 (Suppl 1): 13–17. 10.1179/136485908X337427 18718148

[10] Hougard JM, Alley ES, Yaméogo L, Dadzie KY, Boatin BA.Eliminating onchocerciasis after 14 years of vector control: a proved strategy. J Infect Dis. 2001;184(4): 497–503. 11471108 10.1086/322789

[11] Boatin BA.The current state of the Onchocerciasis Control Programme in West Africa. Trop Doct. 2003;33(4): 209–214. 14620423 10.1177/004947550303300407

[12] Hougard JM, Yaméogo L, Sékétéli A, Boatin B, Dadzie KY. Twenty-two years of blackfly control in the onchocerciasis control programme in West Africa. Parasitol Today. 1997;13(11): 425–431. 15275144 10.1016/s0169-4758(97)01145-9

[13] World Health Organization/African Programme for Onchocerciasis Control. Onchocerciasis control in special intervention zones including Sierra Leone in the OCP area. Joint Programme Committee Report, September 2002. Ouagadougou (Burkina Faso): World Health Organization/African Programme for Onchocerciasis Control; 2002.

[14] Diawara L, Traoré MO, Badji A, Bissan Y, Doumbia K, Goita SF, et al Feasibility of onchocerciasis elimination with ivermectin treatment in endemic foci in Africa: first evidence from studies in Mali and Senegal. PLoS Negl Trop Dis. 2009;3(7): e497 10.1371/journal.pntd.0000497 19621091 PMC2710500

[15] Traore MO, Sarr MD, Badji A, Bissan Y, Diawara L, Doumbia K, et al Proof-of-principle of onchocerciasis elimination with ivermectin treatment in endemic foci in Africa: final results of a study in Mali and Senegal. PLoS Negl Trop Dis. 2012;6(9): e1825 10.1371/journal.pntd.0001825 23029586 PMC3441490

[16] Tekle A, Elhassan E, Isiyaku S, Amazigo U, Bush S, Noma M, et al Impact of long-term treatment of onchocerciasis with ivermectin in Kaduna State, Nigeria: first evidence of the potential for elimination in the operational area of the African Programme for Onchocerciasis Control. Parasit Vectors. 2012;5(1): 28. 22313631 10.1186/1756-3305-5-28 PMC3296569

[17] Winnen M, Plaisier AP, Alley ES, Nagelkerke NJD, van Oortmarssen G, Boatin BA, et al Can ivermectin mass treatments eliminate onchocerciasis in Africa? Bull World Health Organ. 2002;80(5): 384–390. 12077614 PMC2567795

[18] Turner HC, Churcher TS, Walker M, Osei-Atweneboana MY, Prichard RK, Basáñez MG. Uncertainty surrounding projections of the long-term impact of ivermectin treatment for human onchocerciasis. PLoS Negl Trop Dis. 2013;7(4): e2169 10.1371/journal.pntd.0002169 23634234 PMC3636241

[19] Remme J, Ba O, Dadzie KY, Karam M.A force-of-infection model for onchocerciasis and its applications in the epidemiological evaluation of the Onchocerciasis Control Programme in the Volta River basin area. Bull World Health Organ. 1986;64: 667–681. 3492300 PMC2490951

[20] Basáñez MG, Collins RC, Porter CH, Little MP, Brandling-Bennett D. Transmission intensity and the patterns of *Onchocerca volvulus* infection in human communities. Am J Trop Med Hyg. 2002;67(6): 669–679. 12518860 10.4269/ajtmh.2002.67.669

[21] Duerr HP, Leary CC, Eichner M. High infection rates at low transmission potentials in West African onchocerciasis. Int J Parasitol. 2006;36(13): 1367–1372. 16979644 10.1016/j.ijpara.2006.08.001

[22] Turner HC, Walker M, Churcher TS, Osei-Atweneboana MY, Biritwum NK, Hopkins A, et al Reaching the London Declaration on Neglected Tropical Diseases goals for onchocerciasis: an economic evaluation of increasing the frequency of ivermectin treatment in Africa. Clin Infect Dis. 2014;59(7): 923–932. 10.1093/cid/ciu467 24944228 PMC4166981

[23] African Programme for Onchocerciasis Control. Conceptual and operational framework of onchocerciasis elimination with ivermectin treatment World Health Organization: JAF 16.6 (II). 2010 [http://www.who.int/apoc/oncho_elimination_report_english.pdf]. Accessed 30 November 2015.

[24] Taylor MJ, Awadzi K, Basáñez MG, Biritwum N, Boakye D, Boatin B, et al Onchocerciasis control: vision for the future from a Ghanaian perspective. Parasit Vectors. 2009;2(1): 7 10.1186/1756-3305-2-7 19154624 PMC2639371

[25] Turner HC, Walker M, Attah SK, Opoku NO, Awadzi K, Kuesel AC, et al The potential impact of moxidectin on onchocerciasis elimination in Africa: an economic evaluation based on the Phase II clinical trial data. Parasit Vectors. 2015; 19;8: 167. 10.1186/s13071-015-0779-4 PMC4381491 25889256

[26] Coffeng LE, Stolk WA, Zouré HGM, Veerman JL, Agblewonu KB, Murdoch ME, et al African Programme for Onchocerciasis Control 1995–2015: model-estimated health impact and cost. PLoS Negl Trop Dis. 2013;7(1): e2032 10.1371/journal.pntd.0002032 23383355 PMC3561133

[27] Diggle PJ, Ribeiro PJ Jr. Model-based Geostatistics Springer Series in Statistics. New York: Springer; 2007.

[28] Karagiannis-Voules DA, Scholte RGC, Guimarães LH, Utzinger J, Vounatsou P. Bayesian geostatistical modeling of leishmaniasis incidence in Brazil. PLoS Negl Trop Dis. 2013;7(5): e2213 10.1371/journal.pntd.0002213 23675545 PMC3649962

[29] Brooker S. Spatial epidemiology of human schistosomiasis in Africa: risk models, transmission dynamics and control. Trans R Soc Trop Med Hyg. 2007;101(1): 1–8. 17055547 10.1016/j.trstmh.2006.08.004 PMC1975763

[30] Brooker S, Clements AC. Spatial heterogeneity of parasite co-infection: determinants and geostatistical prediction at regional scales. Int J Parasitol. 2009;39(5): 591–597. 10.1016/j.ijpara.2008.10.014 19073189 PMC2644303

[31] Magalhães RJ, Clements AC, Patil AP, Gething PW, Brooker S. The applications of model-based geostatistics in helminth epidemiology and control. Adv Parasitol. 2011;74: 267–296. 10.1016/B978-0-12-385897-9.00005-7 21295680 PMC3037997

[32] Chammartin F, Scholte RGC, Guimarães LH, Tanner M, Utzinger J, Vounatsou P. Soil-transmitted helminth infection in South America: a systematic review and geostatistical meta-analysis. Lancet Infect Dis. 2013;13(6): 507–518. 10.1016/S1473-3099(13)70071-9 23562238

[33] Patil AP, Gething PW, Piel FB, Hay SI. Bayesian geostatistics in health cartography: the perspective of malaria. Trends Parasitol. 2011;27(6): 246–253. 10.1016/j.pt.2011.01.003 21420361 PMC3109552

[34] Diggle PJ, Thomson MC, Christensen OF, Rowlingson B, Obsomer V, Gardon J, et al Spatial modelling and the prediction of *Loa loa* risk: decision making under uncertainty. Ann Trop Med Parasitol. 2007;101(6): 499–509. 17716433 10.1179/136485913X13789813917463

[35] Wardrop NA, Atkinson PM, Gething PW, Fèvre EM, Picozzi K, Kakembo AS, et al Bayesian geostatistical analysis and prediction of Rhodesian human African trypanosomiasis. PLoS Negl Trop Dis. 2010;4(12): e914 10.1371/journal.pntd.0000914 21200429 PMC3006141

[36] Slater H, Michael E. Mapping, Bayesian geostatistical analysis and spatial prediction of lymphatic filariasis prevalence in Africa. PLoS One. 2013;8(8): e71574 10.1371/journal.pone.0071574 23951194 PMC3741112

[37] Zouré HG, Noma M, Tekle AH, Amazigo UV, Diggle PJ, Giorgi E, et al The geographic distribution of onchocerciasis in the 20 participating countries of the African Programme for Onchocerciasis Control: (2) pre-control endemicity levels and estimated number infected. Parasit Vectors. 2014;7: 326 10.1186/1756-3305-7-326 25053392 PMC4222889

[38] Prost A, Thylefors B, Pairault C. Methods of mass epidemiological evaluation of onchocerciasis Their utilisation in a vector control programme. Geneva: World Health Organization ONCHO/WP/75.14. 1975.

[39] Kirkwood B, Smith P, Marshall T, Prost A. Variations in the prevalence and intensity of microfilarial infections by age, sex, place and time in the area of the Onchocerciasis Control Programme. Trans R Soc Trop Med Hyg. 1983;77(6): 857–861. 6665840 10.1016/0035-9203(83)90307-3

[40] Moreau JP, Prost A, Prod’hon J. Essai de normalisation de la méthodologie des enquêtes clinico-parasitologiques sur l’onchocercose en Afrique de l’ouest. Méd Trop (Mars). 1978;38(1): 43–51. 723550

[41] Vajime C, Quillévéré D. The distribution of the *Simulium damnosum* complex in West Africa with particular reference to the Onchocerciasis Control Programme area. Tropenmed Parasitol. 1978;29(4): 473–481. 741507

[42] Boakye DA, Back C, Fiasorgbor GK, Sib AP, Coulibaly Y. Sibling species distributions of the *Simulium damnosum* complex in the west African Onchocerciasis Control Programme area during the decade 1984–93, following intensive larviciding since 1974. Med Vet Entomol. 1998;12(4): 345–358. 9824818 10.1046/j.1365-2915.1998.00118.x

[43] Hijmans RJ, Cameron SE, Parra JL, Jones PG, Jarvis A. Very high resolution interpolated climate surfaces for global land areas. Int J Climatol. 2005;25(15): 1965–1978.

[44] Danielson JJ. Delineation of drainage basins from 1 km African digital elevation data. In: Pecora Thirteen, Human Interactions with the Environment–Perspectives from Space. Sioux Falls, South Dakota, August 20–22, 1996.

[45] Verdin KL, Verdin JP. A topological system for delineation and codification of the Earth’s river basins. J Hydrol. 1999; 218(1–2):1–12.

[46] Food and Agriculture Organization of the United Nations. FAO GEONETWORK. Rivers of Africa (Derived from HydroSHEDS) (GeoLayer). [http://www.fao.org/geonetwork/srv/en/metadata.show?id=31026]. Accessed 30 November 2015.

[47] Tucker CJ, Pinzon JE, Brown ME. Global Inventory Modeling and Mapping Studies, Normalized Difference Vegetation Index (NDVI), 2.0, Global Land Cover Facility, University of Maryland, College Park, Maryland, 01/07/1981–31/12/1986. 2004.

[48] Pinzon JE, Brown ME, Tucker CJ. EMD correction of orbital drift artifacts in satellite data stream In: Huang NE, Shen SSP, editors. Hilbert-Huang Transform and its Applications. Interdisciplinary Mathematical Sciences Volume 5, Singapore: World Scientific Publishing Co. Pte. Ltd.; 2005 pp: 167–186.

[49] Tucker CJ, Pinzon JE, Brown ME, Slayback D, Pak EW, Mahoney R, et al An extended AVHRR 8-km NDVI data set compatible with MODIS and SPOT vegetation NDVI data. Int J Remote Sens. 2005;26 (20): 4485–4498.

[50] NASA Land Processes Distributed Active Archive Center (LP DAAC). MODIS MOD13A1. USGS/Earth Resources Observation and Science (EROS) Center, Sioux Falls, South Dakota. 2001.

[51] NASA Land Processes Distributed Active Archive Center (LP DAAC). MODIS MOD1A2. USGS/Earth Resources Observation and Science (EROS) Center, Sioux Falls, South Dakota. 2001.

[52] NASA Land Processes Distributed Active Archive Center (LP DAAC). MODIS MCD12Q1. USGS/Earth Resources Observation and Science (EROS) Center, Sioux Falls, South Dakota. 2001.

[53] Friedl MA, Sulla-Menashe D, Tan B, Schneider A, Ramankutty N, Sibley A, et al MODIS Collection 5 global land cover: algorithm refinements and characterization of new datasets. Remote Sens Environ. 2010;114(1): 168–182.

[54] White, F. Vegetation of Africa—a descriptive memoir to accompany the Unesco/AETFAT/UNSO vegetation map of Africa; Natural Resources Research Report XX; U. N. Educational, Scientific and Cultural Organization; 7 Place de Fontenoy, 75700 Paris, France; 1983. 356 pp.

[55] US Geological Survey. Global 30-Arc-Second Elevation Data Set, Sioux Falls, South Dakota. 1996.

[56] Gesch DB, Larson KS. Techniques for development of global 1-kilometer digital elevation models. In: Pecora Thirteen, Human Interactions with the Environment—Perspectives from Space, 13th, Sioux Falls, South Dakota, August 20–22, 1996, Proceedings. Bethesda, Maryland: American Society of Photogrammetry and Remote Sensing. 1996.

[57] GLOBE Task Team and others. In: Hastings DA, Dunbar PK, Elphingstone GM, Bootz M, Murakami H, Maruyama H, Masaharu H, Holland P, Payne J, Bryant NA, Logan TL, Muller JP, Schreier G, MacDonald JS, editors. The Global Land One-kilometer Base Elevation (GLOBE) Digital Elevation Model, Version 1.0. National Oceanic and Atmospheric Administration, National Geophysical Data Center, 325 Broadway, Boulder, Colorado 80305–3328. 1999.

[58] Diggle PJ, Tawn JA, Moyeed RA. Model-based geostatistics. Appl Statist. 1998;47(3): 299–350.

[59] Christensen OS, Ribeiro PJ Jr. geoRglm: a package for generalised linear spatial models. R News. 2002;2: 26–28.

[60] R Development Core Team. R: a language and environment for statistical computing, version 3.0.1 The R Development Core Team. R Foundation for Statistical Computing 2014 [http://www.R-project.org/]. Accessed 23 June 2014.

[61] Austin PC, Tu JV. Bootstrap methods for developing predictive models. Am Stat. 2004;58(2): 131–137.

[62] Spiegelhalter DJ, Best NG, Carlin BP, van der Linde A. Bayesian measures of model complexity and fit. J R Statist Soc B. 2002;64(4): 583–639.

[63] Prost A, Hervouet JP, Thylefors B. [Epidemiologic status of onchocerciasis]. Bull World Health Organ. 1979;57(4): 655–662 [Article in French]. 316743 PMC2395839

[64] Hay SI, Guerra CA, Gething PW, Patil AP, Tatem AJ, Noor AM, et al A world malaria map: *Plasmodium falciparum* endemicity in 2007. PLoS Med. 2009;6(3): e1000048 Erratum in: PLoS Med. 2009;6(10). 10.1371/journal.pmed.1000048 19323591 PMC2659708

[65] Gething PW, Patil AP, Smith DL, Guerra CA, Elyazar IR, Johnston GL, et al A new world malaria map: *Plasmodium falciparum* endemicity in 2010. Malar J. 2011;10: 378 10.1186/1475-2875-10-378 22185615 PMC3274487

[66] Efron B, Tibshirani R. Improvements on cross-validation: the 632+ bootstrap method. J Am Statist Assoc. 1997;92 (438): 548–560.

[67] Hyndman RJ, Koehler AB. Another look at measures of forecast accuracy. Int J Forecast. 2006;22(4): 679–688.

[68] Gething PW, Noor AM, Gikandi PW, Hay SI, Nixon MS, Snow RW, et al Developing geostatistical space-time models to predict outpatient treatment burdens from incomplete national data. Geogr Anal. 2008;40: 167–188. 19325928 10.1111/j.1538-4632.2008.00718.x PMC2660576

[69] Center for International Earth Science Information Network (CIESIN), Columbia University; and Centro Internacional de Agricultura Tropical (CIAT). Gridded Population of the World Version 3 (GPWv3): Population Density Grids. Palisades, NY: Socioeconomic Data and Applications Center (SEDAC), Columbia University 2005 [http://sedac.ciesin.columbia.edu/data/set/gpw-v3-population-density]. Accessed 23 June 2015.

[70] United Nations. World Population Prospects: The 2012 Revision, DVD edition. New York: UN Department of Economic and Social Affairs, Population Division Highlights and advance tables. 2013 [http://esa.un.org/wpp/Documentation/pdf/WPP2012_HIGHLIGHTS.pdf]. Accessed 30 November 2015.

[71] Center for International Earth Science Information Network (CIESIN), Columbia University; International Food Policy Research Institute (IFPRI); the World Bank; and Centro Internacional de Agricultura Tropical (CIAT). Global Rural-Urban Mapping Project, Version 1 (GRUMPv1): Urban Extents Grid. Palisades, NY: Socioeconomic Data and Applications Center (SEDAC), Columbia University 2011 [http://sedac.ciesin.columbia.edu/data/set/grump-v1-urban-extents]. Accessed 30 November 2015.

[72] Frentzel-Beyme R. The geographical distribution of *Onchocerca volvulus* infection in Liberia. Tropenmed Parasitol. 1975;26(1): 70–87. 1145727

[73] Coffeng LE, Pion SDS, O'Hanlon S, Cousens S, Abiose AO, Fischer PU, et al Onchocerciasis: the pre-control association between prevalence of palpable nodules and skin microfilariae. PLoS Negl Trop Dis. 2013;7(4): e2168 10.1371/journal.pntd.0002168 23593528 PMC3623701

[74] Wilson MD, Cheke RA, Flasse SPJ, Grist S, Osei-Atweneboana MY, Tetteh-Kumah A, et al Deforestation and the spatio-temporal distribution of savannah and forest members of the *Simulium damnosum* complex in southern Ghana and south-western Togo. Trans R Soc Trop Med Hyg. 2002;96(6): 632–639. 12625139 10.1016/s0035-9203(02)90335-4

[75] Osei-Atweneboana MY, Eng JK, Boakye DA, Gyapong JO, Prichard RK. Prevalence and intensity of *Onchocerca volvulus* infection and efficacy of ivermectin in endemic communities in Ghana: a two-phase epidemiological study. Lancet. 2007;369(9578): 2021–2029. 17574093 10.1016/S0140-6736(07)60942-8

[76] Lamberton PHL, Cheke RA, Walker M, Winskill P, Osei-Atweneboana MY, Tirados I, et al Onchocerciasis transmission in Ghana: biting and parous rates of host-seeking sibling species of the *Simulium damnosum* complex. Parasit Vectors. 2014;7: 511 10.1186/s13071-014-0511-9 25413569 PMC4247625

[77] Lamberton PHL, Cheke RA, Winskill P, Tirados I, Walker M, Osei-Atweneboana MY, et al Onchocerciasis transmission in Ghana: persistence under different control strategies and the role of the simuliid vectors. PLoS Negl Trop Dis. 2015;9(4): e0003688. 10.1371/journal.pntd.0003688 25897492 PMC4405193

[78] Rolland A, Balay G. L’onchocercose dans le foyer Bissa. Mimeographed document 111/ONCHO. Bobo-Dioulasso: Organisation de Coordination et de Coopération pour la Lutte contre les Grandes Endémies. 1969. [English translation published by the Onchocerciasis Control Programme, Ouagadougou, in 1976].

[79] De Sole G, Giese J, Keita FM, Remme J. Detailed epidemiological mapping of three onchocerciasis foci in West Africa. Acta Trop. 1991;48(3): 203–213. 1671622 10.1016/0001-706x(91)90048-o

[80] Samba EM (1994) The Onchocerciasis Control Programme in West Africa: an example of effective public health management Public Health in Action 1, Geneva: World Health Organization 1994 [http://apps.who.int/iris/bitstream/10665/39261/1/WHO_PHA_1.pdf]. Accessed 30 November 2015.

[81] Renz A, Wenk P. Studies on the dynamics of transmission of onchocerciasis in a Sudan savanna area of North Cameroon. I. Prevailing *Simulium* vectors, their biting rates and age composition at different distances from their breeding sites. Ann Trop Med Parasitol. 1987;81(3): 215–228. 3662664 10.1080/00034983.1987.11812115

[82] Duke BOL, Moore PJ, Anderson J. Studies on factors influencing the transmission of onchocerciasis. VII. A comparison of the *Onchocerca volvulus* transmission potentials of *Simulium damnosum* populations in four Cameroon rain-forest villages and the pattern of onchocerciasis associated therewith. Ann Trop Med Parasitol. 1972;66(2): 219–234. 5038247

[83] Davies JB, Beech-Garwood PA, Thomson MC, McMahon JE. Onchocerciasis transmission levels and *Simulium damnosum* complex biting activity at riverside and rice field sites in Sierra Leone. Med Vet Entomol. 1988;2(4): 357–369. 2980195 10.1111/j.1365-2915.1988.tb00209.x

[84] Bockarie MJ, Davies JB, Thomson MC, Morgan HG. The transmission of onchocerciasis at a forest village in Sierra Leone. I. *Simulium damnosum* s.l. biting densities and infection with *Onchocerca volvulus* at five representative sites. Ann Trop Med Parasitol. 1990;84(6): 587–597. 2076037 10.1080/00034983.1990.11812514

[85] Garms R, Walsh JF, Davies JB. Studies on the reinvasion of the Onchocerciasis Control Programme in the Volta River Basin by *Simulium damnosum* s.l. with emphasis on the south-western areas. Tropenmed Parasitol. 1979;30(3): 345–362. 575581

[86] Dadzie KY, Remme J, Baker RH, Rolland A, Thylefors B. Ocular onchocerciasis and intensity of infection in the community. III. West African rainforest foci of the vector *Simulium sanctipauli*. Trop Med Parasitol. 1990;41(4): 376–382 1963702

[87] Dadzie KY, Remme J, Rolland A, Thylefors B. Ocular onchocerciasis and intensity of infection in the community. II. West African rainforest foci of the vector *Simulium yahense*. Trop Med Parasitol. 1989;40(3): 348–354 2559472

[88] Remme J, Dadzie KY, Rolland A, Thylefors, B. Ocular onchocerciasis and intensity of infection in the community. I. West African savanna. Trop Med Parasitol. 1989; 40(3): 340–347 2617045

[89] Jacob BG, Novak RJ, Toe LD, Sanfo M, Griffith DA, Lakwo TL, et al Validation of a remote sensing model to identify *Simulium damnosum* s.l. breeding sites in Sub-Saharan Africa. PLoS Negl Trop Dis. 2013;7(7): e2342 10.1371/journal.pntd.0002342 23936571 PMC3723572

[90] Ngoumou P, Walsh JF, Macé JM. A rapid mapping technique for the prevalence and distribution of onchocerciasis: a Cameroon case study. Ann Trop Med Parasitol. 1994;88(5): 463–474. 7979636 10.1080/00034983.1994.11812893

[91] Macé JM, Boussinesq M, Ngoumou P, Enyegue Oye J, Koéranga A, Godin C. Country-wide rapid epidemiological mapping of onchocerciasis (REMO) in Cameroon. Ann Trop Med Parasitol. 1997;91(4): 379–391. 9290845

[92] Cheke RA, Basáñez MG, Perry M, White MT, Garms R, Obuobie E, et al Potential effects of warmer worms and vectors on onchocerciasis transmission in West Africa. Philos Trans R Soc Lond B Biol Sci. 2015; 370(1665): 20130559 10.1098/rstb.2013.0559 25688018 PMC4342963

[93] Mendoza-Aldana J, Piechulek H, Maguire J. Forest onchocerciasis in Cameroon: its distribution and implications for selection of communities for control programmes. Ann Trop Med Parasitol. 1997;91(1): 79–86. 9093432 10.1080/00034983.1997.11813114

[94] Vivas-Martínez S, Basáñez MG, Grillet ME, Weiss H, Botto C, García M, et al Onchocerciasis in the Amazonian focus of southern Venezuela: altitude and blackfly species composition as predictors of endemicity to select communities for ivermectin control programmes. Trans R Soc Trop Med Hyg. 1998;92(6): 613–620. 10326102 10.1016/s0035-9203(98)90784-2

[95] Tada I, Aoki Y, Rimola CE, Ikeda T, Matsuo F, Ochoa JO, et al Onchocerciasis in San Vicente Pacaya, Guatemala. Am J Trop Med Hyg. 1979;28(1): 67–71. 434316 10.4269/ajtmh.1979.28.67

[96] Carabin H, Escalona M, Marshall C, Vivas-Martínez S, Botto C, Joseph L, et al Prediction of community prevalence of human onchocerciasis in the Amazonian focus: Bayesian approach. Bull World Health Organ. 2003;81(7): 482–490. 12973640 PMC2572496

[97] Botto C, Villamizar NJ, Jocik Z, Grillet ME, Basáñez MG. Landscape epidemiology of human onchocerciasis in southern Venezuela In: Nriagu J, editor-in-chief. Encyclopaedia of Environmental Health. Oxford: Elsevier; 2011 pp: 366–379.

[98] Garske T1, Ferguson NM, Ghani AC. Estimating air temperature and its influence on malaria transmission across Africa. PLoS One. 2013;8(2): e56487 10.1371/journal.pone.0056487 23437143 PMC3577915

[99] World Health Organization. World Health Organization Expert Committee on Onchocerciasis. Third Report. Technical Report Series No. 752. Geneva: WHO. 1987. [http://apps.who.int/iris/bitstream/10665/38959/1/WHO_TRS_752_(part1).pdf] and [http://apps.who.int/iris/bitstream/10665/38959/2/WHO_TRS_752_(part2).pdf], accessed 30 November 2015. 3120423

[100] De Sole G, Baker R, Dadzie KY, Giese J, Guillet P, Keita FM, et al Onchocerciasis distribution and severity in five West African countries. Bull World Health Organ. 1991; 69(6): 689–698. 1786617 PMC2393320

[101] De Sole G, Accorsi S, Cresveaux H, Remme J, Walsh F, Hendrickx J. Distribution and severity of onchocerciasis in southern Benin, Ghana and Togo. Acta Trop. 1992;52(2–3): 87–97. 1363185 10.1016/0001-706x(92)90024-r

[102] Amazigo U, Noma M, Bump J, Benton B, Liese B, Yaméogo L, et al Onchocerciasis (Chapter 15) In: Jamison DT, Feachem RG, Makgoba MW, Bos ER, Baingana FK, Hofman KJ, Rogo KO, editors. Disease and Mortality in Sub-Saharan Africa. 2nd edition Washington DC: World Bank; 2006 pp: 215–222.

[103] Basáñez MG, McCarthy J, French MD, Yang GJ, Walker M, Gambhir M, et al A research agenda for helminth diseases of humans: modelling for control and elimination. PLoS Negl Trop Dis. 2012;6(4): e1548 10.1371/journal.pntd.0001548 22545162 PMC3335861

[104] Turner HC, Walker M, Churcher TS, Basáñez MG. Modelling the impact of ivermectin on River Blindness and its burden of morbidity and mortality in African savannah: EpiOncho projections. Parasit Vectors. 2014;7(1): 241. 24886747 10.1186/1756-3305-7-241 PMC4037555

[105] Noma M, Nwoke BEB, Nutall I, Tambala PA, Enyong P, Namsenmo A, et al Rapid epidemiological mapping of onchocerciasis (REMO): its application by the African Programme for Onchocerciasis Control. Ann Trop Med Parasitol. 2002;96 (Suppl 1): S29–S39. 12081248 10.1179/000349802125000637

[106] Thomson MC, Obsomer V, Dunne M, Connor SJ, Molyneux DH. Satellite mapping of *Loa loa* prevalence in relation to ivermectin use in west and central Africa. Lancet. 2000; 356(9235): 1077–1078. Erratum in Lancet. 2000; 356(9236): 1198. 11009145 10.1016/s0140-6736(00)02733-1

[107] Zouré HGM, Wanji S, Noma M, Amazigo UV, Diggle PJ, Tekle AH, et al The geographic distribution of *Loa loa* in Africa: results of large-scale implementation of the Rapid Assessment Procedure for Loiasis (RAPLOA). PLoS Negl Trop Dis. 2011;5(6): e1210 10.1371/journal.pntd.0001210 21738809 PMC3125145

[108] Tekle AH, Zouré H, Wanji S, Leak S, Noma M, Remme JH, et al Integrated rapid mapping of onchocerciasis and loiasis in the Democratic Republic of Congo: impact on control strategies. Acta Trop. 2011;120 (Suppl 1): S81–S90. 10.1016/j.actatropica.2010.05.008 20525531

[109] Chippaux JP, Boussinesq M, Gardon J, Gardon-Wendel N, Ernould JC Severe adverse reaction risks during mass treatment with ivermectin in loiasis endemic areas. Parasitol Today. 1996;12(11): 448–450. 15275280 10.1016/0169-4758(96)40006-0

[110] Gardon J, Gardon-Wendel N, Demanga-Ngangue, Kamgno J, Chippaux JP, Boussinesq M. Serious reactions after mass treatment of onchocerciasis with ivermectin in an area endemic for *Loa loa* infection. Lancet. 1997;350(9070): 18–22. 9217715 10.1016/S0140-6736(96)11094-1

