# Supplementary material for: Model-Based Geostatistical Mapping of the Prevalence of Onchocerca volvulus in West Africa
Source: PLoS Negl Trop Dis. 2016 Jan 15;10(1):e0004328. doi: 10.1371/journal.pntd.0004328 (PMC4714852; doi:10.1371/journal.pntd.0004328)
Supplement: S1 File — Text A. Data description and pre-control data selection criteria. Text B. Parasitological survey methods and geographical limits of the mapping. Text C. Processing of environmental covariates. Text D. Maps of environmental covariates. Text E. K-folds model validation. Text F. MCMC chain diagnostics. Text G. Posterior distributions of model parameters. Text H. Further maps derived from the predictive posterior distributions. Text I. Geospatial software. Table A. Population growth rates, factor by which the 1990 population count was multiplied for back-calculation to 1975, and proportion of the population aged 0–4 years for OCP countries. Fig A. Village locations in the OCP epidemiological database. Fig B. Location of the data points selected for use in this study after applying pre-control selection criteria. Fig C. Scatterplot of age- and sex-standardised microfilarial prevalence in those aged 5 years and above (Pmf), against the crude, unadjusted prevalence. Fig D. Heatmap of distance (in Km) to nearest pre-control survey data point for each map pixel in the study area. Fig E. Lambert Azimuthal Equal Area projection of the world, centred on Africa. Fig F.1. Maps for covariate values at prediction locations, for NDVI βj covariates included in the final predictive model. Fig F.2. Maps for covariate values at prediction locations, for all other βj covariates included in the final predictive model. Fig G. The location of validation points in each K-fold cross-validation dataset. Fig H. MCMC traceplots of all βj regression coefficients. Fig I. MCMC traceplot of the value of the range parameter, ϕ. Fig J. Highest posterior density (HPD) plots of model parameters. Fig K. Mean of predictive posterior distributions for estimated microfilarial prevalence of Onchocerca volvulus prior to the commencement of the Onchocerciasis Control Programme in West Africa (OCP) in 1975. Fig L. Signal to noise ratio between mean and standard deviation of predictive posteriors. Fig M. Histogr [file pntd.0004328.s001.pdf]

Model-Based Geostatistical Mapping of the Prevalence of *Onchocerca volvulus* in West Africa

Supplementary Information S1 File. Detailed description of data and methods

Simon J. O'Hanlon[1*], Hannah C. Slater[1,2], Robert A. Cheke[1,3], Boakye A. Boatin[4], Luc E. Coffeng[5], Sébastien D.S. Pion[6], Michel Boussinesq[6], Honorat G. M. Zouré[7], Wilma A. Stolk[5], and María-Gloria Basáñez[1,8*]

[1] Department of Infectious Disease Epidemiology, School of Public Health, Faculty of Medicine (St Mary's campus), Imperial College London, Norfolk Place, London W2 1PG, UK

[2] MRC Centre for Outbreak Analysis & Modelling, Department of Infectious Disease Epidemiology, Imperial College London, London W2 1PG, UK

[3] Natural Resources Institute, University of Greenwich at Medway, Central Avenue, Chatham Maritime, Chatham, Kent ME4 4TB, UK

[4] Lymphatic Filariasis Support Centre, Department of Parasitology, Noguchi Memorial Institute for Medical Research, University of Ghana, Legon, Ghana

[5] Department of Public Health, Erasmus MC, University Medical Center Rotterdam, PO Box 2040, 3000 CA, Rotterdam, The Netherlands

[6] UMI 233, Institut de Recherche pour le Développement (IRD) and University of Montpellier 1, 911 avenue Agropolis, BP 64501. 34394 Montpellier Cedex 5, France

[7] London Centre for Neglected Tropical Disease Research, Department of Infectious Disease Epidemiology, Imperial College London, London W2 1PG, UK

* Corresponding authors: simon.ohanlon@gmail.com (SOH); m.basanez@imperial.ac.uk (MGB)

Text A. Data description and pre-control data selection criteria

The OCP epidemiological database contains 5,816 surveys carried out at 2,581 geo-referenced villages in the 11 participating countries in West Africa, spread across the 9 OCP operational phases. A total of 331 uniquely named locations, where 393 parasitological surveys were carried out, were missing geo-referencing information or were coded with inaccurate geo-references (208 locations were coded with X/Y coordinates of –1/–1 or 0/0). Fig A depicts the locations of all village sites in the database with valid, unique geo-references, stratified by operational phase.

A further 175 locations had missing data for numbers of persons examined and/or numbers of persons positive for skin microfilariae of *Onchocerca volvulus* at each site (data coded as '-1'). After trimming these data, 5,248 surveys at 2,224 unique locations within the OCP remained. These surveys were divided between phases whose villages received primarily mass treatment with ivermectin and those where vector control was the chief method of intervention between 1975 and approximately 1990. There were 2,525 surveys in OCP phases with antiparasitic intervention (ivermectin) and 2,723 surveys in phases with antivectorial intervention (larviciding of vector breeding sites).

Each operational phase of the OCP (Fig 1B Main Text) comprised a number of contiguous river basins or watersheds. The OCP database contains data on the start date of control within each river basin, as well as the overall start date for that operational phase (usually the same as the earliest date of control in any river basin within that phase). The initiation of control was not implemented simultaneously across all river basins in a single phase. The size of a river basin referred to in the OCP dataset may be quite small and sometimes refers to a small tributary which is part of a larger contiguous hydrological watershed. Therefore, control would have been quite localised. Some river basins did not receive control until well after the start of control in other river basins within that phase, e.g. in Phase I the start of control ranged from February 1975 to March 1979. Hence, the start date of control of the river basin in which a village was located was used to define the criteria for selecting pre-control surveys.

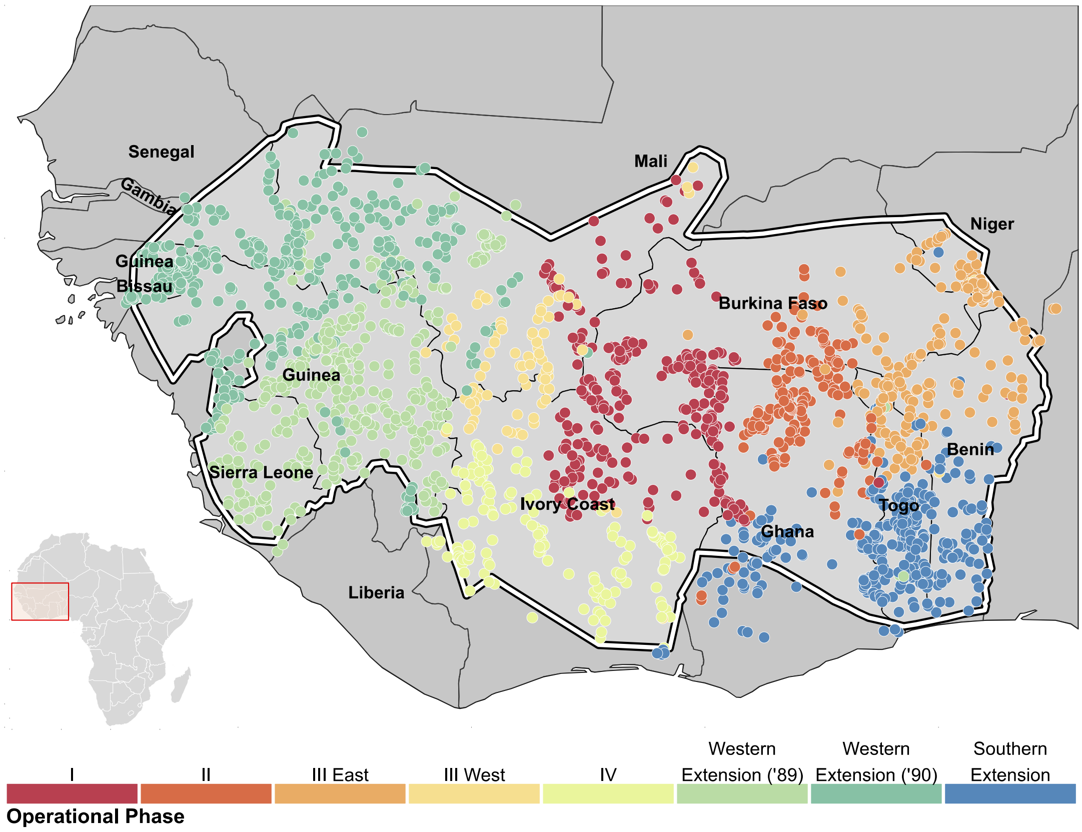

Fig A. Village locations in the OCP epidemiological database. The position of all villages within the OCP dataset with valid geo-referencing data. The data are coloured according to the operational phase that is listed for that village in the database. The white border indicates the limits of the OCP (some surveys were conducted outside these).

In the phases that started with vector larviciding (Phases I, II, III-East, III-West and IV) the criteria were relaxed that all surveys must have been carried out before the start of control because—prior to the introduction of ivermectin treatment—reductions in microfilarial prevalence would have been very slow at first, especially in hyperendemic areas, as illustrated in [1]. For this reason, in all phases where vector control was the primary method of control, surveys (a total of 443) that were conducted within 3 years of the start date of control for that river basin were included. Finally, at locations where there was more than one survey available, the earliest of the surveys were considered to be the most representative of the pre-control endemic equilibrium, leaving 336 pre-control data points in these phases.

In phases that commenced with mass ivermectin treatment (the Western Extension and Southern Extension Phases), selection was restricted to those surveys conducted prior to the start date of control within river basins. Ivermectin is an effective microfilaricide [2], effecting changes in the

endemic microfilarial prevalence more quickly than vector control. A total of 453 surveys were eligible in these phases and, as described earlier, at sites where multiple parasitological surveys were available, the earliest surveys were selected as the most representative of initial microfilarial prevalence. Applying this criterion left 401 surveys available in these phases, which added to the 336 surveys in vector control phases totalled 737 data points overall (Fig 1C, Main Text). Fig B plots the locations of the selected villages, stratified by operational phase, after cleaning the data for errors due to miscoded phases or erroneous coordinates. The villages cluster within phases with very little evident intermixing between phases, perhaps with the exception of the Western Extension phases. However, these phases started control at the same time. The distribution of pre-control points shows good congruence with the distribution of all data points.

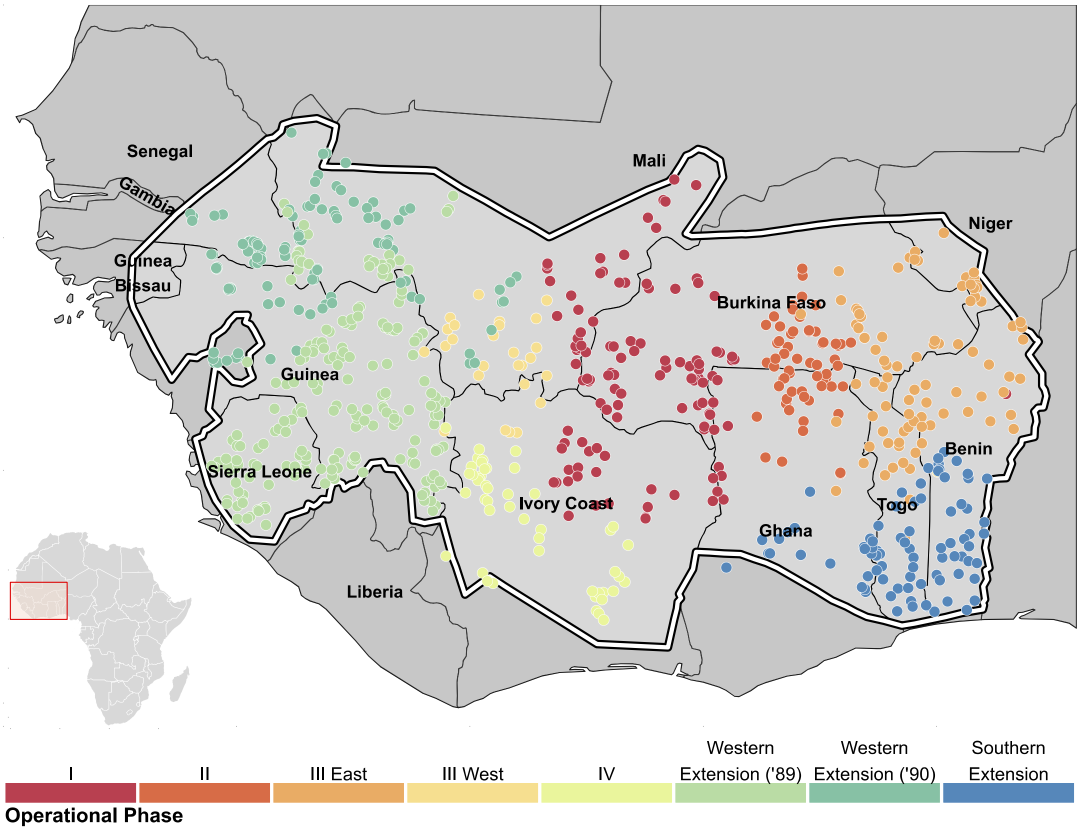

Fig B. Location of the data points selected for use in this study after applying pre-control selection criteria. The data ($n = 737$ villages) are stratified by OCP operational phase. The spatial distribution of the selected points is similar to the distribution of all villages in the main dataset. The white border indicates the limits of the OCP. (Although there was no vector control in Guinea Bissau and ivermectin treatment only started in 1991, data from this country were excluded because of erroneous geo-referencing of some villages.)

Text B. Parasitological survey methods and geographical limits of the mapping

The methods used in the epidemiological surveys of the OCP have been previously described [3]. At each survey a complete census of the village was conducted, and approximately 84% of persons enumerated in the census were examined [4]. Parasitological assessment comprised taking bloodless skin snips (with a 2-mm Holth-type corneoscleral punch), one from the right and one from the left iliac crests. Biopsies taken with this punch are relatively similar in size, weighing between 1 and 3 mg [5]. The skin snips were placed in distilled water for 30 minutes and examined by microscopy for presence (and number) of emerged microfilariae. Negative snips were re-incubated for up to 24 hours in saline solution and examined again. Reported prevalence at the village-level was age- and sex-standardised to the OCP reference population [3]. Due to the relatively invasive nature of the biopsy and the low prevalence expected in individuals aged below 5 yrs, skin snipping was only performed in the population aged 5 years and above. Standardised prevalence data were available in the database, but age- and sex-adjusted counts of positive persons were not. It would have been possible to back-calculate these figures, but although standardised prevalence was provided by sex, the data by age-group were not available. The Pearson's correlation between the standardised and unadjusted prevalence (Fig C below) was very high ($r^2 = 0.95$) and the relationship was roughly linear; therefore, the unadjusted count data were used for this study.

Our ground-truth data points, consisting of parasitological survey data from the OCP epidemiological database, were located almost entirely within the boundaries of the OCP, as depicted in Fig 1C of the Main Text (see also Fig D below for a heatmap of distance of map pixels to the nearest input, ground-truth data point within the study area).

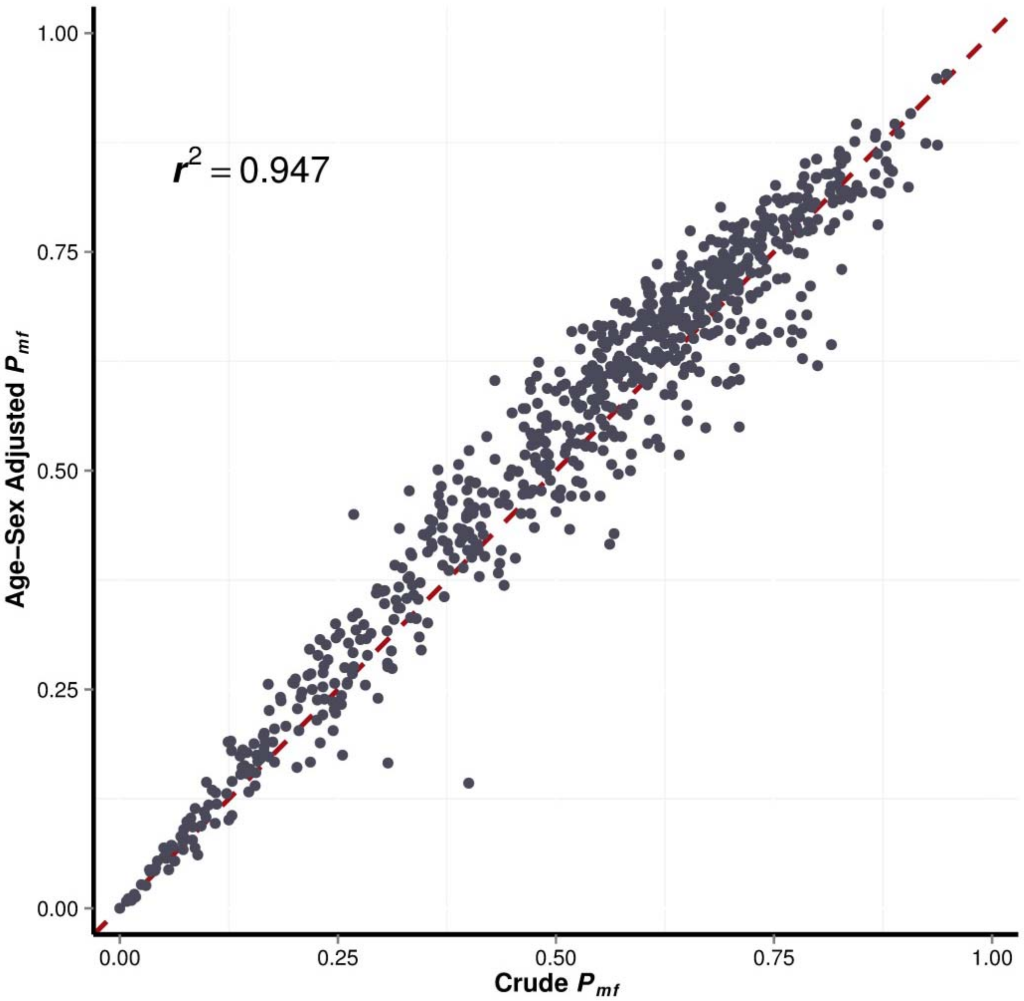

Fig C. Scatterplot of age- and sex-standardised microfilarial prevalence in those aged 5 years and above (P_{mf}), against the crude, unadjusted prevalence. Unadjusted prevalence is the total number of persons positive for skin *O. volvulus* microfilariae divided by total number of persons examined. The determination coefficient is 0.95 (Pearson's correlation coefficient equal to 0.973). The red dashed line represents the line of perfect correlation.

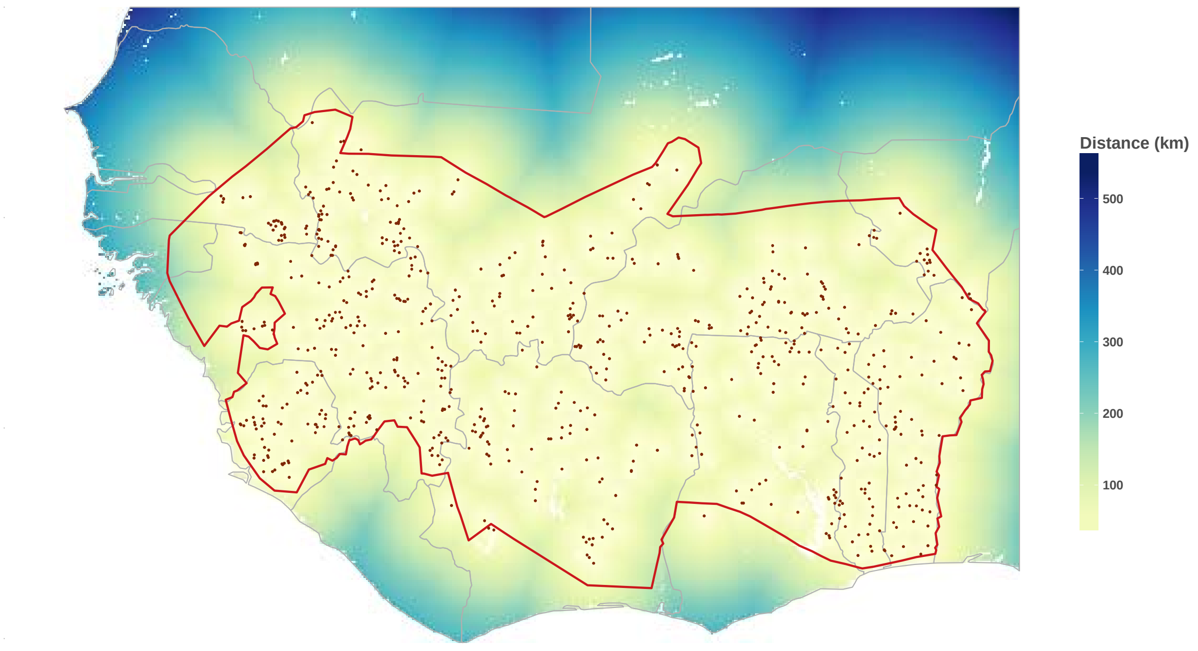

Fig D. Heatmap of distance (in Km) to nearest pre-control survey data point for each map pixel in the study area. Without pseudo-absence points (see Main Text), there is a large distance to nearest ground-truth data points north of the northerly limits of the OCP.

Text C. Processing of environmental covariates

An ensemble of potential explanatory environmental covariates was assembled from a variety of data sources, at a variety of spatial resolutions and differing map projections. Selection of environmental covariates was initially guided by those deemed to have a known influence on the epidemiology of onchocerciasis (e.g. distance between villages and vector breeding sites in rivers). In addition, values for covariates had to be available at each of the prediction locations in the study area, with data ideally available at a spatial resolution of 5 km or better. The full list of environmental covariates that were assessed is presented in Table 2 in the Main Text. This section describes the data and the spatial re-projection and sampling that was carried out to generate values of environmental covariates at input data locations and continuous maps across prediction locations. For the biological relevance of including any of these covariates in a predictive model of onchocerciasis prevalence, see the 'Discussion' section in the Main Text.

Sampling covariate layers at data locations

Sampling of environmental covariate values at input data locations was done in the native projection of each covariate layer to minimise error introduced by distortion from re-projecting a regular grid of raster data, usually in a geographic coordinate system, to a projected coordinate system. The coordinates of the data locations were instead re-projected (there is no distortion in the re-projection of a single point in space from one geographic coordinate system to another) to match the projection of each covariate layer in turn. Values were then sampled from each covariate layer at the data locations, before the input data locations and all covariate layers were finally transformed to the common projection of the prediction grid.

The projection of the prediction grid chosen was the Lambert Azimuthal Equal Area projection (Fig E), which is especially suitable for spatial data across continental and regional scales. One of the benefits of this projection is the accurate representation of distances and area at the expense of angular representation (see Fig K in section H 'Further maps derived from the predictive posterior distributions'). In addition the datum of the projection, typically in metres or kilometres, is easier to interpret in terms of model outputs than decimal degrees. The specifications of the standard prediction grid were:

```
rows : 303, columns: 460, ncells: 139380
resolution (Km): 5, 5  (x, y)
extent: -4000, -1700, -15, 1500  (xmin, xmax, ymin, ymax)
proj4string: +proj=laea +lon_0=20 +lat_0=5 +ellps=sphere +to_meter=1e3
```

The total number of cells in the prediction grid included cells that overlay water bodies, the sea and urban extents (as defined using the GRUMPv1.0 data described below). We did not predict in these cells, so the total number of prediction points was 112,295.

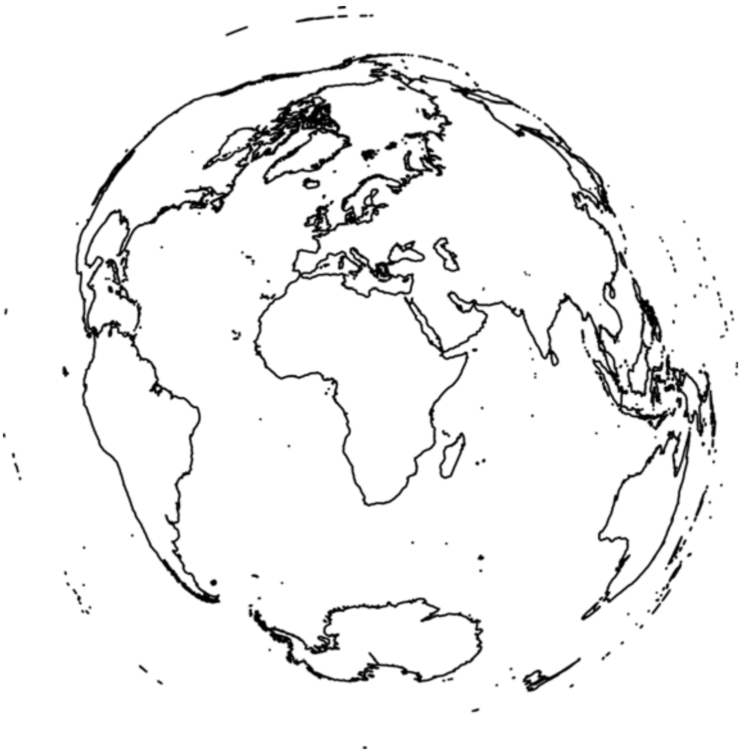

Fig E. Lambert Azimuthal Equal Area projection of the world, centred on Africa. This projection was used in this study as it is especially suitable for the representation of spatial data across continental and regional scales.

WorldClim Data [6]

The data are a set of global-coverage raster maps of climatic conditions with a spatial resolution of 1km. They consist of temporally aggregated data (1950–2000) for average monthly minimum temperature, maximum temperature, mean temperature and precipitation. There are also a set of 19 bioclimatic variables derived from these temperature and precipitation data, which are described at http://www.worldclim.org/bioclim. The temperature data (in degrees Celsius, °C) are supplied as integer data and must be converted to floating point by multiplying by a scaling factor, z, with $z = 0.1$. The units of the precipitation layers are in millimetres (mm). The derived bioclimatic layers (termed Bioclim variables) are more biologically meaningful variables. Examples include precipitation seasonality (the intra-year variability of rainfall, with higher values indicating that rainfall is more seasonal); mean temperature of the wettest quarter (on a pixel-by-pixel basis, this is the mean temperature for the 12 contiguous weeks that had the highest average rainfall in the year; for neighbouring pixels the 12 contiguous weeks could be at different time-points during the year), and

mean diurnal temperature range (the mean of monthly difference between the maximum and minimum temperature).

Hydro1K Database [7]

Hydrologic covariates were extracted from the Hydro1K database. These layers consist of a number of raster maps, derived from a digital elevation model and a vector shapefile of streamlines based on the elevation model. We extracted elevation, stream lines, compound topographic index (CTI, a measure of groundwater flow, often called the wetness index), flow direction (direction of flow from each cell in the raster to its steepest down-slope neighbour) and flow accumulation (the upstream catchment area for a given pixel). The stream lines data were used to calculate a raster of Euclidean distance to the nearest stream segment, and to derive a raster of gradient of the nearest stream segment as a proxy for flow speed of the nearest river segment. Blackfly larvae survive in rivers with discharge volumes in the range of 0.1–150 cubic metres per second, and stream gradient affects flow rate .The CTI or topographic wetness index layer, quantifies how topographic variation affects hydrological processes. Ground water flow often follows surface topography [8], which can also affect river discharge volumes and flow rates, and hence the location of suitable blackfly breeding sites. Following Beven and Kirkby [9], the CTI for any given location i, λ_i, is calculated as $\lambda_i = \ln\left(\frac{a_i}{\tan b_i}\right)$ where a_i is the local upstream area which flows into location i, and b_i is the surface slope angle between location i and its neighbouring locations (i.e. the 8 surrounding cells).

MODIS Satellite Data Products [10-12]

<u>MOD13Q1 MODIS data product</u>

The MOD13Q1 MODIS data product contains layers for vegetation indices. Both the Normalised Difference Vegetation Index (NDVI) and the Enhanced Vegetation Index (EVI) were extracted from this dataset. The data are provided at a high resolution of 250m, in 16-day means (i.e. one raster consisting of the mean values for the previous 16 days). We downloaded all available data at the time

(from 2000.02.18–2013.07.12), consisting of 309 sets of raster layers. The data are supplied at integer values (to reduce storage and data transmission requirements), needing to be multiplied by a scaling factor, $z = 0.0001$. Each raster was sorted into ecological quarters based on year-day of the dataset, with ecological quarters defined as in the Main Text. The long-term per-pixel mean was then calculated across all raster layers in each ecological quarter, resulting in 8 high resolution layers; one for each quarter of NDVI and EVI respectively. After sampling values at input data locations at high resolution, these layers were aggregated to 5km spatial resolution using a bilinear interpolation and re-projected to the same extent and resolution as the prediction grid.

MOD11A2 data product

The MOD11A2 data product consists of Land Surface Emissivity data. We used the daytime and night-time landsurface temperature (LST) layers which were provided at 1km spatial resolution, as 8-day averages. Temporal aggregation, data sampling, spatial aggregation and re-projection were carried out as described above for the MOD13Q1 MODIS data product, with the exception that the scaling factor was $z = 0.02$ and that the data were converted from Kelvin to Celsius degrees by subtracting 273.15.

MCD12Q1 data product

The MCD12Q1 data product contains Yearly Land Cover Type at a spatial resolution of 500m. The data are supplied as a yearly classification with 5 schemes available. We used the International Geosphere-Biosphere Programme classification system [13]. Data were downloaded for each of the available years (2001–2012) and aggregated by using the per-pixel mode of land type, suitable for categorical data. This method of aggregation was chosen instead of using the earliest available data layer (2001) because there was a period of 26 years between the desired time point (1975) and the earliest available data. Using the mode for each pixel was deemed most likely to capture the true land-use type for that pixel and eliminate any anomalous classification derived from using the earliest raster by itself, e.g. as a result of a forest fire, giving a classification that would not consistent with the long-term use of land cover in that pixel. Aggregation from 500m to 5km resolution was performed,

with the larger pixels also taking the modal value of the one hundred 500m pixels contained in each 5km pixel.

Global Inventory Modelling and Mapping Studies NDVI [14-16]

The NDVI layer from the Global Inventory Modelling and Mapping Studies (GIMMS) database is derived from much earlier satellite data of the Advanced Very High Resolution Radiometer (AVHRR) satellites of the National Oceanic and Atmospheric Administration (NOAA). Continuous sampling at an 8km resolution is available from 1982 to 2006; however, at present the data are listed as 'not available' on the Global Land Cover Facility Website[1]. The data consist of 616 raster layers covering the African continent in twice-monthly averages. The raster data were sampled at input data locations in the native resolution before being resampled. This dataset was downloaded and used because the temporal coverage is much closer to the start date of control of the OCP. However, the low spatial resolution hinders its utility when looking at spatial relationships on a finer scale. In addition, GIMMS NDVI was not found to be as good a univariate predictor of microfilarial prevalence as the high-resolution MODIS data, i.e. models using GIMMS NDVI had a higher—worse fit—DIC value than models using MODIS NDVI or EVI. As GIMMS NDVI was highly correlated with MODIS NDVI and MODIS EVI), this data layer was not taken forward in further analyses.

HydroSheds [17]

The Rivers of Africa dataset is derived from HydroSheds, a mapping product of the World Wildlife Fund (WWF). The data are based on NASA's Shuttle Radar Topographic Mission (SRTM) 15-second Digital Elevation Model (DEM) and consist of a drainage direction layer and a stream network layer. The stream network consists of a network of spatial lines with attribute information for each line segment. In addition to the spatial location of the stream segments, attributes which were thought to

[1] *The authors hold a copy of the processed GIMMS NDVI database for the African continent, covering the period July 1981-November 2006. We would be pleased to provide freely the dataset upon request on a case-by-case basis, on the condition we are given explicit permission by the Global Land Cover Facility, who originally hosted the data.*

be relevant to this mapping study were the classification of stream segments as perennial or intermittent streams (intermittent streams do not flow throughout the year), and the Strahler order (hydrological rank) of river segments. Strahler's stream order system [18] proposes that a river or stream segment be given a rank according to the number of upstream tributaries it possesses. Therefore a 1st order stream segment has no upstream tributaries and is known as a headstream. As rank increases so does the size of a river (as more tributaries feed into it), which can give an indication of the magnitude or flow rate of the river in that location compared to the upstream area.

White's Vegetation Map of Africa [19]

As well as remotely sensed land cover data (see MCD12Q1 data product), we also evaluated the use of a map of major vegetation types based on physiognomy, not climate data. The data in this layer had been compiled from existing maps and verified though fieldwork and local expert knowledge. The morphological types and floristic composition of endemic species were used to define vegetation classifications. The data are supplied as a single raster, which was sampled at native resolution (1km) before being aggregated and re-projected to the prediction grid using the same methods as those described above for the MODIS MCD12Q1 Land Cover dataset.

GTOPO30 [20,21] and GLOBE [22] Digital Elevation Models

There are multiple altitude datasets available. We assessed the use of two DEMs, the Global 30 Arc-Second Elevation (GTOPO30) model and the Global Land One-km Base Elevation Project (GLOBE) model. Both GTOPO30 and GLOBE provide global coverage of topographic relief data at 1km resolution. After sampling the value of altitude at input data locations, the layers were aggregated and re-projected to the geographic attributes of the prediction grid using a bilinear interpolation. As might be expected from the measurement of a relatively invariant covariate, these datasets were highly congruent. At the input data locations the mean absolute difference in altitude between the two datasets was 5.81m, with a Pearson's correlation coefficient of $r = 0.99$. GLOBE was selected for further analysis because a greater percentage of its digital elevation model is estimated from the

highly accurate Digital Terrain Elevation Data (DTED) [23,24] (57.5% in GLOBE vs. 50.0% in GTOPO30 DEM).

GPWv3.0 [25] and GRUMP v1 [26]

Gridded population counts with a consistently processed global coverage are available from the Gridded Population of the World dataset (GPWv3.0), and the subsequent Gridded Rural Urban Mapping Project (GRUMPv1.0) which build upon GPW and refine and improve it for current population estimates. Unlike in Hay et al. [27], where the GRUMP population count data were used, we used the GPW population counts as our reference population from which to back calculate to 1975. The arguments for using GRMUP over GPW were that, firstly, the data are available at a finer spatial resolution (1km in GRMUP vs. 5km in GPW), and secondly, the GRMUP data are constructed using a fine-scale areal weighting, based predominantly on night-time lights data collected by the National Oceanic and Atmospheric Administration (NOAA). The result of this areal weighting is that it better clusters persons into known settlements and sparsely populated, large administrative zones, where there is little data to inform the spatial distribution of populations. A limitation of this approach, however, is that it may lead to underestimation of the rural population of West Africa in 1975 using the method of back-calculation applied here (see below). These calculations assumed a constant rate of rural–urban migration, and the use of night-light data measured after 1975—when increasing urban population sizes would make a greater contribution to night-time light signals—could have inflated the population count moved to urban areas, in turn decreasing the rural population count obtained after applying the back calculation to 1975 population levels.

In order to back calculate population counts to 1975 totals, country-specific, 5-yearly average growth rates, g_r, were applied on a per-pixel basis. The growth rates were taken from the World Populations Prospects: 2012 Revision [28]. The formula applied for a given pixel, p, with country-specific average growth rates for the periods 1985–1990 (g_1), 1980–1985 (g_2) and 1975–1980 (g_3) was $p_{75} = p_{90} \times \prod_{r=1}^{3} \exp(-g_r t)$, with p_{75} and p_{90} being, respectively, the required population count

for a given pixel in 1975, and the known population count for that pixel in 1990 (the earliest observed data), with $t = 5$ years. Growth rates are expressed as values per one hundred population (e.g. a growth rate of 0.05 would be a 5% growth rate for the 5-year period). Because microfilarial prevalence in the OCP is reported for the population aged 5 years and above, The World Population Prospects: 2012 Revision data on age composition [28] in 1975 was used to remove the proportion of the p_{75} population attributed to the 0–4 years age class. The country-specific growth rates, the value by which the 1990 population was multiplied, and proportion of the population aged 0–4 years are given in Table A below.

Table A. Population growth rates, factor by which the 1990 population count was multiplied for back-calculation to 1975, and proportion of the population aged 0-4 years for OCP countries.

Country	g_1	g_2	g_3	$\prod_{r=1}^{3} \exp(-g_r t), t = 5$	Proportion of the population aged 0-4 years
Benin	0.026	0.028	0.031	0.654	0.179
Burkina Faso	0.021	0.025	0.026	0.698	0.179
Ivory Coast	0.045	0.041	0.035	0.546	0.195
Ghana	0.019	0.033	0.028	0.670	0.184
Guinea	0.007	0.024	0.034	0.723	0.166
Guinea Bissau	0.010	0.021	0.022	0.767	0.206
Mali	0.017	0.019	0.015	0.775	0.174
Niger	0.028	0.028	0.029	0.654	0.201
Senegal	0.026	0.029	0.031	0.651	0.19
Sierra Leone	0.024	0.024	0.024	0.698	0.178
Togo	0.024	0.036	0.030	0.638	0.189

Country-specific medium-variant intercensal growth rates, g_r, were obtained from the UNDP World Population Prospects: 2012 Revision [28]; g_1 refers to the growth rate for 1985–1990, g_2 to that for 1980–1985 and g_3 to that for 1975–1980. The expression $\prod_{r=1}^{3} \exp(-g_r t), t = 5$ is the overall reduction applied to each p_{90} pixel population count in that country in 1990 to obtain the p_{75} pixel population count in 1975.

Text D. Maps of environmental covariates (β_j)

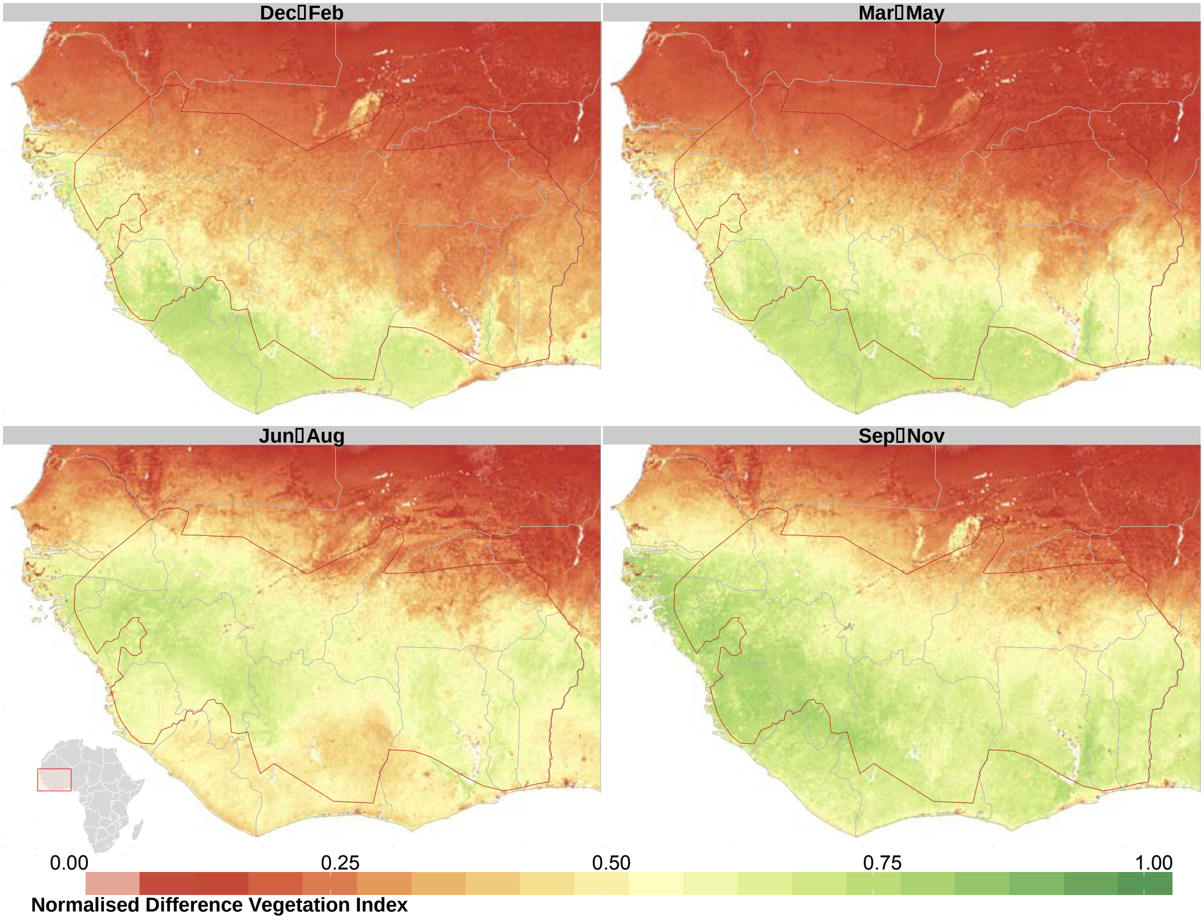

Fig F.1. Maps for covariate values at prediction locations, for NDVI β_j covariates included in the final predictive model. Each panel depicts the mean NDVI value per ecological quarter, labelled in the strip legend above each panel. Inset in the bottom left panel (Jun–Aug) is a map of the study area highlighted in red.

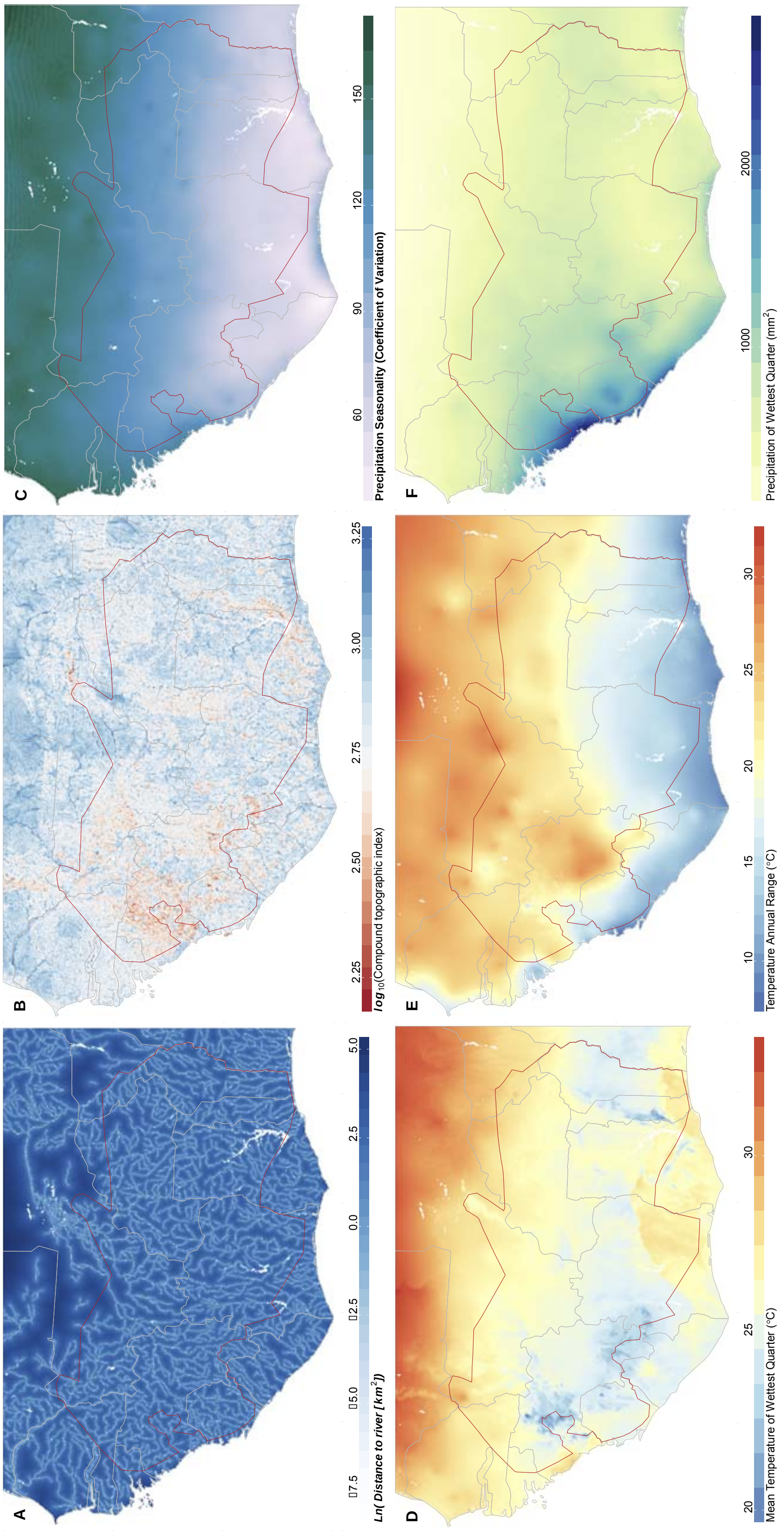

Fig F.2. Maps for covariate values at prediction locations, for all other β_j covariates included in the final predictive model. Maps for Compound Topographic Index (CTI, panel F.2.B) and BIO16 (Precipitation of wettest quarter, panel F.2.F) are plotted on a log-scale for display purposes, but entered into the model as un-transformed variables.

Text E. *K*-folds model validation

For the purposes of model validation, a *K*-folds cross-validation exercise was carried out as described in the Main Text section entitled *Model Validation*. In each of the 10 *K*-folds, the data were randomly split into 90% training data and 10% hold-out validation data. The locations of the hold-out points in each fold are shown in Fig G below. We used the default built-in random number generator (RNG) in R (the *Mersenne-Twister* algorithm [29]) to generate indices to split the data by using the function *sample( )*. The starting state of the RNG was pre-specified by setting a randomly chosen initial seed value ($seed = 5242675$) for the purposes of reproducibility. The row order of the database was then randomised using the command: $sample(737)$. For fold $K = 1$ rows 1:74 from this re-ordered database were used as the hold-out set, and all others as training data; for $K = 2$ rows 75:148 were used as validation data and all other rows as training data, and so on, for $K = 1,...,10$.

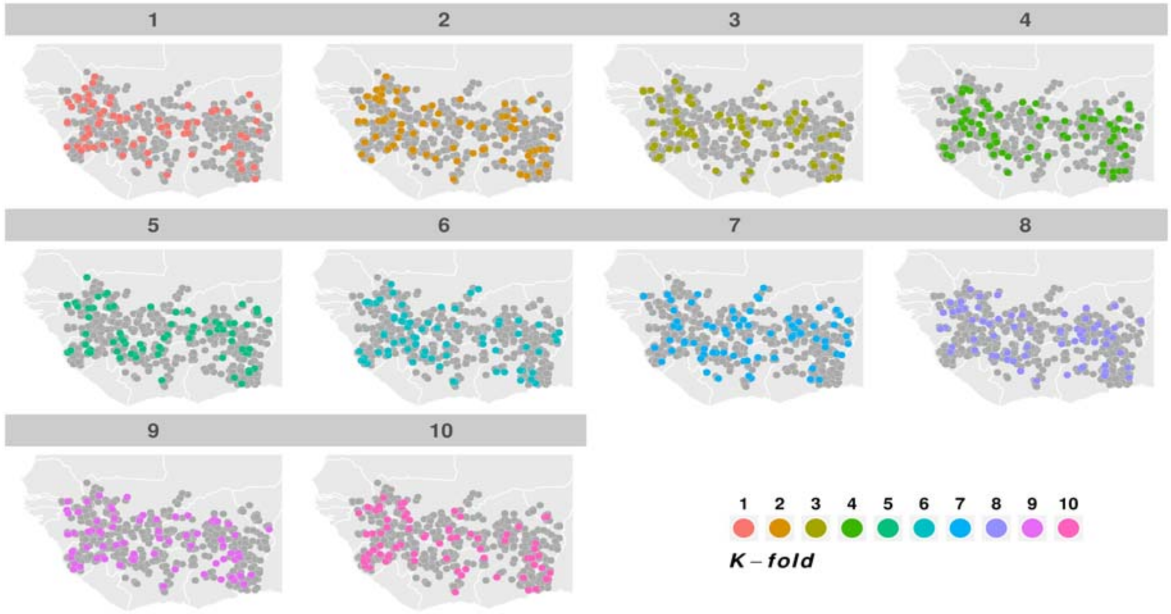

Fig G. The location of validation points in each *K*-fold cross-validation dataset. The coloured data points correspond to the 10% hold-out validation data; the grey markers are the remaining 90% training data.

Text F. MCMC chain diagnostics

Chains were initially run with different starting parameters to ensure good mixing of chains, and to estimate the required length of burn-in period that would be discarded. Both time to convergence and burn-in period are related to the initial set of parameters chosen; additionally, parameter step size was updated at each iteration of the model. Most parameters converged after just 5,000 model iterations from very different starting values ($\beta_j = \{-5, 1, 5\}$) (Fig H below). However, some parameters took longer to converge; for instance, NVDI for the second ecological quarter required roughly 1×10^5 iterations (Fig H, panel labelled $NDVI_{Q2}$—only first 5,000 iterations shown), and the range parameter, ϕ, took approximately 3×10^5 iterations to converge from an initial set of $\phi = \{1, 50, 100\}$ (Fig I). Therefore, a long burn-in period of 1×10^6 iterations was set, which was subsequently discarded to ensure sampling from the posterior distribution.

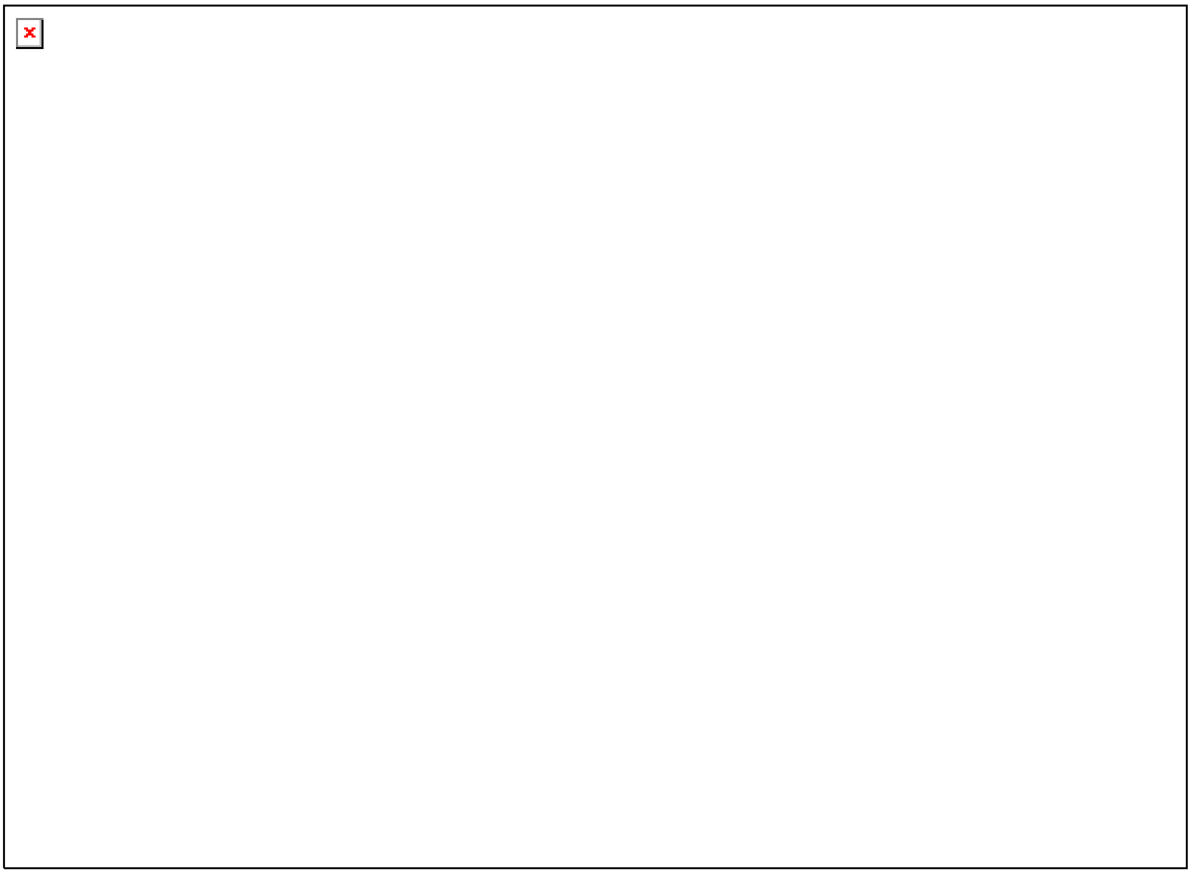

Fig H. MCMC traceplots of all β_j regression coefficients. The plots show the value of each regression coefficient (on the *y*-axis) at each iteration (on the *x*-axis) of the MCMC fitting process, for three independent chains with different starting values. Posterior parameter values are presented in Table 3 of the Main Text. Only 5,000 iterations (as most parameters had converged) are illustrated.

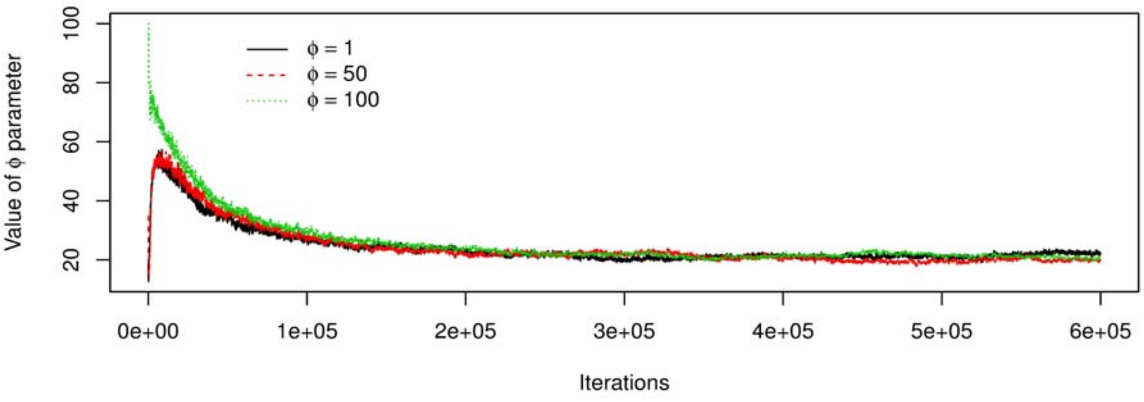

Fig I. MCMC traceplot of the value of the range parameter, ϕ. Three independent chains are illustrated, with starting values of $\phi = 1$, $\phi = 50$ and $\phi = 100$. Starting values were deliberately chosen to be far apart to test the robustness of the fitting process. Convergence of this parameter was achieved after approximately 3×10^5 iterations.

Text G. Posterior distributions of model parameters

The mean values and Bayesian credible intervals (95% and 75% BCIs) of the β_j regression coefficients for each of the environmental covariates and parameters governing the spatial covariance structure, ϕ and σ^2, are given in Table 3 of the Main Text. Plots of the highest posterior density (HPD) are shown in Fig J, below. The HPD for a given probability value, $x\%$, is the shortest interval over the posterior distribution in which $x\%$ of the distribution falls. The HPD intervals were calculated using the package *LaplacesDemon Version 13.3.4* in R (3.0.1) [30].

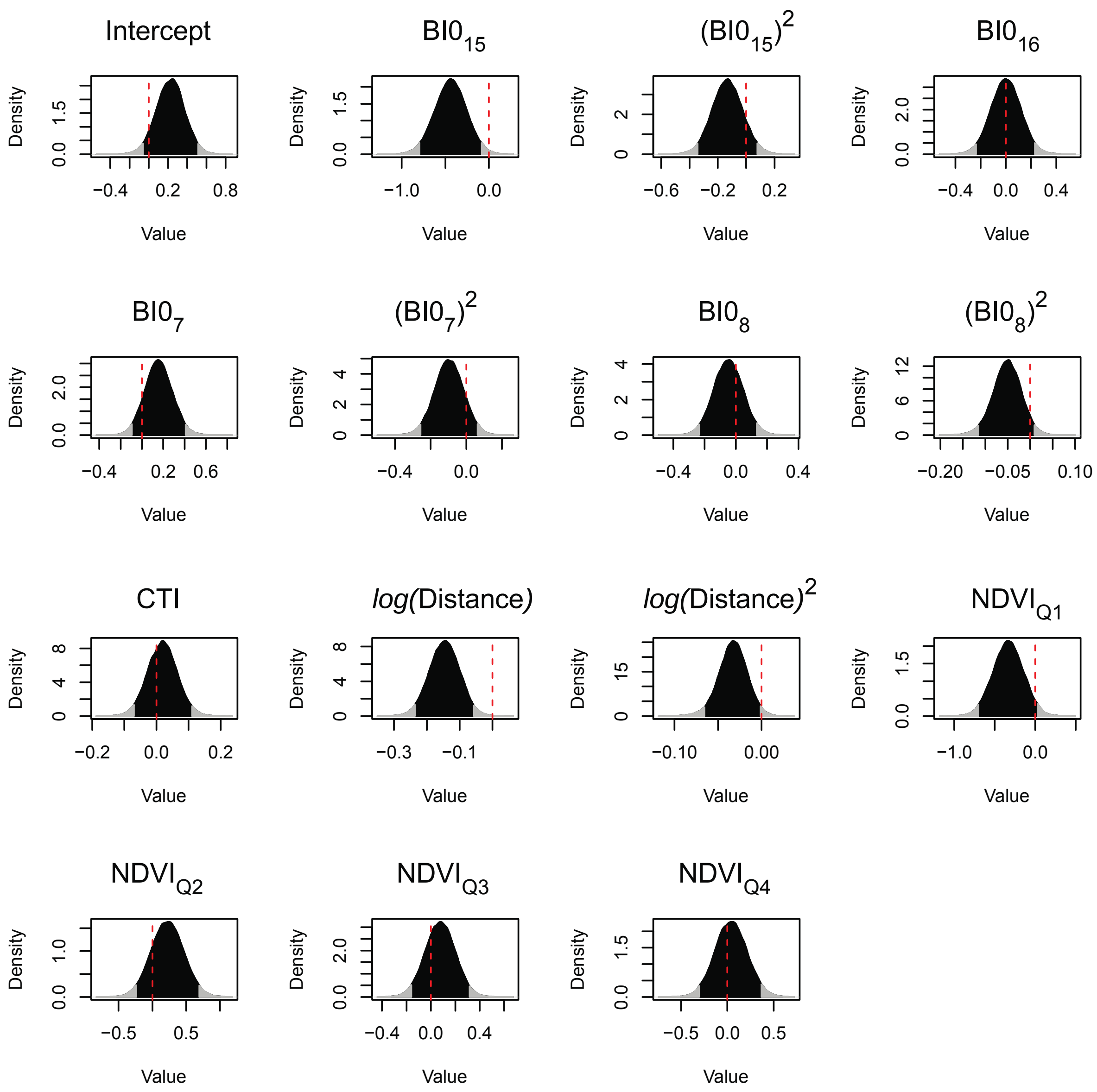

Fig J. Highest posterior density (HPD) plots of model parameters. The dark shaded regions contain 95% of the posterior distribution for each of the β_j (environmental covariate) model parameters summarised in Table 3 of the Main Text. The vertical red-dashed line crosses the *x*-axis at 0 (i.e. no statistically significant effect). (NB The notation **I(function)** in R (the program used to prepare these plots) corresponds to the exact operation stated within the brackets.)

Text H. Further maps derived from the predictive posterior distributions

Among the many advantages of the Bayesian approach to geostatistical mapping, is the use of the posterior distribution to obtain other quantities of interest from the model in addition to measures of central tendency .However, this also poses the challenge of how best to summarise the information that becomes available; e.g., 112,295 histograms at each prediction location would not be particularly useful.

In the Main Text, we summarised the mean behaviour of the posterior predictive distributions for microfilarial prevalence by taking the mean, $\overline{\mu}_i$ of the distribution at each of the prediction locations. For the purposes of visual display, the mean was plotted by binning it into 10%-wide intervals of prevalence, because it is easier to see contiguous areas with similar prevalence values. However, this necessarily erodes some of the fine detail in the maps. Fig K below presents the point estimate for $\overline{\mu}_i$ at each prediction location, plotted on a continuous scale. In this visualisation it is possible to see the effect of the environmental covariate, log(*Distance to river*). The stream-lines network (visible in Fig F2.A) is visible (albeit faintly) in this map, showing the importance of this covariate to the overall model.

Another property of interest is the signal to noise ratio (SNR). More often connected with the communication and electronics industries, the SNR gives the ratio of signal strength (mean microfilarial prevalence) to noise (dispersion in the posterior distribution, as measured by the standard deviation). Pixels with more dispersion in the posterior distribution have a lower SNR. In Fig L below the highest SNR values are within the OCP area, where the ground-truth data are located, as would be expected from a geostatistical model that has a dependence on Euclidean distance.

We could also choose a different realisation of the posterior to display, e.g. the median of each distribution. Since we assume a normal error distribution at each posterior predictive location, the values for the mean and median would ideally be identical. Fig M shows a histogram of the values of the mean prevalence value (left panel) and median prevalence value (right panel) for all 112,295

prediction locations. These distributions are highly similar, suggesting that the assumed normal distribution is a good fit for the posterior predictive distributions.

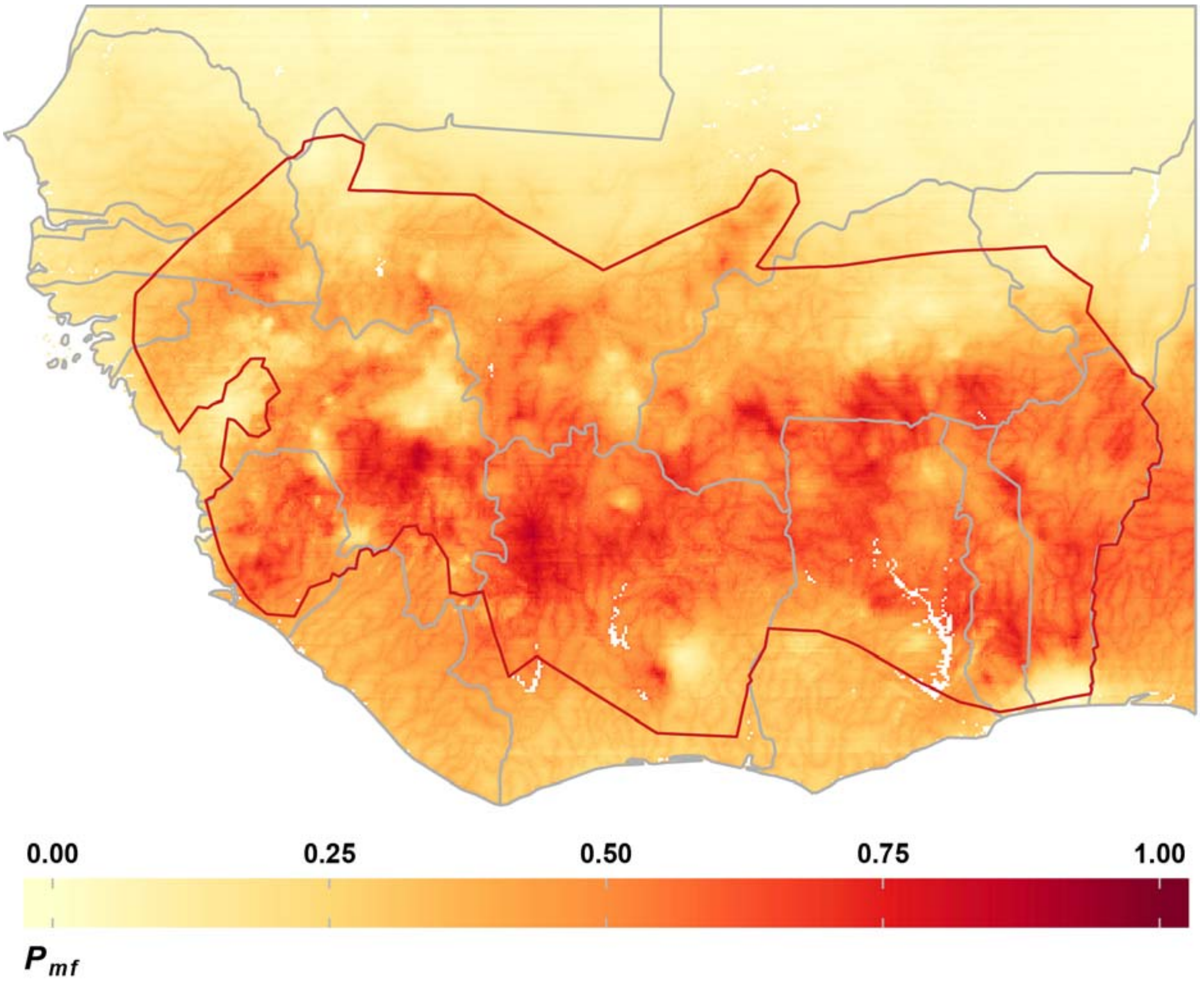

Fig K. Mean of predictive posterior distributions for estimated microfilarial prevalence of *Onchocerca volvulus* prior to the commencement of the Onchocerciasis Control Programme in West Africa (OCP) in 1975. Model outputs are presented and plotted on a continuous prevalence scale (compare this visualisation with Fig 3A in Main Text, where output microfilarial prevalence was binned in 10% intervals). The effect of the environmental covariate *log(distance to river)* is even more evident in this visualisation from the faint outline of the stream network appearing in this plot. The thick read border represents the OCP limits.

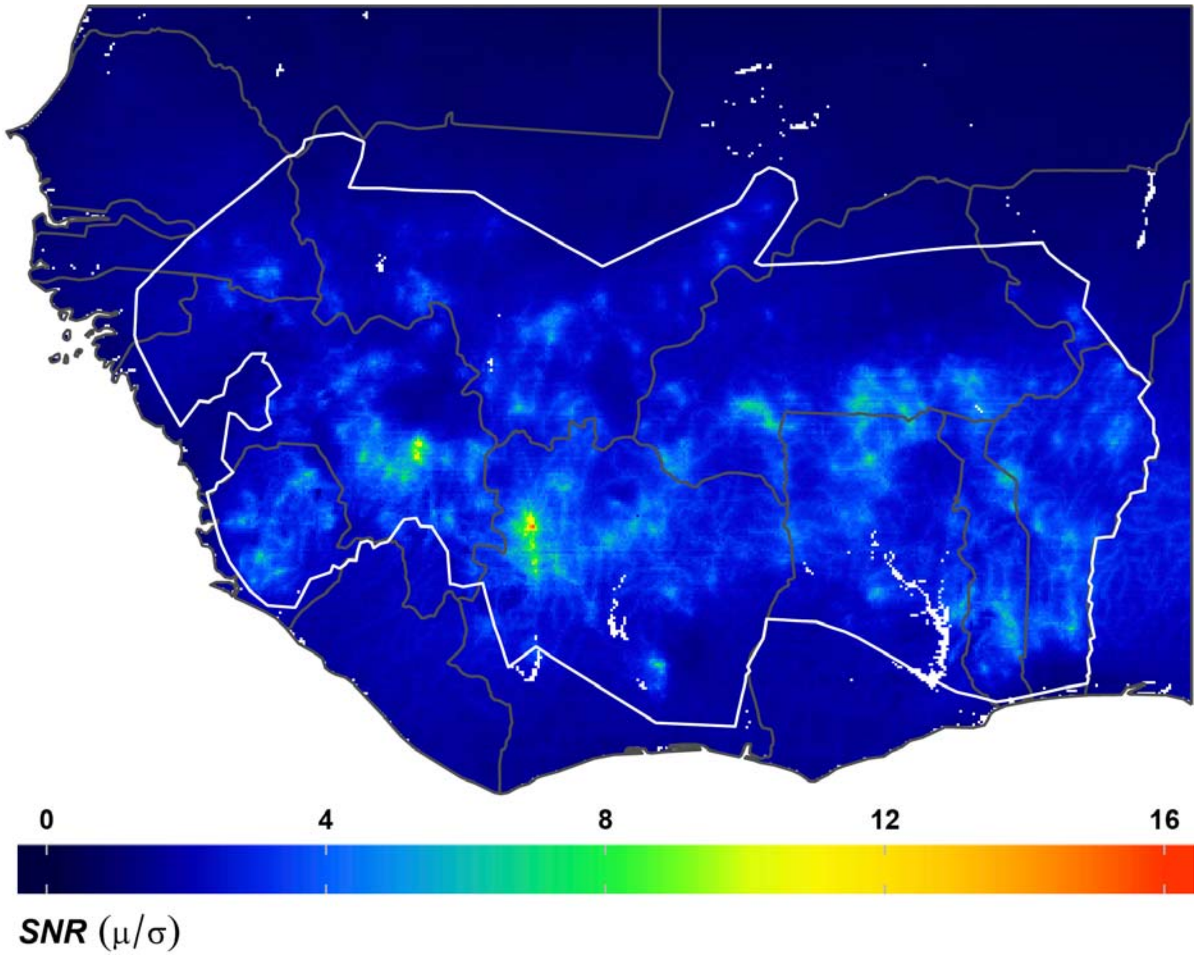

Fig L. Signal to noise ratio (SNR) between mean and standard deviation of predictive posteriors. The strongest SNR values are found in areas close to ground-truth data, within the OCP borders, particularly in meso- and hyperendemic areas. A small cluster of very high prevalence villages between the regions of Bafing and Denguélè in Côte d'Ivoire indicates that all realisations from the predictive posterior are very similar, resulting in a very high SNR in that area (green and yellow area with orange core in Western Côte d'Ivoire).

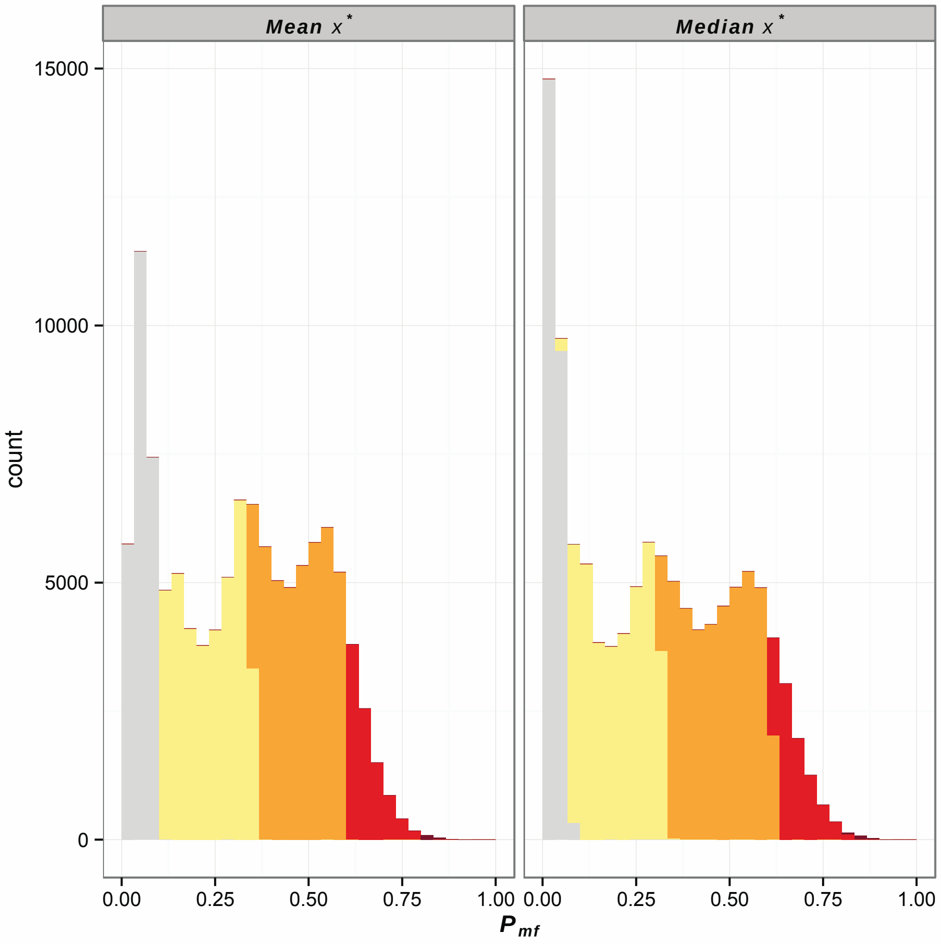

Fig M. Histogram of model output mean (left) and median (right) microfilarial prevalence values at all prediction locations. The data are stratified by endemicity class. The similarity between the frequency distributions of both summary metrics suggests that the assumption of a normal distribution at each of the predictive posteriors is reasonable.

Text I. Geospatial software

All spatial data and geostatistical modelling were handled in *R 3.0.1* [31]. The main packages used to handle the spatial data were: *raster 2.2.12* [32] for manipulation of raster data; *sp 1.0.14* [33,34] for manipulation of vector shapefiles (polygons, lines and points); *rgdal 0.8.14* [35] for providing bindings to the Geospatial Data Abstraction Library (GDAL) [36]; *rgeos 0.3.2* [37] for providing bindings to Geometry Engine – Open Source (GEOS); *gpplot2 0.9.3.1* [38] for plotting of all data; *gridExtra 0.9.1* [39] for further manipulation of plots; *data.table 1.8.11* [40] for manipulating large datasets generated by this analysis and *RColorBrewer 1.0.5* [41] for map colour palettes. The vector shapefile data for drawing country outlines was obtained from Natural Earth (www.naturalearthdata.com), a source of free public-domain vector and raster map data.

References

1) Hougard JM, Alley ES, Yaméogo L, Dadzie KY, Boatin BA (2001) Eliminating onchocerciasis after 14 years of vector control: a proved strategy. J Infect Dis 184(4): 497–503.

2) Basáñez MG, Pion SDS, Boakes E, Filipe JAN, Churcher TS, Boussinesq M (2008) Effect of single dose ivermectin on *Onchocerca volvulus*: a systematic review and meta-analysis. Lancet Infect Dis 8(5): 310–322.

3) Moreau JP, Prost A, Prod'hon J (1978) Essai de normalisation de la méthodologie des enquêtes clinico-parasitologiques sur l'onchocercose en Afrique de l'ouest. Méd Trop (Mars) 38(1): 43–51.

4) Prost A, Thylefors B, Pairault C (1975) Methods of mass epidemiological evaluation of onchocerciasis. Their utilisation in a vector control programme. Geneva: World Health Organization. ONCHO/WP/75.14 ONCHO/WP/75.14. Available: [http://whqlibdoc.who.int/hq/pre-wholis/ONCHO_WP_75.14.pdf]. Accessed 23 June 2015.

5) Prost A, Prod'hon J (1978) Le diagnostique de l'onchocercose. Revue critique des méthodes en usage. Méd Trop (Mars) 38(5): 519–532.

6) Hijmans RJ, Cameron SE, Parra JL, Jones PG, Jarvis A (2005) Very high resolution interpolated climate surfaces for global land areas. Int J Climatol 25(15): 1965–1978.

7) Danielson JJ (1996) Delineation of drainage basins from 1 km African digital elevation data. In: Pecora Thirteen, Human Interactions with the Environment – Perspectives from Space. Sioux Falls, South Dakota, August 20–22, 1996.

8) Burt TP, Butcher DP (1985) Topographic controls of soil-moisture distributions. J Soil Sci 36(3): 469–486.

9) Beven KJ, Kirkby MJ (1979) A physically based variable contributing area model of basin hydrology. Hydrol Sci Bull 24(1): 43−69.

10) NASA Land Processes Distributed Active Archive Center (LP DAAC) (2001) MODIS MOD13A1. USGS/Earth Resources Observation and Science (EROS) Center, Sioux Falls, South Dakota. Available: [https://lpdaac.usgs.gov/products/modis_products_table]. Accessed 23 June 2015.

11) NASA Land Processes Distributed Active Archive Center (LP DAAC) (2001) MODIS MOD1A2. USGS/Earth Resources Observation and Science (EROS) Center, Sioux Falls, South Dakota. Available: [https://lpdaac.usgs.gov/products/modis_products_table]. Accessed 23 June 2015.

12) NASA Land Processes Distributed Active Archive Center (LP DAAC) (2001) MODIS MCD12Q1. USGS/Earth Resources Observation and Science (EROS) Center, Sioux Falls, South Dakota. Available: [https://lpdaac.usgs.gov/products/modis_products_table]. Accessed: 23 June 2015.

13) Lambin EF, Geist HJ (eds) (2006) *Land-Use and Land-Cover Change. Local Processes and Global Impacts*. The IGBP Series. Berlin: Springer-Verlag, 222 pp.

14) Tucker CJ, Pinzon JE, Brown ME (2004) Global Inventory Modeling and Mapping Studies, Normalized Difference Vegetation Index (NDVI), 2.0, Global Land Cover Facility, University of Maryland, College Park, Maryland, 01/07/1981–31/12/1986.

15) Pinzon JE, Brown ME, Tucker CJ (2005) EMD correction of orbital drift artifacts in satellite data stream. In: Huang NE, Shen SSP, editors. *Hilbert-Huang Transform and its Applications*. Interdisciplinary Mathematical Sciences Volume 5, Singapore: World Scientific Publishing Co. Pte. Ltd., pp: 167–186.

16) Tucker CJ, Pinzon JE, Brown ME, Slayback D, Pak EW, Mahoney R, Vermote EF, El Saleous N (2005) An extended AVHRR 8-km NDVI data set compatible with MODIS and SPOT vegetation NDVI data. Int J Remote Sens 26(20): 4485–4498.

17) Food and Agriculture Organization of the United Nations. FAO GEONETWORK. Rivers of Africa (Derived from HydroSHEDS) (GeoLayer) (Latest update: 18 Feb 2014). Available: [http://data.fao.org/ref/b891ca64-4cd4-4efd-a7ca-b386e98d52e8.html?version=1.0]. Accessed: 23 June 2015.

18) Strahler AN (1952) Dynamic basis of geomorphology. Geol Soc Am Bull 63(9): 923–938.

19) White F (1983) Vegetation of Africa - a descriptive memoir to accompany the Unesco/AETFAT/UNSO vegetation map of Africa; Natural Resources Research Report XX; U. N. Educational, Scientific and Cultural Organization; 7 Place de Fontenoy, 75700 Paris, France; 356 pages.

20) US Geological Survey (1996) Global 30-Arc-Second Elevation Data Set, Sioux Falls, South Dakota.

21) Gesch DB, Larson KS (1996) Techniques for development of global 1-kilometer digital elevation models. In: Pecora Thirteen, Human Interactions with the Environment--Perspectives from Space, 13th, Sioux Falls, South Dakota, August 20-22, 1996, Proceedings. Bethesda, Maryland: American Society of Photogrammetry and Remote Sensing.

22) GLOBE Task Team and others (1999) In: Hastings DA, Dunbar PK, Elphingstone GM, Bootz M, Murakami H, Maruyama H, Masaharu H, Holland P, Payne J, Bryant NA, Logan TL, Muller JP, Schreier G, MacDonald JS, editors. The Global Land One-kilometer Base Elevation (GLOBE) Digital Elevation Model, Version 1.0. National Oceanic and Atmospheric Administration, National Geophysical Data Center, 325 Broadway, Boulder, Colorado 80305-3328.

23) National Imagery and Mapping Agency (1996) Digital Terrain Elevation Data Level 0. National Imagery and Mapping Agency, Fairfax, Virginia (partly in GLOBE Task Team and others, 1999).

24) NIMA, USGS, and NGDC (1997) 30"-gridded DEM from DTED, Precursors, and Derivatives. National Geophysical Data Center, Boulder, Colorado (in GLOBE Task Team and others, 1999).

25) Center for International Earth Science Information Network (CIESIN), Columbia University; and Centro Internacional de Agricultura Tropical (CIAT) (2005) Gridded Population of the World Version 3 (GPWv3): Population Density Grids. Palisades, NY: Socioeconomic Data and Applications Center (SEDAC), Columbia University. Available: [http://sedac.ciesin.columbia.edu/data/set/gpw-v3-population-density]. Accessed 23 June 2015.

26) Center for International Earth Science Information Network (CIESIN), Columbia University; International Food Policy Research Institute (IFPRI); the World Bank; and Centro Internacional de Agricultura Tropical (CIAT) (2011). Global Rural-Urban Mapping Project, Version 1 (GRUMPv1): Urban Extents Grid. Palisades, NY: Socioeconomic Data and Applications Center (SEDAC), Columbia University. Available: [http://sedac.ciesin.columbia.edu/data/set/grump-v1-urban-extents]. Accessed 23 June 2015.

27) Hay SI, Guerra CA, Gething PW, Patil AP, Tatem AJ, Noor AM, Kabaria CW, Manh BH, Elyazar IR, Brooker S, Smith DL, Moyeed RA, Snow RW. (2009) A world malaria map: *Plasmodium falciparum* endemicity in 2007. PLoS Med 6(3): e1000048. Erratum in: PLoS Med. 2009;6(10). doi: 10.1371/annotation/a7ab5bb8-c3bb-4f01-aa34-65cc53af065d.

28) United Nations (2013) World Population Prospects: The 2012 Revision, DVD edition. New York: UN Department of Economic and Social Affairs, Population Division. Highlights and advance tables.
Available: [http://esa.un.org/wpp/Documentation/pdf/WPP2012_HIGHLIGHTS.pdf]. Accessed: 23 June 2015.

29) Matsumoto M, Nishimura T. (1998) Mersenne Twister: a 623-dimensionally equidistributed uniform pseudo-random number generator. ACM Trans Model Comput Simul 8(1): 3–30.

30) Statisticat LLC (2013). LaplacesDemon: Complete Environment for Bayesian Inference. CRAN. R package version 13.03.04. Available: [http://cran.r-project.org/web/packages/LaplacesDemon/index.html]. Tutorial available: https://datajobs.com/data-science-repo/Bayesian-Inference-[Statisticat].pdf]. Accessed 23 June 2015.

31) R Core Team (2013). R: A language and environment for statistical computing. R Foundation for Statistical Computing, Vienna, Austria. Available: [http://www.R-project.org/]. Accessed 23 June 2014.

32) Hijmans RJ (2014) raster: raster: Geographic data analysis and modeling. R package version 2.2-12. Available: [http://CRAN.R-project.org/package=raster]. Accessed: 23 June 2015.

33) Pebesma EJ, Bivand RS (2005) Classes and methods for spatial data in R. R News 5(2) [http://cran.r-project.org/doc/Rnews/]. Available: [http://cran.r-project.org/doc/Rnews/Rnews_2005-2.pdf]. Accessed: 23 June 2015.

34) Bivand RS, Pebesma E, Gómez-Rubio V (2013) *Applied Spatial Data Analysis with R*, Second edition. New York: Springer, 405 pp. Available: [http://www.asdar-book.org/]. Accessed: 23 June 2015.

35) Bivand R, Keitt T, Rowlingson B (2013) rgdal: Bindings for the Geospatial Data Abstraction Library. R package version 0.8-14. Available: [http://CRAN.R-project.org/package=rgdal]. Accessed: 23 June 2015.

36) GDAL (2015) GDAL - Geospatial Data Abstraction Library: Version GDAL/OGR 2.0.0 (June 2015), Open Source Geospatial Foundation. Available: [http://www.osgeo.org/gdal/]. Accessed 23 June 2015.

37) Bivand R, Rundel C (2013) rgeos: Interface to Geometry Engine - Open Source (GEOS). R package version 0.3-2. Available: [http://CRAN.R-project.org/package=rgeos]. Accessed 23 June 2015.

38) Wickham H (2009) *ggplot2: Elegant Graphics for Data Analysis*. New York: Springer, 213 pp.

39) Auguie B (2012) gridExtra: functions in Grid graphics. R package version 0.9.1. Available: [http://CRAN.R-project.org/package=gridExtra]. Accessed: 23 June 2015.

40) Dowle M, Short T, Lianoglou S, Srinivasan A (with contributions from Saporta R and Antonyan E) (2013) data.table: Extension of data.frame for fast indexing, fast ordered joins, fast assignment, fast grouping and list columns. R package version 1.8.11/r1001. Available: [http://R-Forge.R-project.org/projects/datatable/]. Accessed: 23 June 2015.

41) Neuwirth E (2011) RColorBrewer: ColorBrewer palettes. R package version 1.0-5. Available: [http://CRAN.R-project.org/package=RColorBrewer]. Accessed: 23 June 2015.
